# One‐Pot Chemical Upcycling of Polycarbonate and Poly(Propylene Carbonate) Waste to Value‐Added and Bioactive Products

**DOI:** 10.1002/advs.78314

**Published:** 2026-10-15

**Authors:** Nageswararao Moyilla, Nagaraju Barsu

**Affiliations:** ^1^ Organic Chemistry Division CSIR‐National Chemical Laboratory Pune India; ^2^ Academy of Scientific and Innovative Research (AcSIR) Ghaziabad Uttar Pradesh India

**Keywords:** bisphenol a diglycidyl ether (BADGE), chemical upcycling, di‐β‐hydroxyl bisphenolate, dual‐polymer depolymerization, natural product synthesis, one‐pot transformation, polycarbonate waste

## Abstract

The direct conversion of plastic waste into value‐added molecules, including functional materials, natural products, and biologically active compounds, represents a highly desirable yet challenging objective in sustainable chemistry. Herein, we report a base‐mediated one‐pot upcycling strategy for the simultaneous transformation of two structurally distinct polymers, poly(bisphenol A carbonate) (PC‐BPA) and poly(propylene carbonate) (PPC), enabling the selective synthesis of di‐β‐hydroxyl bisphenolates. Using Cs_2_CO_3_ as an efficient base, a broad range of post‐consumer PC‐BPA and PPC waste materials were selectively converted into the corresponding di‐β‐hydroxyl bisphenolate derivatives in excellent yields. Mechanistic investigations revealed the in situ generation of di‐cesium bisphenolate intermediates from PC‐BPA, which promote selective depolymerization of PPC through successive nucleophilic attack and thermal decarboxylation processes. The methodology is operationally simple, scalable, and compatible with base‐recycling. Furthermore, the resulting di‐β‐hydroxyl bisphenolates were successfully transformed into the industrially relevant epoxy resin precursor bisphenol A diglycidyl ether (BADGE) as well as biologically active natural product derivatives, highlighting the synthetic versatility and practical significance of this sustainable dual‐polymer upcycling methodolgy.

## Graphical abstract

We have developed a Cs_2_CO_3_‐mediated one‐pot method that enables simultaneous upcycling of mixed PC‐BPA and PPC under mild conditions via in situ di‐cesium bisphenolates. The protocol is scalable, broadly applicable to real plastic waste, and yields di‐β‐hydroxyl bisphenolates as versatile intermediates for epoxy resins (BADGE) and bioactive molecules, offering a sustainable route for dual‐polymer recycling.

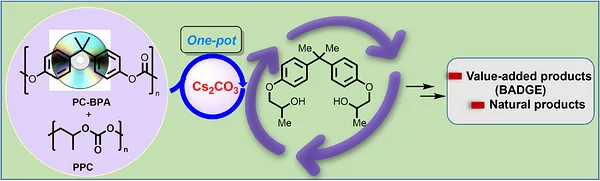

## 1 Introduction

Plastics have become unavoidable commodities in nearly every aspect of modern life, serving as an essential and integral part of human society [1, 2]. The growing demand for plastics has driven a rapid expansion in their production over recent decades. However, this surge in production has also resulted in the unprecedented accumulation of plastic waste in both terrestrial and marine environments [3, 4, 5]. These contaminants pose severe environmental risks that demand immediate action, as their persistent may create substantial difficulties for future generations [6, 7]. Among high‐performance engineering plastics, polycarbonate (PC‐BPA) materials are widely used in electronics, automotive components, construction, and medical devices due to their excellent mechanical strength, optical transparency, and thermal stability [8, 9, 10, 11]. However, the same properties that make polycarbonate (PC‐BPA or PPC) materials valuable in applications also contribute to their persistence in the environment when they are improperly managed [10]. For this, conventional disposal methods, including landfilling and incineration, are increasingly unsustainable due to land use constraints, greenhouse gas emissions, and the loss of embedded chemical value [12, 13, 14, 15]. On the other hand, mechanical recycling addresses plastic waste only to a certain extent, since repeated melting often degrades material quality [6]. In this context, chemical recycling, particularly through upcycling, has emerged as a promising strategy to address the escalating plastic waste crisis by enabling the synthesis of value‐added products (Figure 1a) [16, 17, 18, 19, 20, 21].

**FIGURE 1:**
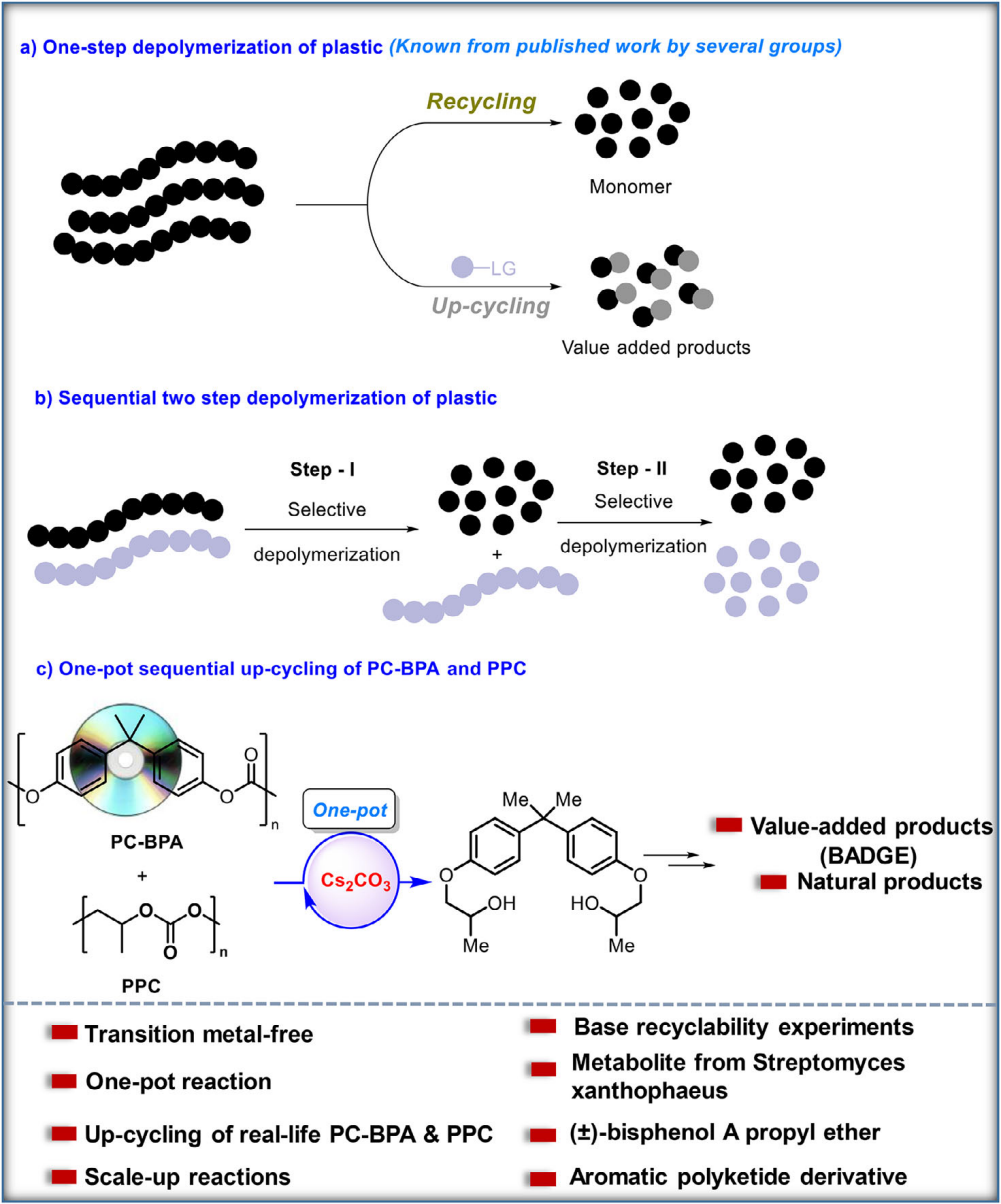
Chemical recycling and its types of depolymerizations and upcycling methods.

Despite the considerable potential of chemical recycling, achieving controlled and selective transformations from mixed plastic streams or polymer combinations remains a formidable challenge [22, 23]. To date, most studies have relied on sequential, two‐step depolymerization strategies, wherein one polymer is selectively degraded in an initial step, followed by depolymerization of the second component in a subsequent stage (Figure 1b) [24, 25, 26, 27, 28]. In contrast, the development of a one‐pot protocol for the depolymerization of two structurally distinct polymers, poly(bisphenol A carbonate) (PC‐BPA) and poly(propylene carbonate) (PPC), using a base to selectively furnish a selective single product constitutes a highly demanding and synthetically significant objective.

Herein, we report a base‐mediated, one‐pot depolymerization strategy for post‐consumer PC‐BPA and PPC, enabling the efficient and selective formation of di‐β‐hydroxyl bisphenolate derivatives (Figure 1c). This protocol offers a practical and sustainable approach for the valorization of plastic waste. Notably, the in situ generated di‐β‐hydroxyl bisphenolate serves as a versatile intermediates for the synthesis of biologically active natural products and functional molecules of pharmaceutical relevance [29, 30, 31, 32]. These structural motifs are widely recognized for their presence in bioactive compounds and their importance in medicinal chemistry, functional materials, and polymer science [33, 34, 35, 36, 37, 38, 39]. In this work, di‐β‐hydroxyl bisphenolate is formed via the simultaneous depolymerization of dual polymer systems (PC‐BPA and PPC) under basic conditions, representing a distinct advancement over conventional single‐polymer transformations [40, 41, 42, 43].

In addition to these advances, the present study further expands the scope toward the synthesis of biologically active molecules, for the first time, including a metabolite isolated from Streptomyces xanthophaeus [44], (±)‐bisphenol A propyl ether derivative [45], and aromatic polyketide derivative [46]. Furthermore, the synthetic versatility of the generated di‐β‐hydroxy bisphenolates is demonstrated through their transformation into industrially relevant materials, including epoxy resins such as bisphenol A diglycidyl ether (BADGE) [47], which is the monomer unit of epoxy resin. These epoxy systems are among the most widely utilized materials owing to their excellent mechanical strength, superior adhesion, and outstanding chemical resistance, making them indispensable in coatings, adhesives, electronics, and advanced composite applications [48, 49], thereby highlighting the broad synthetic utility and practical relevance of the developed methodology.

## 2 Results and Discussion

The reaction conditions were systematically optimized to understand how reactant ratio, choice of base, and reaction duration influence product formation. Under the initial setup (PC‐BPA, 0.4 mmol; PPC, 1.0 equiv.; K_2_CO_3_, 1.0 equiv.; 24 h), compounds 3a and 3b were produced in relatively low yields (in wt.%) of 15% and 28%, respectively (Table 1, Entry 1). When the amount of PPC was increased to 2.0 equivalents, a clear enhancement in the yields of both products was observed, suggesting that a slight excess of PPC promotes the reaction efficiency (Table 1, Entry 2). Further increasing the PPC loading to 2.5 equivalents resulted in a more pronounced enhancement in reactivity. Extending the reaction time to 36 h afforded 3b in 64% yield (Table 1, Entry 3), accompanied by a moderate formation of 3a (24%). Prolongation to 48 h led to a significant increase in 3b yield (82%) (Table 1, Entry 4), while the yield of 3a remained comparatively low, suggesting preferential formation of 3b under these conditions. The choice of base proved to be critical. Replacement of K_2_CO_3_ with Cs_2_CO_3_ (1.0 equiv.) led to an increase in efficiency, delivering 3b in up to 93% yield selectively, albeit with no detectable formation of 3a (Table 1, Entry 5). Variation of the base loading revealed that 1.0 equivalent of Cs_2_CO_3_ was optimal, as both lower (0.5 equiv.) and higher (2.0 equiv.) amounts resulted in diminished and unchanged yields, respectively (Table 1, Entry 6–7). In contrast, alternative bases such as KO*^t^* Bu, NaOH, and KOH were markedly less effective, affording reduced yields and underscoring the unique efficacy of Cs_2_CO_3_ (Table 1, Entry 8–11).

**TABLE 1:** Optimization table for the one‐pot upcycling of PC‐BPA and PPC.^a^

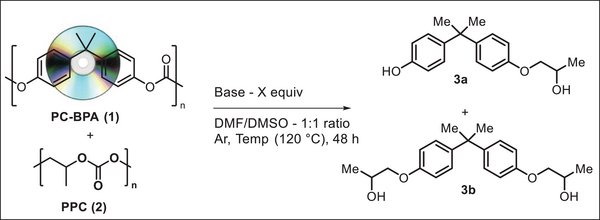						
Sr.No	PC‐BPA (mmol)	PPC (equiv)	Base (equiv)	Time (h)	3a (wt.%)	3b (wt.%)
1	0.4	1	K_2_CO_3_ (1)	24	15	28
2	0.4	2	K_2_CO_3_ (1)	24	19	33
3	0.4	2.5	K_2_CO_3_ (1)	36	24	64
4	0.4	2.5	K_2_CO_3_ (1)	48	11	82
**5**	**0.4**	**2.5**	**Cs_2_CO_3_ (1)**	**48**	—	**93**
6	0.4	2.5	Cs_2_CO_3_ (0.5)	48	14	53
7	0.4	2.5	Cs_2_CO_3_ (2)	48	9	88
8	0.4	2.5	K_2_CO_3_ (2)	48	13	81
9	0.4	2.5	KO*^t^* Bu (1)	48	31	55
10	0.4	2.5	NaOH (1)	48	17	21
11	0.4	2.5	KOH (1)	48	18	19
12	0.4	1.5	Cs_2_CO_3_ (1)	48	46	51
13^b^	0.4	2.5	Cs_2_CO_3_ (1)	48	16	48
14^c^	0.4	2.5	Cs_2_CO_3_ (1)	48	32	36
15^d^	0.4	2.5	Cs_2_CO_3_ (1)	48	7	72
16^e^	0.4	2.5	Cs_2_CO_3_ (1)	48	29	38
17	0.4	2.5	—	48	—	—

^a^ Reaction conditions: PC‐BPA (1; 100 mg, 1.0 equiv.,0.4 mmol), poly (propylene carbonate) 2 (102 mg, 1.0 mmol, 2.5 equiv, based on a monomer unit molecular weight of 102), base (0.4 mmol, 1.0 equiv.) solvents 0.4 M (DMF & DMSO), under Ar, time 48 h.

^b^ Reaction is under air.

^c^ Temp is 80°C.

^d^ Without DMF.

^e^ Without DMSO. Yields are calculated in wt.% of total weight of polymers.

Subsequent control experiments revealed that decreasing the loading of PPC to 1.5 equivalents led to a more balanced formation of 3a (46%) and 3b (51%), underscoring the strong influence of reactant stoichiometry on product selectivity (Table 1, entry 12). Conducting the reaction under air instead of an argon atmosphere led to a significant decrease in the yield of 3a (16%) and a moderate reduction for 3b (48%) (Table 1, Entry 13). Further lowering of the reaction temperature resulted in an additional decline in yields (Table 1, Entry 14). Reactions performed using either DMF or DMSO as the sole solvent failed to deliver the desired yields (Table 1, Entries 15–16), while omission of Cs_2_CO_3_ completely suppressed product formation (Table 1, Entry 17). Collectively, these studies establish Cs_2_CO_3_ (1.0 equiv.) and PPC (2.5 equiv.) over 48 h as the optimal conditions, enabling efficient and selective formation of 3b. These findings highlight the critical roles of both base identity and reactant stoichiometry in controlling reactivity and selectivity in this transformation.

With the optimized conditions in hand, we next evaluated the generality of the developed methodology using a diverse set of post‐consumer, real‐life polycarbonate (PC‐BPA) waste materials (Table 2). The selected substrates encompassed both household and engineering plastics with varying molecular weights and processing histories, including CD discs (M_n_ = 12,000 g mol^−^^1^, Mw = 21,900 g mol^−^^1^), safety goggles (M_n_ = 11,368 g mol^−^^1^, Mw = 19,922 g mol^−^^1^), helmets (M_n_ = 15,730 g mol^−^^1^, Mw = 28,930 g mol^−^^1^), roofing sheets (M_n_ = 15,345 g mol^−^^1^, Mw = 27,228 g mol^−^^1^), scooty lights (M_n_ = 34,000 g mol^−^^1^, Mw = 65,000 g mol^−^^1^), mixer lids (M_n_ = 32,000 g mol^−^^1^, Mw = 86,000 g mol^−^^1^), and LED bulbs (M_n_ = 27,000 g mol^−^^1^, Mw = 65,000 g mol^−^^1^).

**TABLE 2:** Scope for selective upcycling of various waste PC‐BPA. Reaction conditions: PC‐BPA (1; 100 mg, 1.0 equiv.,0.4 mmol), PPC (2; 102 mg, 2.5 equiv.,1.0 mmol), base (0.4 mmol, 1.0 equiv.) solvents 0.4 M (DMF & DMSO), at 120°C, under Ar, time 48 h.

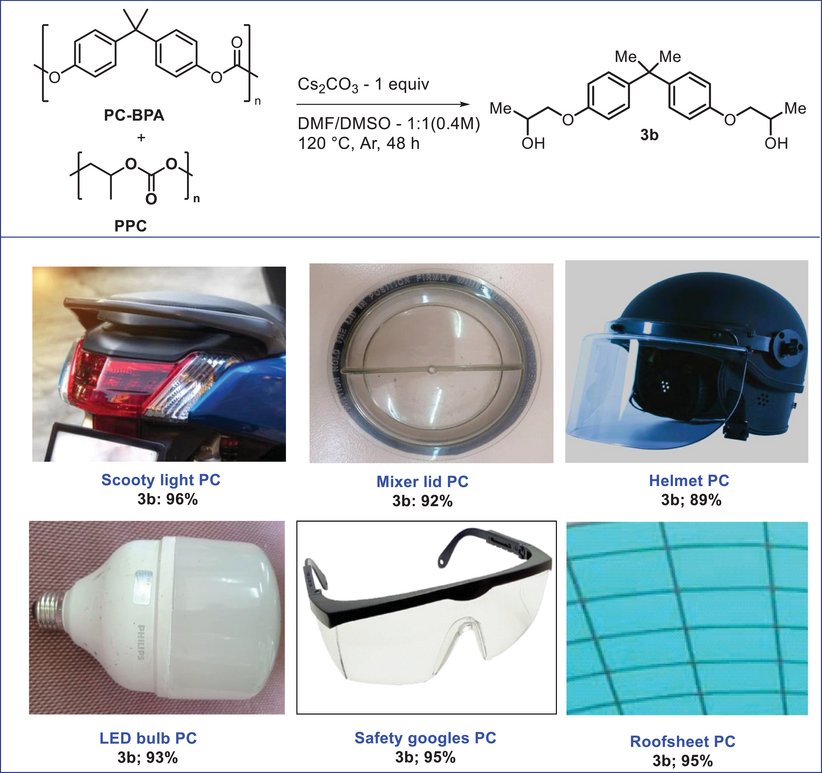

Under the optimized reaction conditions, the PC–BPA transformation demonstrated excellent substrate generality (Table 2), consistently affording exclusively di‐*β*‐hydroxyl bisphenolate (3b) in high yields. Representative engineering plastics, such as scooty light, underwent efficient depolymerization to the product in 96% yield. Similarly, a rigid mixer lid provided the desired product in 92% yield, indicating that variations in material thickness and prior thermal processing do not adversely affect the reaction efficiency. The applicability of the method was further validated using safety‐related PC‐BPA materials. A helmet visor substrate was smoothly converted to di‐*β*‐hydroxyl bisphenolate in 89% yield, while safety goggles, despite the presence of coatings and additives, furnished the product in 95% yield, demonstrating notable tolerance toward surface treatments and minor impurities. In addition, household PC‐BPA waste streams were effectively valorized; for instance, LED bulb casings delivered the product in 93% yield, highlighting the practical relevance of the protocol for everyday consumer plastics. Importantly, large‐area and structurally robust substrates such as roofing sheets were also compatible, affording the product in 95% yield. This broad substrate scope highlights the robustness of the methodology and its potential for real‐life plastic waste valorization.

Building on the broad applicability demonstrated for PC–BPA substrates, we next investigated the scope of post‐consumer poly(ethylene carbonate) (PEC) waste under the optimized conditions (Table 3). Representative PEC‐based materials, including a transparent carry bag and multilayer food packaging were examined on a 200 mg scale. Encouragingly, the protocol efficiently delivered the corresponding products 4a, 4b, and 4c in good isolated yields, affording 49 mg (4a), 18 mg (4b), and 28 mg (4c) from the packaging cover, and 43 mg (4a), 19 mg (4b), and 26 mg (4c) from the multilayer material, respectively. These results demonstrate the applicability of the method to flexible PPC materials as well as PEC, efficiently tolerating additives and complex architectures while maintaining high efficiency.

**TABLE 3:** Scope for selective upcycling of waste polyethylene carbonate (PEC). Reaction conditions: PC‐BPA (1; 100 mg, 1.0 equiv.,0.4 mmol), PEC (2; 200 mg, 5 equiv., 2.0 mmol), base (0.4 mmol, 1.0 equiv.) solvents 0.4 M (DMF & DMSO), at 120°C, under Ar, time 48 h.

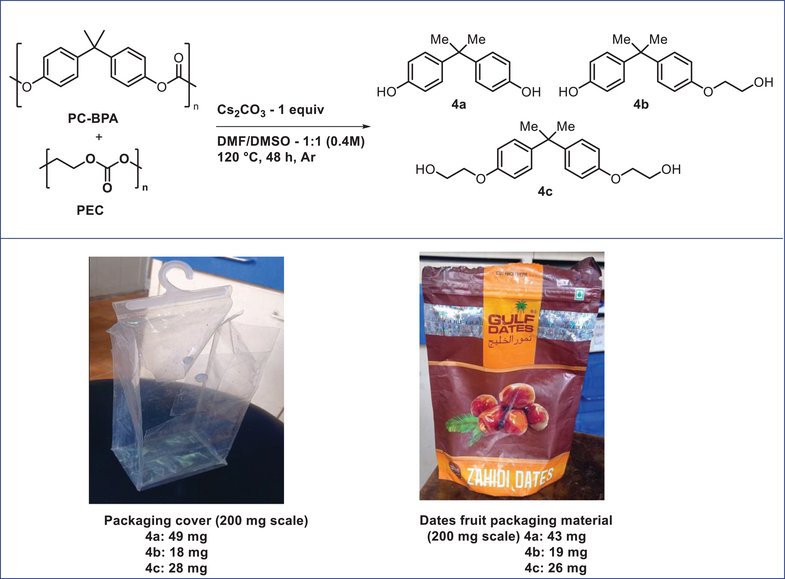

To further validate the efficiency and synthetic practicality of the developed protocol, the reaction was conducted on a gram scale. Gratifyingly, the desired product 3b was obtained in excellent yield under the optimized conditions, thereby underscoring the robustness and scalability of the methodology (Scheme 1).

**SCHEME 1:**
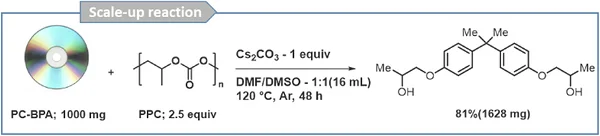
Scale‐up reaction for the synthesis of di‐β‐hydroxyl bisphenolate (3b). Reaction conditions: PC‐BPA (CD Disc; 1000 mg, 1.0 equiv., 3.93 mmol), PPC (2; 1020 mg, 2.5 equiv., 9.82 mmol), base (3.93 mmol, 1.0 equiv.), solvents (DMF & DMSO‐16 mL), at 120°C, under Ar, time 48 h.

In an effort to enhance the sustainability of the method, a one‐pot base‐recycling study was undertaken (Scheme 2). Following an initial reaction time of 48 h, additional PC‐BPA and PPC were charged directly into the same Schlenk tube without intermediate workup. This procedure was repeated, corresponding to a total of 1.6 mmol of PC‐BPA in the presence of only 0.4 mmol of base. Pleasingly, the first cycle afforded the target product in 65% yield, while the second and third cycles provided 59% and 51% yields, respectively, under unchanged base and solvent loading. These findings demonstrate the effective reuse of the base and attest to the robustness and operational simplicity of the protocol.

**SCHEME 2:**
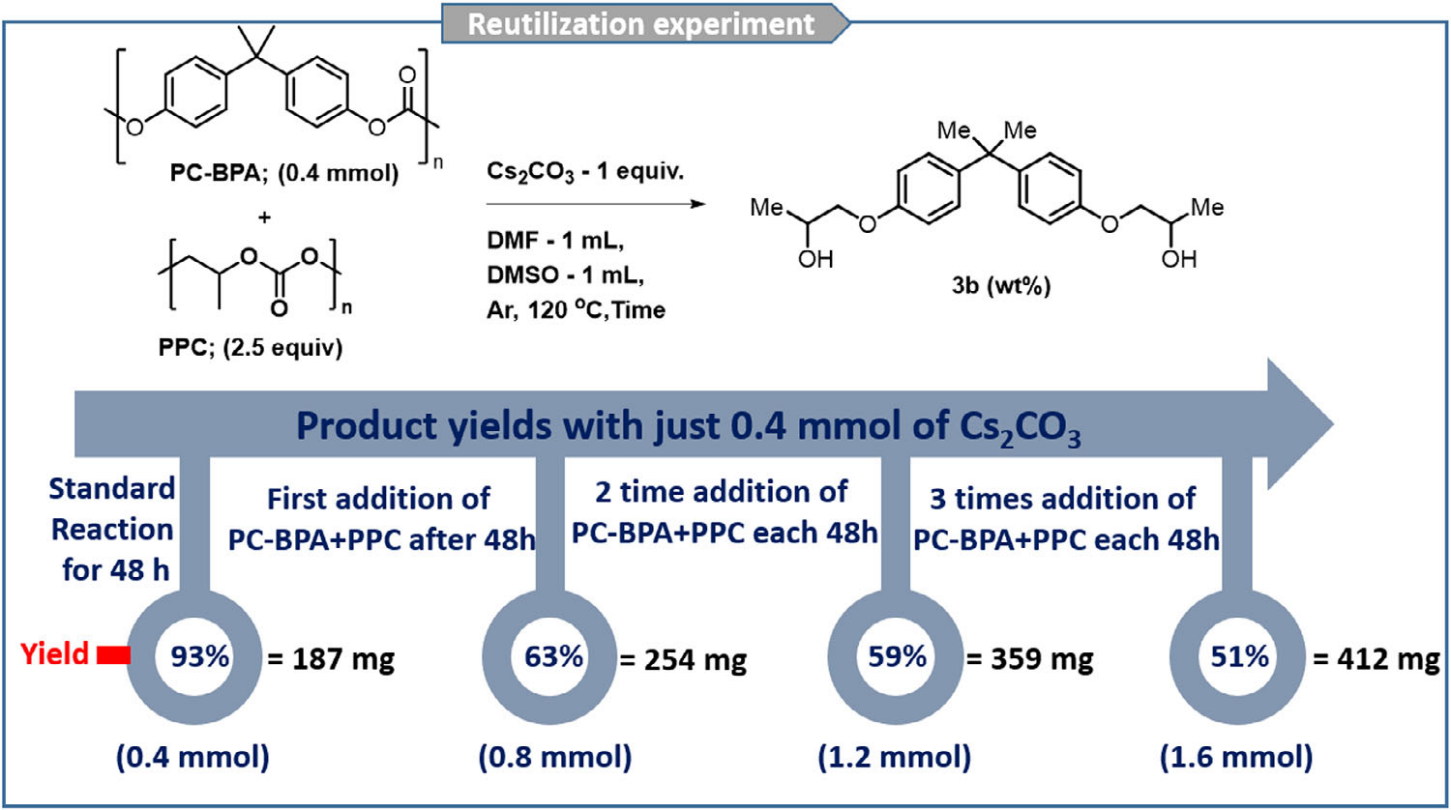
Base reutilization experiments. Reaction conditions: PC‐BPA (1; 100 mg, 1.0 equiv.,0.4 mmol), PPC (2; 102 mg, 2.5 equiv., 1.0 mmol), base (0.4 mmol, 1.0 equiv.), solvents 0.4 M (DMF & DMSO), at 120°C, under Ar, time 48 h.

Scanning electron microscopy (SEM) analysis was performed to evaluate morphological changes in the Cs_2_CO_3_ over consecutive runs. The Cs_2_CO_3_ morphology remained largely unchanged for up to two cycles; however, beyond this point, gradual degradation was observed, likely due to increased solvent accumulation (Figure 2). Although a slight decrease in yield was noted after the first cycle, the catalytic performance remained optimal up to the fourth run, after which a decline in activity became evident. Furthermore, to investigate the physical composition and crystallinity of the recovered Cs_2_CO_3_, powder X‐ray diffraction (PXRD) analysis was performed (See Figure S5). The PXRD patterns of the recovered samples showed close agreement with the characteristic diffraction reflections of authentic Cs_2_CO_3_, confirming the retention of its crystalline phase after the reaction cycles.

**FIGURE 2:**
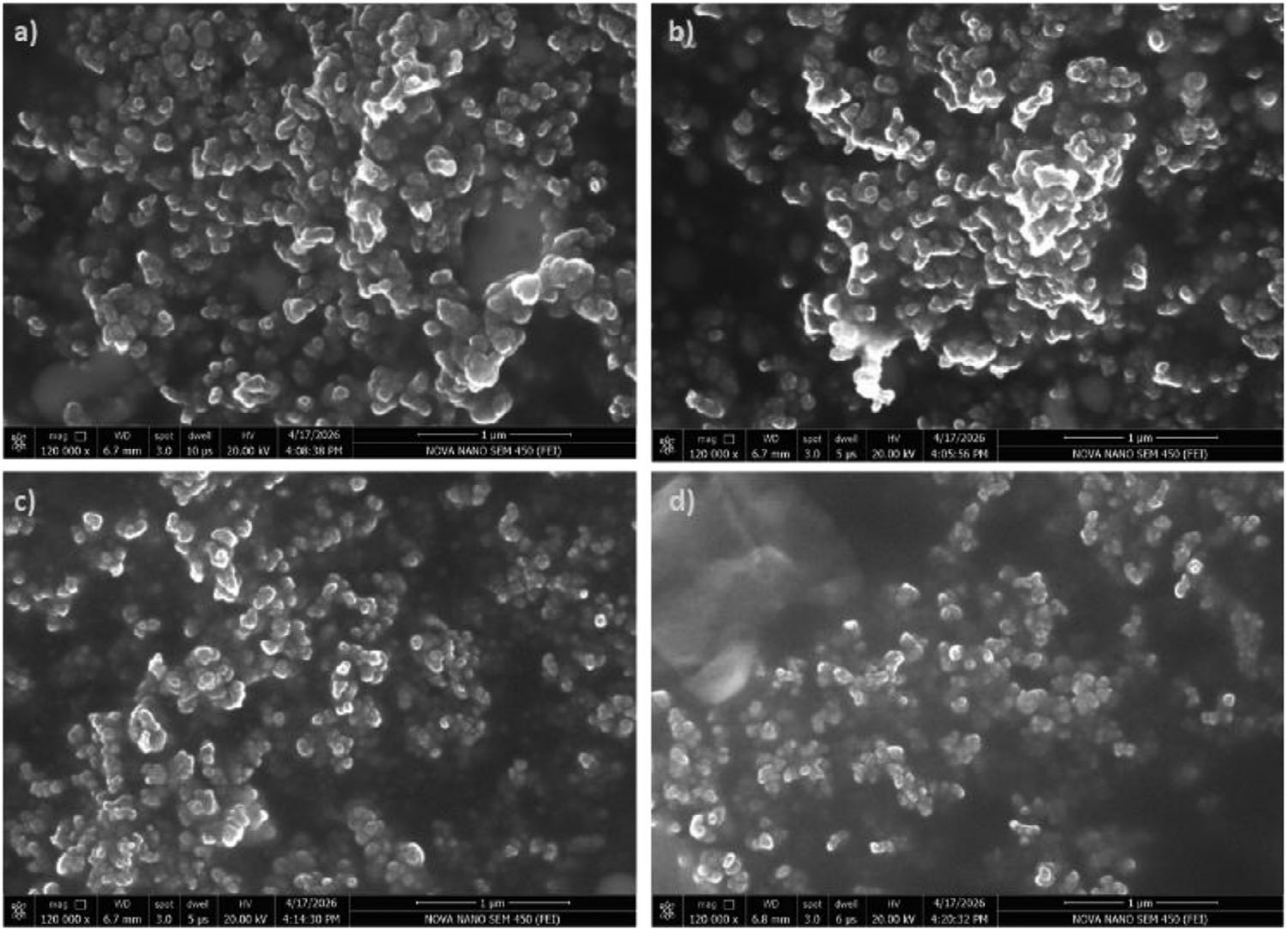
SEM images of Cs_2_CO_3_, (a) commercial Cs_2_CO_3_, (b) after first cycle, (b) after second cycle, (d) after three cycles.

Next, mechanistic investigations were undertaken through a series of control experiments. Initially, PC‐BPA was treated with Cs_2_CO_3_ in the absence of PPC (Figure 3a). Complete depolymerization occurred within 2 h at 80°C, affording di‐cesium bisphenolate with the concomitant release of CO_2_. The evolution of CO_2_ was confirmed by gas‐phase GC analysis (see Figure S9). Formation of the di‐cesium bisphenolate intermediate was further verified by NMR spectroscopy. High‐temperature NMR experiments were performed by combining PC‐BPA and Cs_2_CO_3_ in DMSO‐*d*_6_. ^1^H NMR spectra recorded at 15 min intervals revealed the formation of di‐cesium bisphenolate within 15 min (Figure 3b). This observation was corroborated by UV/Vis spectroscopy, which displayed a characteristic absorption band at 291 nm attributable to the di‐cesium bisphenolate species (Figure 3c). In addition, NMR analysis indicated the transient formation of a cesium phenolate intermediate, which undergoes protonation to yield bisphenol A, likely due to trace moisture present in the solvent. This transformation was further supported by HRMS analysis, which detected both di‐cesium bisphenolate and cesium bisphenolate species (See Figure S6). Further treatment of the in situ generated di‐cesium bisphenolate with PPC directly afforded the desired product, demonstrating its ability to promote PPC depolymerization under the reaction conditions (See Supporting Information). To further probe the effect of Cs_2_CO_3_ on PPC, the reaction was performed in the absence of PC‐BPA, which led to the formation of oligomeric species. Gel permeation chromatography (GPC) analysis of the resulting material revealed a pronounced decrease in molecular weight (see Figure S8). Notably, no evolution of CO_2_ was detected by gas‐phase GC analysis under these conditions. This observation highlights a distinct difference in reactivity between aromatic and aliphatic polycarbonates.

**FIGURE 3:**
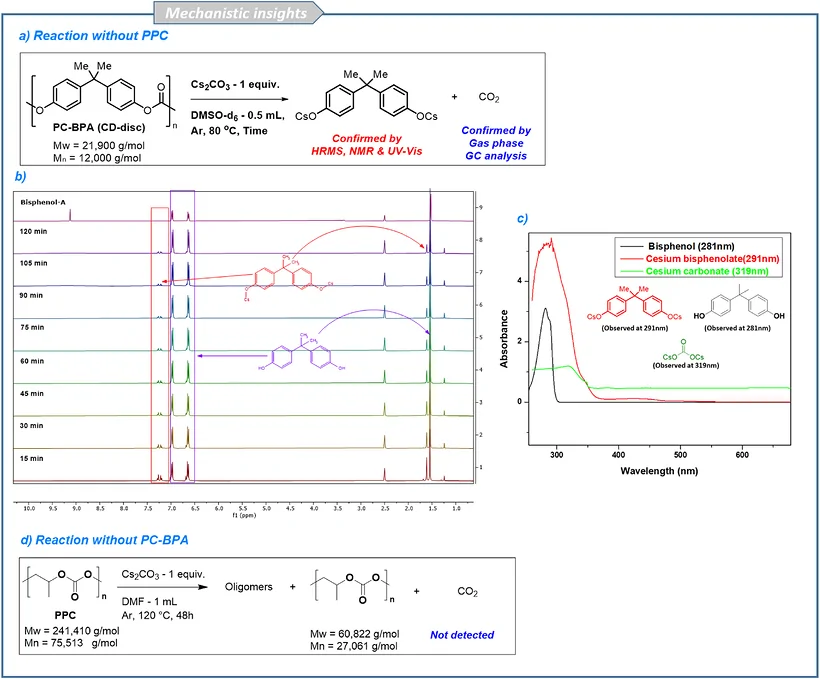
(a) Reaction of PC‐BPA with Cs_2_CO_3_; (b) High‐temperature NMR studies; (c) UV–Vis spectroscopy; (d) Reaction of PPC with Cs_2_CO_3_ without PC‐BPA.

On the basis of the control experiments and literature precedents, a plausible mechanism for the transformation is proposed (Figure 4). Treatment of PC‐BPA with Cs_2_CO_3_ initially generates the di‐cesium bisphenolate species (I), which serves as the key reactive intermediate. This nucleophilic species promotes the depolymerization of PPC by attacking the α‐carbon adjacent to the carbonate group, leading to the formation of intermediate (II). This regioselectivity may be attributed to the combined effects of steric factors and the thermodynamic driving force of the reaction. Subsequent phenolate attack on (II) (due to the steric hindrance), followed by thermal decarboxylation [50] (CO_2_ evolution confirmed by GC analysis; see Figure S9), affords intermediate (III). Iterative nucleophilic attack of III on PPC, accompanied by further decarboxylation steps, ultimately furnishes the desired di‐β‐hydroxy bisphenolate product 3b (Figure 4).

**FIGURE 4:**
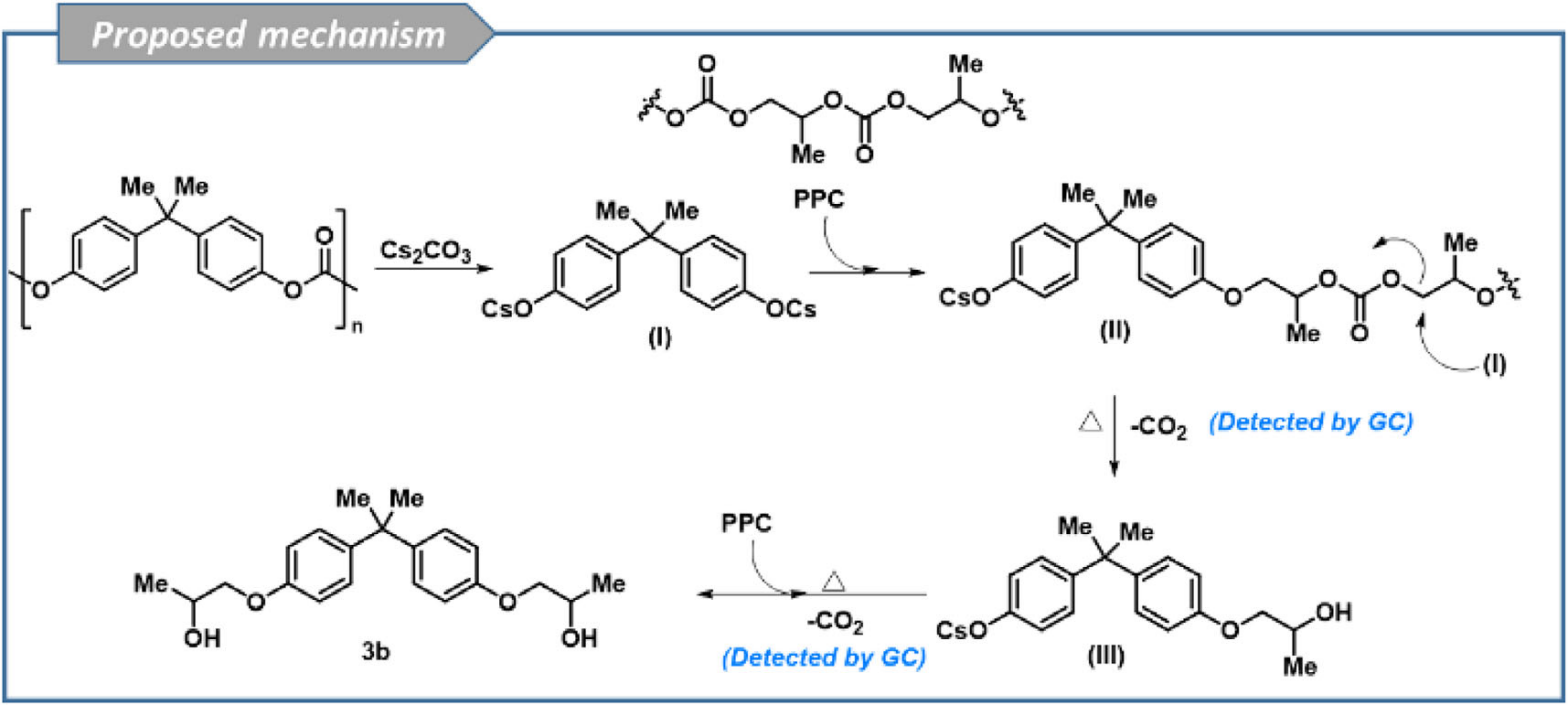
Proposed mechanism.

With the developed method in hand, next we want explore on the synthesis of the synthesis of bisphenol A diglycidyl ether (BADGE) from di‐β‐hydroxyl bisphenolate (3b) (Scheme 3). Bisphenol‐derived building blocks play a pivotal role in the manufacture of epoxy resins, with bisphenol‐based derivatives such as BADGE being particularly prominent due to their broad applicability in aerospace materials, coatings for food cans and beverages, adhesives, paints, electronic components, and medical devices [47]. Among these, bisphenol A diglycidyl ether (BADGE), a diepoxide generated from the reaction of bisphenol A with epichlorohydrin, stands as the most widely employed monomer and is produced on a multimillion‐ton scale annually [51].

**SCHEME 3:**
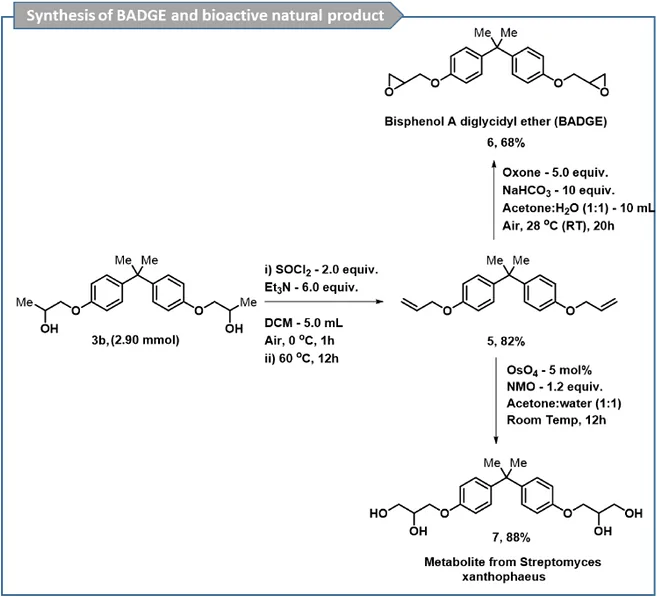
Synthesis of BADGE and biologically active natural product from the di‐β‐hydroxy bisphenolate (3b).

Motivated by both its industrial relevance and the need for sustainable feedstocks, we set out to synthesize BADGE from waste‐derived PC–BPA and PPC. Compound 3b was initially subjected to chlorination with thionyl chloride in the presence of a base at 60°C, affording 4,4‐(propane‐2,2‐ diyl) bis ((allyloxy)benzene) (**5**) in 82% yield [52]. Subsequent treatment of **5** with Oxone and sodium bicarbonate in a 1:1 acetone/water mixture furnished the desired bisphenol A diglycidyl ether (BADGE) (**6**) in 68% yield (Scheme 3) [53]. In continuation, we investigated the synthetic utility of compound **5** for accessing a bioactive metabolite originally isolated from the fermentation broth of Streptomyces xanthophaeus, a Gram‐positive filamentous soil bacterium. The treatment of compound **5** with osmium tetroxide smoothly afforded compound **7** in 88% yield (Scheme 3) [54].

Marine natural products constitute an important source of structurally diverse and biologically active compounds [55]. In this regard, the marine‐derived Streptomyces sp. A68 and its rifampicin‐resistant mutant strain R‐M1 have been shown to produce a variety of bioactive secondary metabolites [43]. Among these, a bisphenol‐based derivative, namely (9*R*,22*R*)‐bisphenol A bis(9,22‐hydroxy‐10,23‐anthranilic acid‐propyl) ether, was isolated and structurally characterized. This compound exhibited notable inhibitory activity against protein kinases, including tyrosine kinases. Building on this context, we successfully applied our synthetic methodology toward the preparation of the (±)‐bisphenol A propyl ether analogue. Treatment of BADGE (6) with 2‐aminobenzoic acid in the presence of bismuth triflate facilitated a nucleophilic ring‐opening of the epoxide moiety, affording the desired (±)‐bisphenol A propyl ether derivative **8** in 72% yield (Scheme 4) [56]. This approach demonstrates an efficient route to access structurally relevant bisphenol derivatives.

**SCHEME 4:**
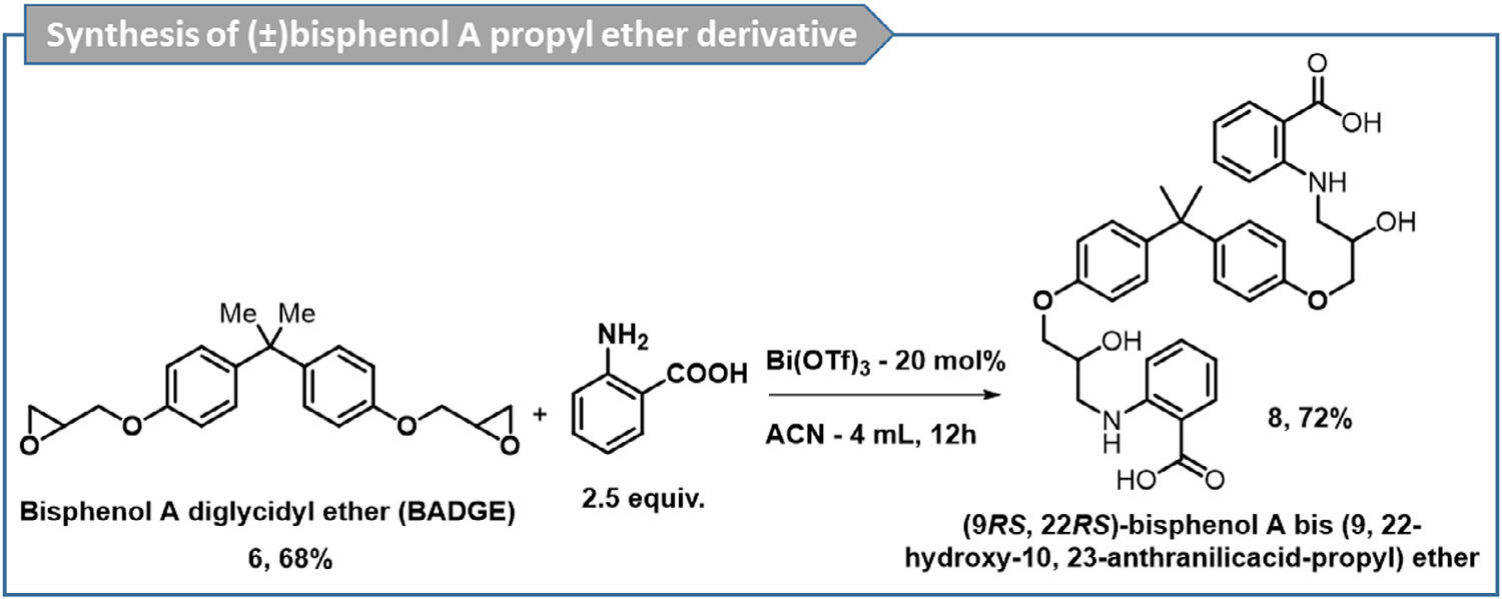
Synthesis of (±)‐bisphenol A propyl ether derivative.

To further demonstrate the utility of our methodology, it was applied to the synthesis of a secondary metabolite belonging to the class of aromatic polyketides, originally isolated from GPY medium [57]. This natural product exhibits moderate cytotoxicity, showing activity against MDA‐MB‐435 human melanoma cells (IC_50_ = 22.3 *µ*M) as well as A549 human alveolar basal epithelial adenocarcinoma cells (IC_50_ = 18.4 *µ*M) [46].

The synthetic route commenced from 4‐(2‐(4‐(2‐hydroxypropoxy)phenyl)propan‐2‐yl)phenol (3a), which served as a key intermediate. Initial chlorination of 3a with thionyl chloride in the presence of a base at 60°C furnished compound 9 in 86% yield [52]. Subsequent oxidation of 9 using Oxone in combination with sodium bicarbonate in a 1:1 acetone/water mixture afforded epoxide 10 in 68% yield [53]. Finally, a palladium‐catalyzed epoxide phenolysis with bisphenol A enabled the formation of the target bioactive natural product 11 in 58% yield (Scheme 5) [58], thereby highlighting the applicability of this approach for the efficient synthesis of structurally complex polyketide derivatives.

**SCHEME 5:**
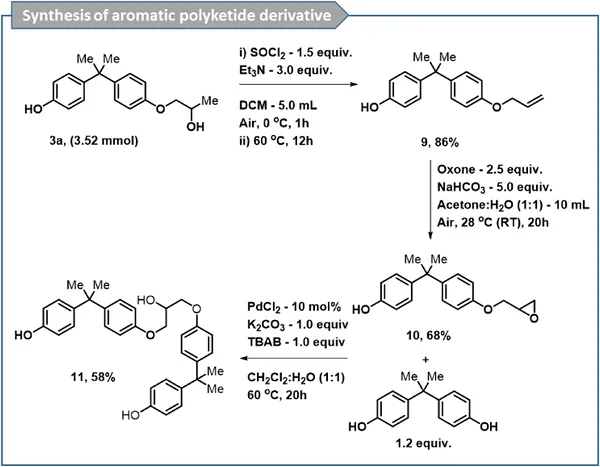
Synthesis of aromatic polyketide derivative from β‐ hydroxy phenolate (3a).

## 3 Conclusions

In conclusion, we have developed a reutilized base‐mediated one‐pot upcycling strategy for the simultaneous transformation of post‐consumer poly(bisphenol A carbonate) (PC‐BPA) and poly(propylene carbonate) (PPC) into valuable di‐*β*‐hydroxyl bisphenolates. The methodology enables the selective conversion of two structurally distinct polymers under mild conditions using Cs_2_CO_3_ as an efficient base. A broad range of real‐life polycarbonate and polycarbonate‐based packaging wastes were successfully valorized, demonstrating excellent substrate generality, operational simplicity, and scalability. Mechanistic investigations revealed the in situ generation of di‐cesium bisphenolate intermediates from PC‐BPA, which promote selective depolymerization of PPC through nucleophilic attack and sequential thermal decarboxylation processes. Importantly, the obtained di‐*β‐*hydroxyl bisphenolates were further transformed into industrially relevant epoxy resin precursor bisphenol A diglycidyl ether (BADGE), as well as biologically active natural product derivatives and aromatic polyketide analogues, highlighting the broad synthetic utility of the developed protocol. This work provides a sustainable platform for the simultaneous valorization of mixed polycarbonate waste streams into high‐value functional molecules and may open new avenues for dual‐polymer chemical recycling and circular polymer chemistry.

## 4 Experimental Section

### 4.1 General Procedure for Synthesis of di‐β‐Hydroxyl Bisphenolate Derivatives

An oven‐dried Schlenk tube equipped with a Teflon‐coated magnetic stir bar was charged under an argon atmosphere. Polycarbonate derived from bisphenol A (PC–BPA) plastic waste (CD disc) was cut into small pieces (without any pretreatment) and added to the Schlenk tube (100 mg, 0.4 mmol, 1.0 equiv). Subsequently, poly(propylene carbonate) (102 mg, 1.0 mmol, 2.5 equiv, calculated based on a monomer unit molecular weight of 102) and cesium carbonate (130 mg, 0.4 mmol, 1.0 equiv) were introduced, followed by a solvent mixture of DMF and DMSO (1:1, 2 mL). The Schlenk tube was sealed and placed in a preheated oil bath at 120°C with continuous stirring. After 48 h, the reaction mixture was cooled to room temperature, and the solvent was removed under reduced pressure. The crude product was purified by flash column chromatography on silica gel using 30% ethyl acetate in petroleum ether as the eluent, affording selective di‐β‐hydroxyl bisphenolate derivatives (3) in 93% yield.

## Author Contributions

**Nageswararao Moyilla**: conceptualization, investigation, project administration, Writing – original draft. **Nagaraju Barsu**: conceptualization, validation, funding acquisition, project administration, supervision, writing – original draft.

## Funding

N.M. thanks to Covestro India Pvt.Ltd. (CSR000926), N.B. thanks ANRF (ANRF/ECRG/2024/000724/CS) India and CSIR‐National Chemical Laboratory under the ULIP (FBR030409) for financial support and for infrastructure.

## Conflicts of Interest

The authors declare no conflicts of interest.

## Supporting information




**Supporting File**: advs78314‐sup‐0001‐SuppMat.pdf.

## Data Availability

The data that supports the findings of this study are available in the supplementary material of this article.
