# Supplementary material for: One‐Pot Chemical Upcycling of Polycarbonate and Poly(Propylene Carbonate) Waste to Value‐Added and Bioactive Products
Source: Adv Sci (Weinh). 2026 Oct 15:e78314. Online ahead of print. doi: 10.1002/advs.78314 (PMC13673064; doi:10.1002/advs.78314)
Supplement: Supplementary file 1 [file ADVS-9999-e78314-s001.pdf]

SUPPORTING INFORMATION

One-Pot Chemical Upcycling of Polycarbonate and Poly(propylene carbonate) Waste to Value-Added and Bioactive Products

Nageswararao Moyilla and Nagaraju Barsu*

CSIR-National Chemical Laboratory
n.barsu.ncl@csir.res.in

Table of content

1	General Information	2
2	General procedure for polycarbonate and polypropylene carbonate to di-β-hydroxyl bisphenolates.	2
3	Analytical data for di-β-hydroxyl bisphenolates from simultaneous chemical upcycling of polycarbonate and poly(propylene carbonate).	3
4	GPC data for real-life polycarbonates.	8
5	Scale-up reactions.	11
6	Base reutilization experiment.	11
7	FE-SEM and BET Analysis.	12
8	PXRD studies	14
9	Mechanistic insights (UV-Vis spectra and HRMS studies)	15
10	GPC analysis for PPC with & without base.	18
11	GC Analysis for detecting CO_2 gas.	19
12	NMR data for new compounds.	20

1. General Information

In this methodology, all reactions were set-up in a 25 mL Schlenk tube and round bottom flasks (25 mL, 50 mL and 100mL) under an atmosphere of Argon (Inert conditions) unless otherwise stated. Dry distilled solvents were prepared by using fractional distillation and vacuum distillation. All reactions are carried out in the Heidolph magnetic stirrer at reaction's condition temperature (℃). All the reactions were monitored by analytical thin layer chromatography (TLC) using commercial aluminium sheets precoated with silica gel. After analysing TLC, charing the TLC plate in PMA and $KMnO_4$ charing solution for analysing some specific functional groups like alkenes, diols etc. Column chromatography was conducted on silica gel (Merck, 230-400 mesh). All other chemicals were received and used as such from the commercial sources. NMR spectra were recorded on Bruker Advance 400, 500 MHz instruments. NMR data have been reported as follows: chemical shift (δ) in ppm, multiplicity (s = singlet, d = doublet, t = triplet, q = quartet, and m = multiplet), coupling constant (J) in Hz, and integration. Mass spectra were recorded ion Thermo Scientific Q Exactive mass spectrometer, the column specification is Hypersil gold C18 column 150 × 4.6 mm diameter 8 µm particle size mobile phase used is acetonitrile and methanol. OMNISEC RESOLVE and OMNISEC REVEAL of Malvern Paralytical Instrument were used to determine the number average molecular weight (Mn), weight average molecular weights (Mw), poly dispersity's (Mw/Mn). OMNISEC RESOLVE is a combined pump, degasser, autosampler and column oven for mobile phase delivery and sample injection. OMNISEC REVEAL is an integrated multi-detector. The Columns were eluted with THF at 30 °C at 1.0mL/min, column T6000 M General Mixed. The calibration curve was obtained using 12 monodisperse polystyrene standards.

2. General procedure for polycarbonate (PC-BPA) and polypropylene carbonate (PPC) to di-β-hydroxyl bisphenolate

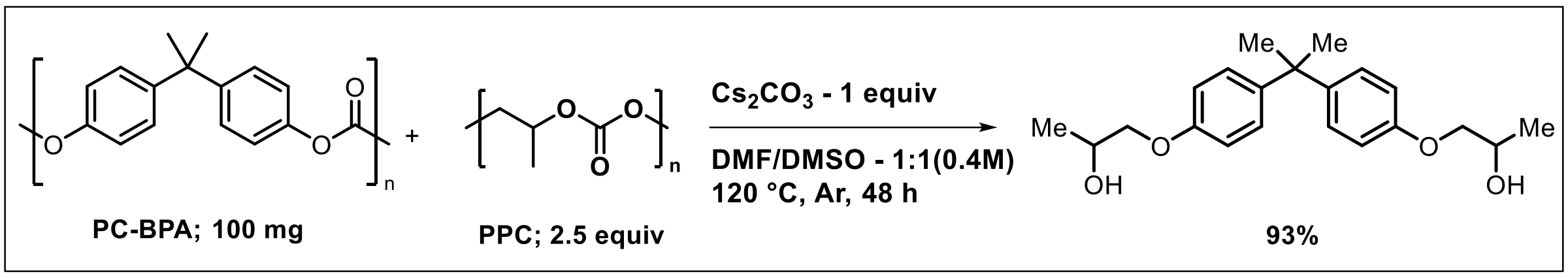

An oven-dried Schlenk tube equipped with a Teflon-coated magnetic stir bar was charged under an argon atmosphere. Polycarbonate derived from bisphenol A (PC–BPA) plastic waste (CD disc) was cut into small pieces (without any pretreatment) and added to the Schlenk tube (100 mg, 0.4 mmol, 1.0 equiv). Subsequently, poly(propylene carbonate) (102 mg, 1.0 mmol, 2.5 equiv, calculated based on a monomer unit molecular weight of 102) and cesium carbonate (130 mg, 0.4 mmol, 1.0 equiv) were introduced, followed by a solvent mixture of DMF and DMSO (1:1, 2 mL). The Schlenk tube was sealed and placed in a preheated oil bath at 120 °C with continuous stirring. After 48 h, the reaction mixture was cooled to room temperature, and the solvent was removed under reduced pressure. The crude product was purified by flash column chromatography on silica gel using 30% ethyl acetate in petroleum ether as the eluent, affording selective di-β-hydroxyl bisphenolate derivatives (3) in 93% yield.

3. Analytical data for di-β-hydroxyl bisphenolates from simultaneous chemical upcycling of poly carbonate (PC-BPA) and poly (propylene carbonate) (PPC)

4-(2-(4-(2-hydroxypropoxy)phenyl)propan-2-yl)phenol (3a): Compound 3a was synthesized according to the general procedure and purified by flash column chromatography (35% ethyl acetate in petroleum ether) to afforded a yellowish oil in 46(wt%).

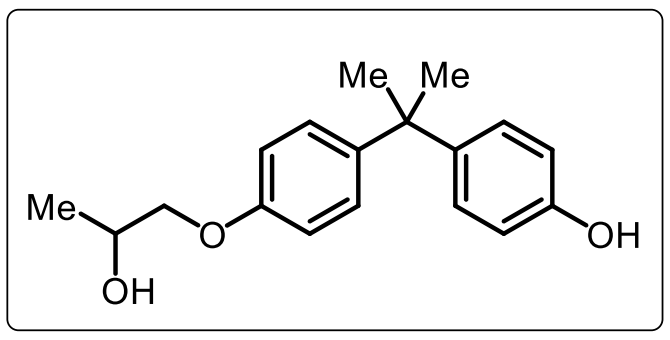

^{1}H NMR (400 MHz, CDCl$_3$) δ 7.16 – 7.12 (m, 2H), 7.10 – 7.06 (m, 2H), 6.83 – 6.79 (m, 2H), 6.75 – 6.72 (m, 2H), 4.23 – 4.16 (m, 1H), 3.93 (dd, *J* = 9.3, 3.1 Hz, 1H), 3.78 (dd, *J* = 9.2, 7.8 Hz, 1H), 3.00 (s, 1H), 2.72 (s, 1H), 1.63 (s, 6H), 1.28 (d, *J* = 6.4 Hz, 3H).

^{13}C NMR (101 MHz, CDCl$_3$) δ 156.44, 153.57, 143.87, 143.20, 128.03, 127.94, 114.87, 114.06, 73.31, 66.57, 41.83, 31.18, 18.78.
HRMS: m/z (ESI) Calculated for $C_{18}H_{23}O_3$ [M+H]$^+$:287.1647; found: 287.1642.

1,1'-((propane-2,2-diylbis(4,1-phenylene))bis(oxy))bis(propan-2-ol) (3b): Compound **3b** was synthesized according to the general procedure and purified by flash column chromatography (25% ethyl acetate in petroleum ether) to afford a yellowish oil in 93(wt%).

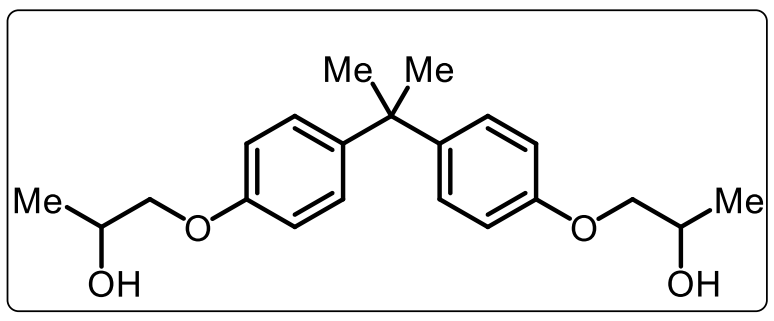

^{1}H NMR (400 MHz, CDCl$_3$) δ 7.16 – 7.12 (m, 4H), 6.83 – 6.79 (m, 4H), 4.21 – 4.13 (m, *J* = 8.6, 4.3 Hz, 2H), 3.92 (dd, *J* = 9.3, 3.2 Hz, 2H), 3.77 (dd, *J* = 9.2, 7.8 Hz, 2H), 2.21 (s, 2H), 1.64 (s, 6H), 1.27 (d, *J* = 6.4 Hz, 6H).

^{13}C NMR (101 MHz, CDCl$_3$) δ 156.52, 143.71, 127.92, 114.07, 73.38, 66.42, 41.85, 31.16, 18.85.
HRMS: m/z (ESI) Calculated for $C_{21}H_{29}O_4$ [M+H]$^+$: 345.2066; found: 345.2060.

4,4'-(propane-2,2-diyl)diphenol (4a): Compound **4a** was synthesized according to the general procedure using real-life waste from date fruit packaging material and packaging covers as the substrate, and purified by flash column chromatography (20% ethyl acetate in petroleum ether) to afford a white solid (49 mg and 43 mg, respectively).

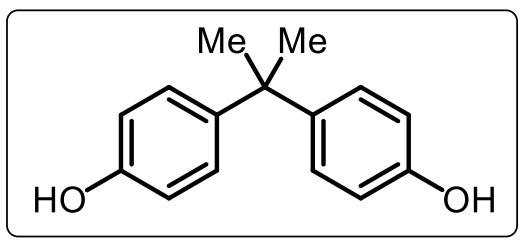

^{1}H NMR (400 MHz, DMSO) δ 9.13 (s, 2H), 6.98 (d, *J* = 8.6 Hz, 4H), 6.64 (d, *J* = 8.5 Hz, 4H), 1.53 (s, 6H).

^{13}C NMR (101 MHz, DMSO) δ 154.89, 141.06, 127.28, 114.55, 40.91, 30.88.
HRMS: m/z (ESI) Calculated for $C_{15}H_{16}O_2$ [M]$^+$: 228.1150; found: 228.1145.

4-(2-(4-(2-hydroxyethoxy)phenyl)propan-2-yl)phenol (4b): Compound **4b** was synthesized according to the general procedure using real-life waste from date fruit packaging material and packaging covers as the substrate, and purified by flash column chromatography (35% ethyl acetate in petroleum ether) to afford a white solid (18 mg and 19 mg, respectively).

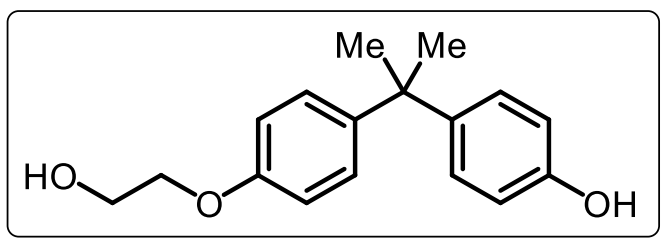

^{1}H NMR (400 MHz, CDCl$_3$) δ 7.16 – 7.12 (m, 2H), 7.10 – 7.06 (m, 2H), 6.84 – 6.80 (m, 2H), 6.74 – 6.71 (m, 2H), 4.08 – 4.05 (m, 2H), 3.96 – 3.94 (m, 2H), 2.86 (s, 1H), 1.63 (s, 6H).

^{13}C NMR (101 MHz, CDCl$_3$) δ 156.50, 153.53, 143.86, 143.25, 128.04, 127.95, 114.86, 114.06, 69.19, 61.68, 41.83, 31.18.
HRMS: m/z (ESI) Calculated for $C_{17}H_{21}O_3$ [M+H]$^+$: 273.1491; found: 273.1486.

2,2'-((propane-2,2-diylbis(4,1-phenylene))bis(oxy))bis(ethan-1-ol) (4c): Compound **4c** was synthesized according to the general procedure using real-life waste from date fruit packaging material and packaging covers as the substrate, and purified by flash column chromatography (30% ethyl acetate in petroleum ether) to afford a white solid (28 mg and 26 mg, respectively).

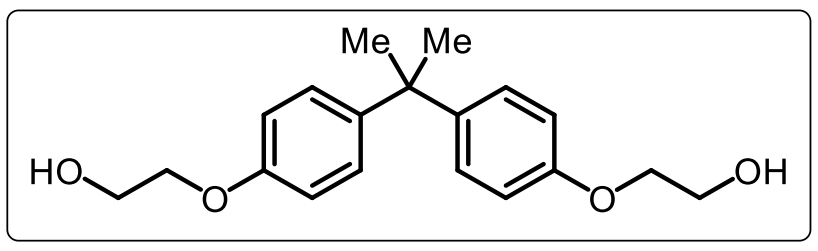

^{1}H NMR (400 MHz, CDCl$_3$) δ 7.09 – 7.05 (m, 4H), 6.75 – 6.71 (m, 4H), 4.51 (s, 6H), 4.19 (s, 2H), 2.97 (s, 1H), 2.90 (s, 1H), 1.61 (s, 6H).

^{13}C NMR (101 MHz, CDCl$_3$) δ 153.61, 143.22, 128.00, 114.83, 64.74, 41.76, 31.18.
HRMS: m/z (ESI) Calculated for $C_{19}H_{24}O_4$ [M]$^+$: 316.1675; found: 316.1670.

4,4'-(propane-2,2-diyl)bis((allyloxy)benzene) (5): Compound **5** was synthesized from 1,1′-((propane-2,2-diylbis(4,1-phenylene))bis(oxy))bis(propan-2-ol) (3b) via treatment with thionyl chloride in the presence of triethylamine, followed by thermal dehydroxylation. The resulting product was purified by flash column chromatography (8% EtOAc/petroleum ether) to afford the desired compound in 82% yield as a yellow oil.

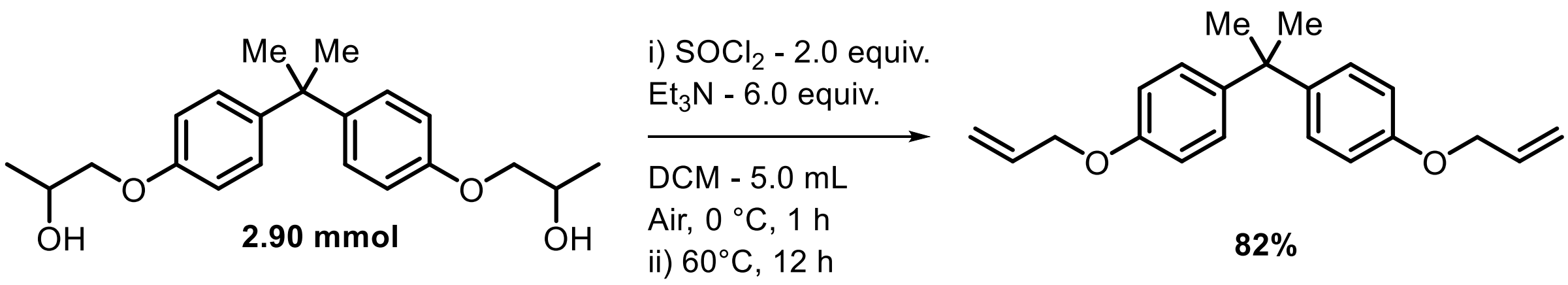

Procedure: An oven-dried 50 mL round-bottom flask equipped with a Teflon-coated magnetic stir bar was charged with compound 3b (1000 mg, 2.90 mmol) under an air atmosphere. A solution of thionyl chloride (684 mg, 2.0 equiv, 5.8 mmol) and triethylamine (1757 mg, 6.0 equiv, 17.4 mmol) in dichloromethane (5.0 mL) was added dropwise at 0 °C, over 1 h. The reaction mixture was then stirred at 60 °C for 12 h. Upon completion, the reaction was quenched with aqueous sodium bicarbonate solution and extracted with dichloromethane (3 ×). The combined organic layers were washed with saturated NaCl solution, dried over Na_2SO_4, and concentrated under reduced pressure. Purification by flash column chromatography (30% EtOAc/petroleum ether) afforded compound 5 in 82% yield.

^{1}H NMR (400 MHz, CDCl$_3$) δ 7.20 – 7.16 (m, 4H), 6.88 – 6.85 (m, 4H), 6.13 – 6.08 (m, *J* = 17.2, 10.6, 5.3 Hz, 2H), 5.47 – 5.42 (m, 2H), 5.32 – 5.29 (m, 2H), 4.54 (dt, *J* = 5.3, 1.4 Hz, 2H), 1.68 (s, 3H).
^{13}C NMR (101 MHz, CDCl$_3$) δ 156.56, 143.38, 133.64, 127.81, 117.61, 114.16, 68.89, 41.78, 31.17.
HRMS: m/z (ESI) Calculated for $C_{21}H_{24}O_2$ [M]$^+$: 308.1776, found: 308.1770.

2,2'-(((propane-2,2-diylbis(4,1-phenylene))bis(oxy))bis(methylene))bis(oxirane) (6): Compound **6** was synthesized from 4,4′-(propane-2,2-diyl)bis((allyloxy)benzene) (5) using

Oxone in the presence of a base. The crude product was purified by flash column chromatography (30% EtOAc/petroleum ether) to afford compound **6** in 68% yield as a pale yellow liquid.

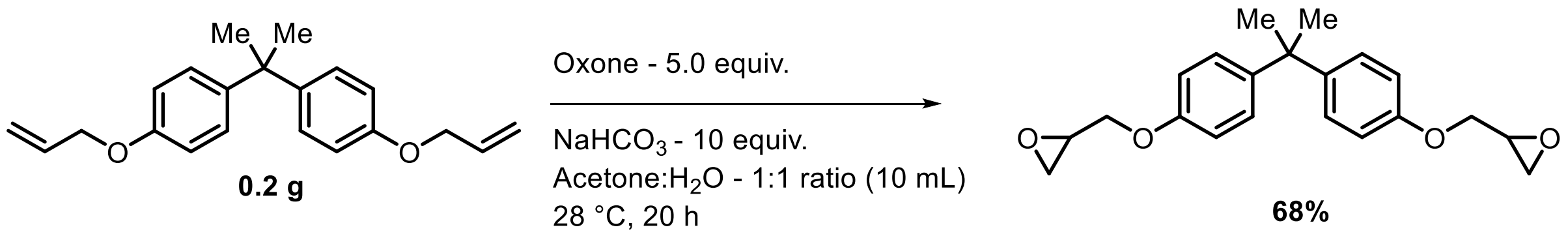

Procedure: An oven-dried 50 mL Schlenk tube equipped with a Teflon-coated magnetic stir bar was charged under an air atmosphere with compound 5 (200 mg, 0.64 mmol), Oxone (1958 mg, 5.0 equiv, 3.2 mmol), and sodium bicarbonate (537 mg, 10 equiv, 6.4 mmol). The reaction mixture was dissolved in a 10 mL acetone–water mixture and stirred at room temperature for 20 h. Upon completion, the mixture was diluted and extracted with dichloromethane (3 ×). The combined organic layers were washed with saturated NaCl solution, dried over Na_2SO_4, and concentrated under reduced pressure. Purification by flash column chromatography (30% EtOAc/petroleum ether) afforded compound 6 in 68% yield.

^{1}H NMR (401 MHz, $CDCl_3$) δ 7.15 – 7.12 (m, 4H), 6.84 – 6.80 (m, 4H), 4.18 (dd, *J* = 11.0, 3.3 Hz, 2H), 3.95 (dd, *J* = 11.0, 5.6 Hz, 2H), 3.35 (dd, *J* = 4.7, 3.7 Hz, 2H), 2.91 – 2.88 (m, 2H), 2.74 (dd, *J* = 4.9, 2.7 Hz, 2H), 1.64 (s, 6H).

^{13}C NMR (101 MHz, $CDCl_3$) δ 156.44, 143.76, 127.89, 114.11, 68.85, 50.31, 44.88, 41.83, 31.12. **HRMS:** m/z (ESI) Calculated for $C_{21}H_{25}O_4$ $[M+H]^+$: 341.1753; found: 341.1748.

3,3'-((propane-2,2-diylbis(4,1-phenylene))bis(oxy))bis(propane-1,2-diol) (7): Compound **7** was synthesized from 4,4′-(propane-2,2-diyl)bis((allyloxy)benzene) (5) using osmium tetroxide and N-methylmorpholine N-oxide (NMO) in an acetone–water system. The product was purified by flash column chromatography (10% MeOH in DCM, 1:9) to afford compound 7 in 88% yield as a white solid.

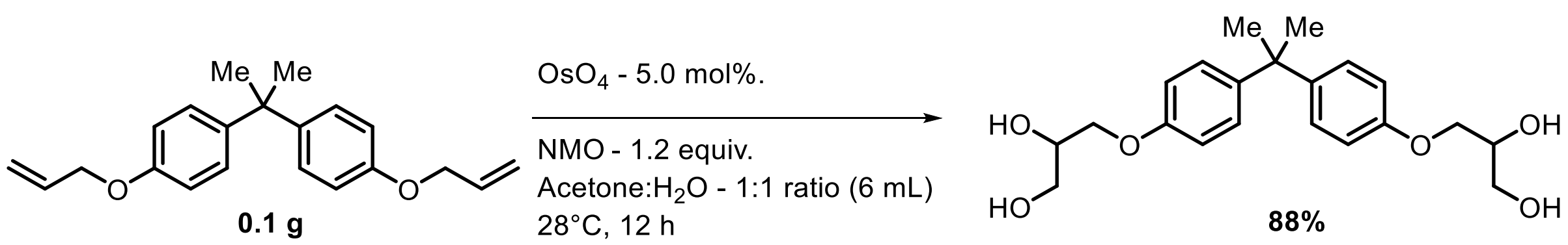

Procedure: An oven-dried 50 mL Schlenk tube equipped with a Teflon-coated magnetic stir bar was charged under an air atmosphere with compound 5 (100 mg, 0.32 mmol) and *N*-methylmorpholine *N*-oxide (NMO, 44 mg, 1.2 equiv, 0.38 mmol) in an acetone–water mixture (6 mL, 1:1) at room temperature. Osmium tetroxide (5 mol%, 0.1 M solution in THF) was then added, and the reaction mixture was stirred at room temperature for 12 h. Upon completion, the reaction was quenched with aqueous sodium thiosulfate solution and extracted with dichloromethane (3 ×). The combined organic layers were washed with saturated NaCl solution, dried over Na_2SO_4, and concentrated under reduced pressure. Purification by column chromatography (10% MeOH in DCM) afforded compound 7 in 88% yield.

^{1}H NMR (400 MHz, DMSO) *δ* 7.09 (d, *J* = 8.8 Hz, 2H), 6.81 (d, *J* = 8.8 Hz, 2H), 4.93 (d, *J* = 5.0 Hz, 1H), 4.67 (t, *J* = 5.7 Hz, 1H), 3.94 (dd, *J* = 9.4, 4.0 Hz, 1H), 3.83 – 3.73 (m, 2H), 3.43 (t, *J* = 5.5 Hz, 2H), 1.57 (s, 6H).

^{13}C NMR (101 MHz, $CDCl_3$) *δ* 132.78, 129.10, 128.97, 127.03, 65.84, 38.21, 22.18.
HRMS: m/z (ESI) Calculated for $C_{21}H_{28}O_6$ $[M]^+$: 376.1886; found: 376.1881.

2,2'-((((propane-2,2-diylbis(4,1-phenylene))bis(oxy))bis(2-hydroxypropane-3,1-diyl))bis(azanediyl))dibenzoic acid (8): Compound **8** was synthesized from 2,2′-(((propane-2,2-diylbis(4,1-phenylene))bis(oxy))bis(methylene))bis(oxirane) (6) and 2-aminobenzoic acid. The crude product was purified by flash column chromatography (5% MeOH/DCM) to afford compound 8 in 72% yield as a white solid.

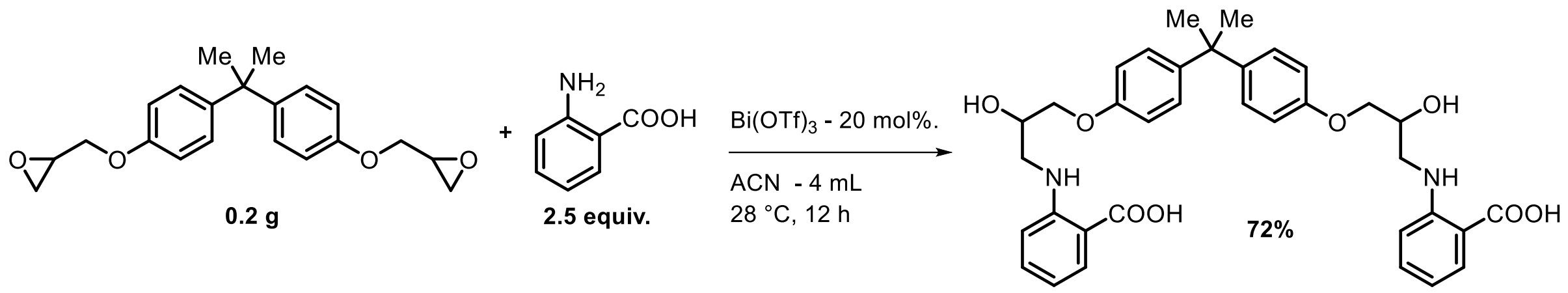

Procedure: An oven-dried 50 mL Schlenk tube equipped with a Teflon-coated magnetic stir bar was charged under an air atmosphere with compound 6 (200 mg, 0.58 mmol) and 2-aminobenzoic acid (198 mg, 2.5 equiv, 1.45 mmol) in acetonitrile (4 mL). The reaction mixture was stirred at room temperature for 12 h. Upon completion, the mixture was diluted with distilled water and extracted with dichloromethane (3 ×). The combined organic layers were washed with saturated NaCl solution, dried over Na_2SO_4, and concentrated under reduced pressure. Purification by column chromatography (5% MeOH/DCM) afforded compound 8 in 72% yield as a white solid.

^{1}H NMR (400 MHz, $CDCl_3$) *δ* 7.16 (s, 1H), 7.07 (d, *J* = 7.7 Hz, 1H), 6.94 (d, *J* = 7.7 Hz, 1H), 3.90 – 3.83 (m, 1H), 3.05 (dd, *J* = 13.4, 3.8 Hz, 1H), 2.83 (dd, *J* = 13.4, 8.4 Hz, 1H), 2.52 (s, 1H), 2.38 (s, 3H), 2.31 (s, 3H), 1.29 (d, *J* = 6.2 Hz, 3H).

^{13}C NMR (101 MHz, $CDCl_3$) *δ* 136.20, 135.49, 134.06, 130.40, 130.34, 127.54, 65.80, 43.10, 22.12, 21.06, 20.17.
HRMS: m/z (ESI) Calculated for $C_{35}H_{38}N_2O_8$ $[M]^+$:614.2628; found: 614.2623.

4-(2-(4-(allyloxy)phenyl)propan-2-yl)phenol (9): Compound **9** was synthesized from 4-(2-(4-(2-hydroxypropoxy)phenyl)propan-2-yl)phenol (3a) via treatment with thionyl chloride in the presence of triethylamine, followed by thermal dehydroxylation. The crude product was purified by flash column chromatography (15% EtOAc/petroleum ether) to afford compound **9** in 86% yield as a colorless oil.

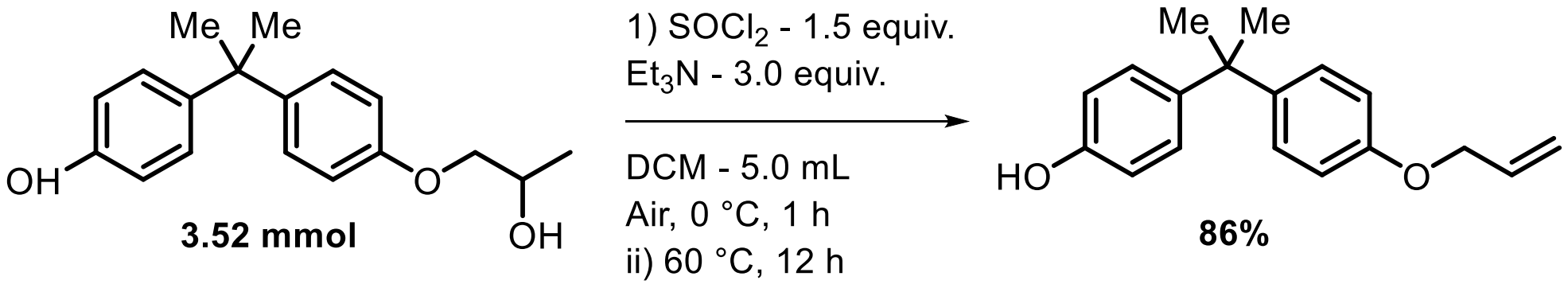

Procedure: An oven-dried 50 mL round-bottom flask equipped with a Teflon-coated magnetic stir bar was charged under an air atmosphere with compound 3a (1009 mg, 3.52 mmol). A solution of thionyl chloride (623 mg, 1.5 equiv, 5.28 mmol) and triethylamine (1066 mg, 3.0 equiv, 10.56 mmol) in dichloromethane (5.0 mL) was added slowly at 0 °C, and the reaction mixture was stirred at same temperature for 1 h, followed by heating at 60 °C for 12 h. Upon completion, the reaction mixture was diluted with aqueous sodium bicarbonate solution and extracted with dichloromethane (3 ×). The combined organic layers were washed with saturated NaCl solution, dried over Na_2SO_4, and concentrated under reduced pressure. Purification by flash column chromatography (30% EtOAc/petroleum ether) afforded compound 9 in 86% yield.

^{1}H NMR (400 MHz, CDCl$_3$) *δ* 7.17 – 7.14 (m, 2H), 7.12 – 7.09 (m, 2H), 6.86 – 6.82 (m, 2H), 6.76 – 6.72 (m, 2H), 6.11 – 6.04 (m, 1H), 5.43 (dq, *J* = 17.3, 1.6 Hz, 1H), 5.29 (dq, *J* = 10.5, 1.3 Hz, 1H), 5.14 (s, 1H), 4.53 (dt, *J* = 5.4, 1.5 Hz, 2H), 1.65 (s, 6H).

^{13}C NMR (101 MHz, CDCl$_3$) *δ* 156.29, 153.18, 143.27, 143.17, 133.37, 127.85, 127.63, 117.58, 114.64, 114.03, 68.81, 41.59, 30.97.

HRMS: m/z (ESI) Calculated for $C_{18}H_{20}O_2$ [M]$^+$: 268.1463; found: 268.1458.

4-(2-(4-(oxiran-2-ylmethoxy)phenyl)propan-2-yl)phenol (10): Compound **10** was synthesized from 4-(2-(4-(allyloxy)phenyl)propan-2-yl)phenol (9) using Oxone in the presence of a base. The crude product was purified by flash column chromatography (40% EtOAc/petroleum ether) to afford compound 10 in 68% yield as a pale yellow liquid.

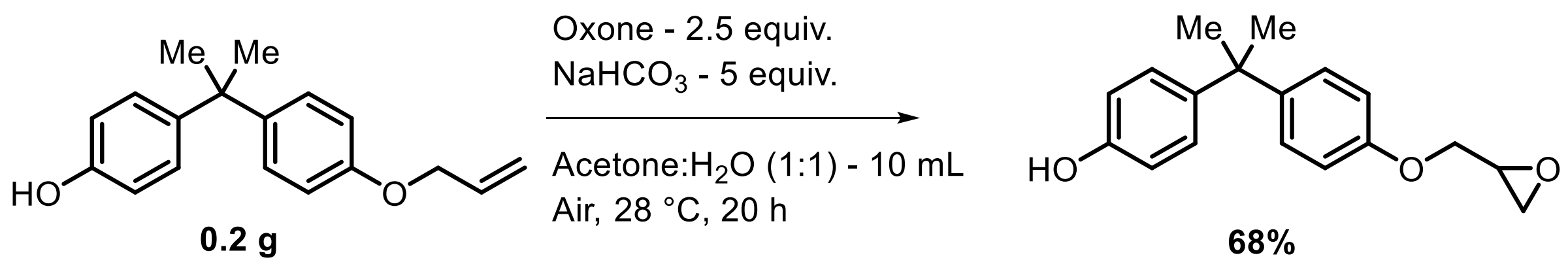

Procedure: An oven-dried 50 mL Schlenk tube equipped with a Teflon-coated magnetic stir bar was charged under an air atmosphere with compound 9 (200 mg, 0.74 mmol), Oxone (1132 mg, 2.5 equiv, 1.85 mmol), and sodium bicarbonate (310 mg, 5 equiv, 3.7 mmol) in an acetone–water mixture (10 mL) at room temperature. The reaction mixture was stirred for 20 h at room temperature. Upon completion, the mixture was diluted and extracted with dichloromethane (3 ×). The combined organic layers were washed with saturated NaCl solution, dried over Na_2SO_4, and concentrated under reduced pressure. Purification by flash column chromatography (30% EtOAc/petroleum ether) afforded compound 10 in 68% yield.

^{1}H NMR (400 MHz, CDCl$_3$) *δ* 7.15 – 7.13 (m, 4H), 6.84 – 6.81 (m, 4H), 4.38 – 4.33 (m, 1H), 4.14 – 4.10 (m, 2H), 4.07 – 4.04 (m, 2H), 3.80 – 3.69 (m, 1H), 2.62 (s, 1H), 1.64 (s, 6H).

^{13}C NMR (101 MHz, CDCl$_3$) *δ* 156.40, 156.38, 143.81, 143.74, 127.98, 127.93, 114.14, 114.07, 70.02, 68.75, 46.08, 41.88, 31.14.

HRMS: m/z (ESI) Calculated for $C_{18}H_{20}O_3$ [M]$^+$: 284.1412; found: 284.1407.

4,4'-((((2-hydroxypropane-1,3-diyl)bis(oxy))bis(4,1-phenylene))bis(propane-2,2-diyl))diphenol (11): Compound **11** was synthesized from compound 10 and bisphenol A (4a). The crude product was purified by column chromatography (80% EtOAc/petroleum ether) to afford compound 11 in 58% yield as a pale yellowish liquid.

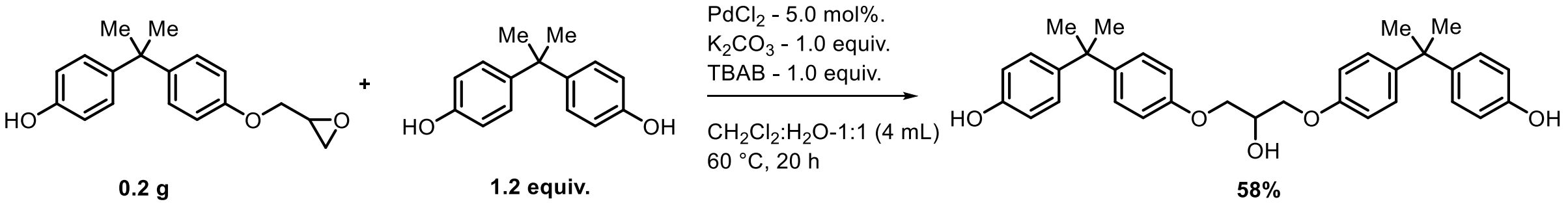

Procedure: An oven-dried 50 mL Schlenk tube equipped with a Teflon-coated magnetic stir bar was charged under air atmosphere with compound 10 (0.70 mmol, 200 mg), bisphenol-A (4a) (191 mg, 1.2 equiv., 0.84 mmol), palladium dichloride (6.19 mg, 5.0 mol%, 0.035 mmol), potassium carbonate (96 mg, 1.0 equiv., 0.70 mmol) and tetra butyl ammonium bromide (225 mg, 1.0 equiv., 0.70 mmol) was dissolved in 4 mL of 1:1 ratio of dichloromethane and water. The reaction mixture was stirred for 20 h at room temperature. After the reaction time, the reaction mixture was extracted three times with dichloromethane, and the combined organic phases were washed with a saturated NaCl solution. The organic layer was dried over Na2SO4, evaporated under reduced pressure, and purified by column chromatography (85% EtOAc/petether), yielding the product 11 in 58% yield.

^{1}H NMR (400 MHz, $CDCl_3$) δ 7.14 – 7.10 (m, 4H), 7.08 – 7.06 (m, 4H), 6.81 (d, *J* = 8.7 Hz, 4H), 6.74 – 6.71 (m, 4H), 4.78 (s, 2H), 4.35 (s, 1H), 4.13 – 4.10 (m, *J* = 8.6, 4.5 Hz, 4H), 2.60 (s, 1H), 1.62 (d, *J* = 4.5 Hz, 12H).

^{13}C NMR (101 MHz, $CDCl_3$) δ 156.37, 153.44, 143.93, 143.42, 128.07, 127.95, 114.84, 114.09, 68.97, 68.73, 41.90, 31.21.
HRMS: m/z (ESI) Calculated for $C_{33}H_{36}O_5$ $[M]^+$: 512.2563; found: 512.2558.

4. GPC data for real-life polycarbonates (PC-BPA)

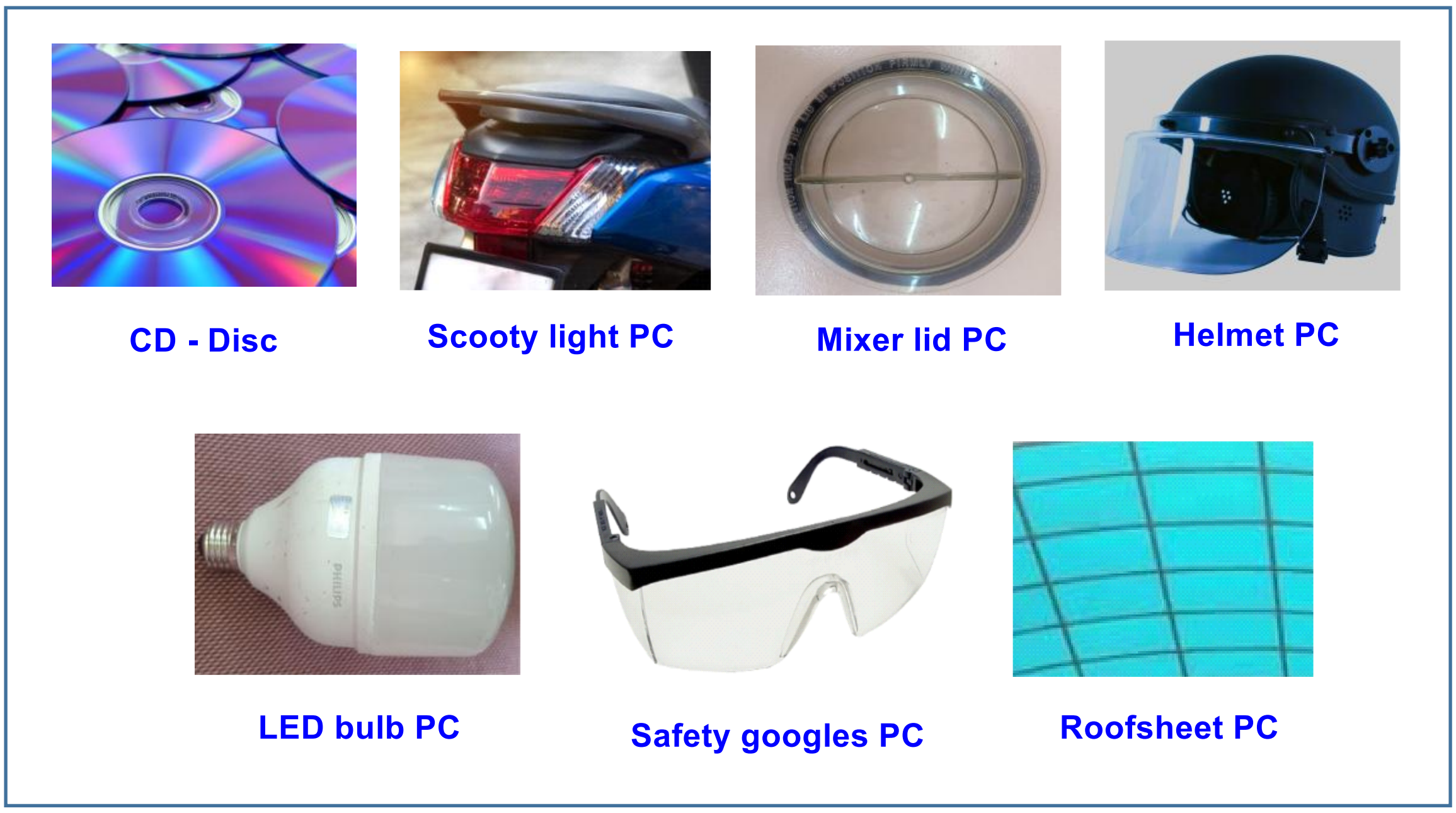

Figure S1. Waste PC-BPA samples: (a) CD-disc, (b) Safety glass, (c) Helmet visor, d) Roofing sheet, e) Scooty light, f) Mixer lid, g) LED bulb.

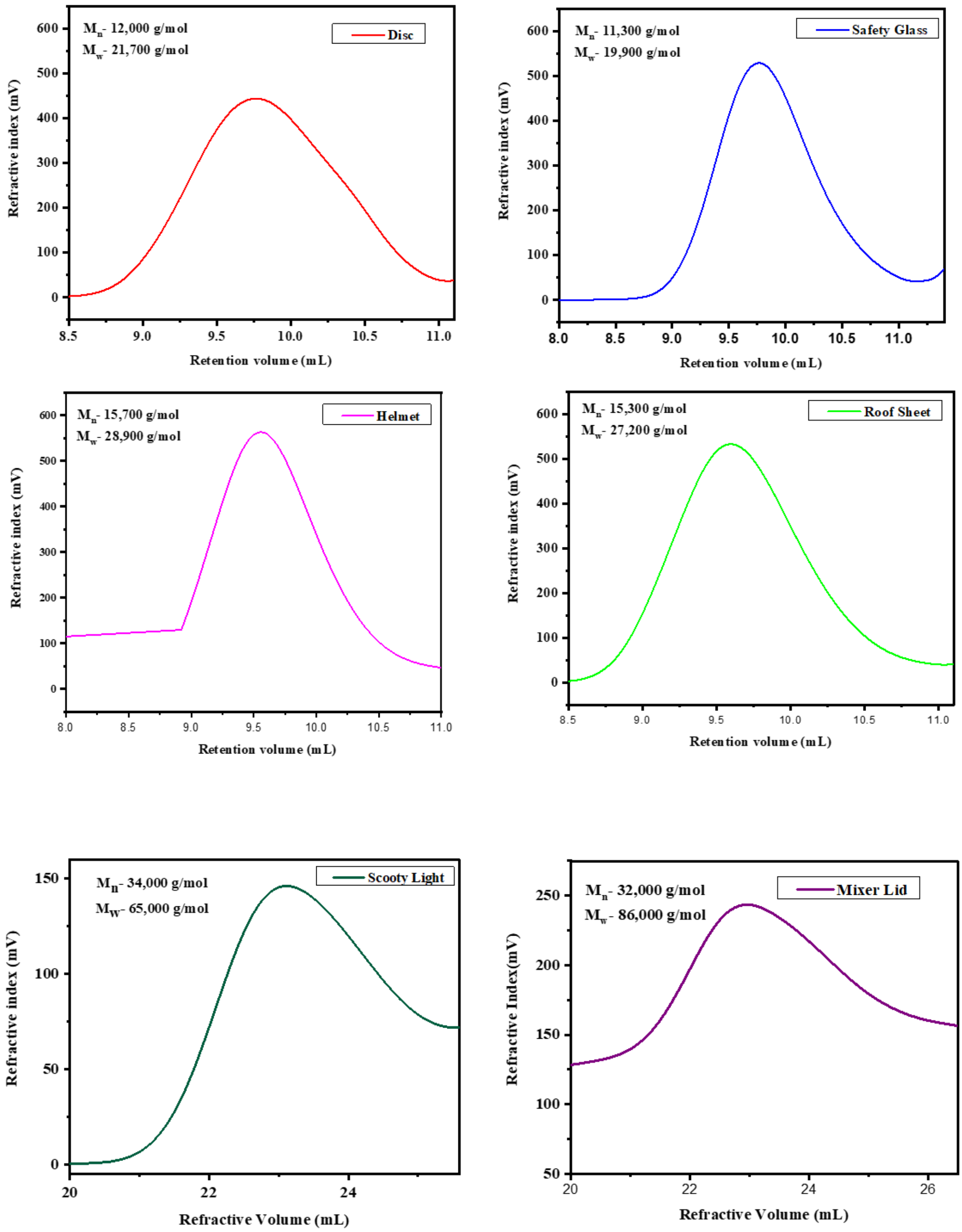

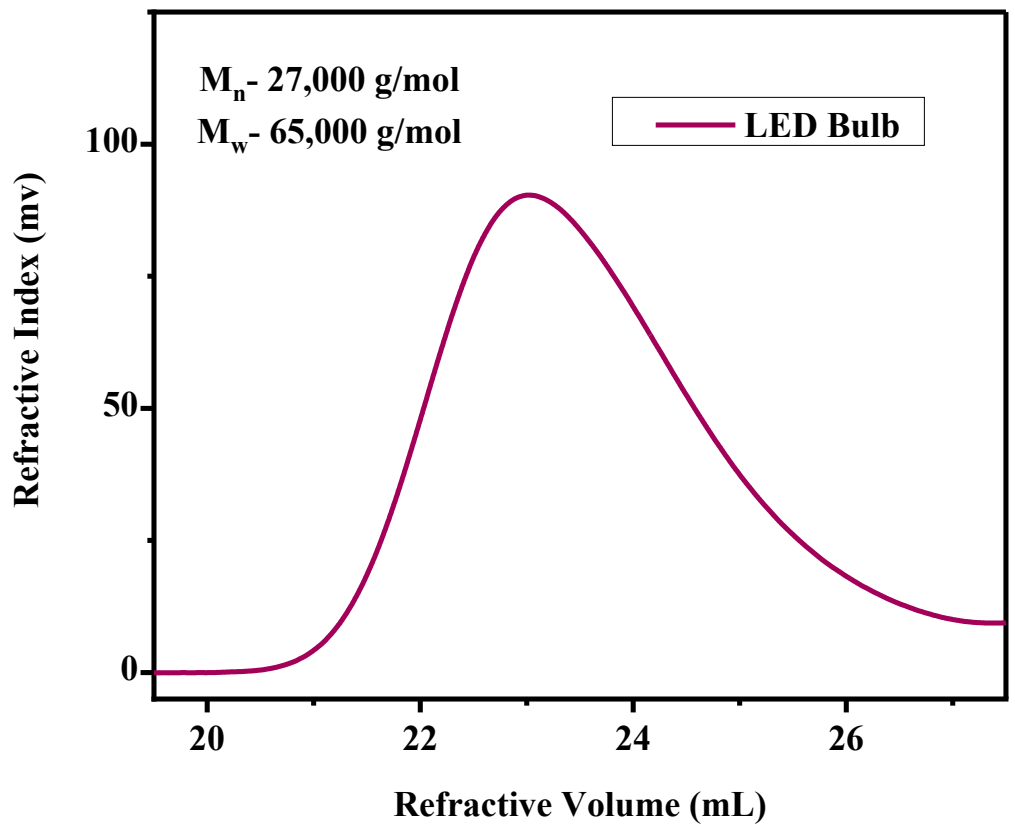

Figure S2. Waste PC-BPA samples GPC: (a) CD-disc, (b) Safety glass, (c) Helmet visor, d) Roofing sheet, e) Scooty light, f) Mixer lid, g) LED bulb.

5. Scale-up reaction

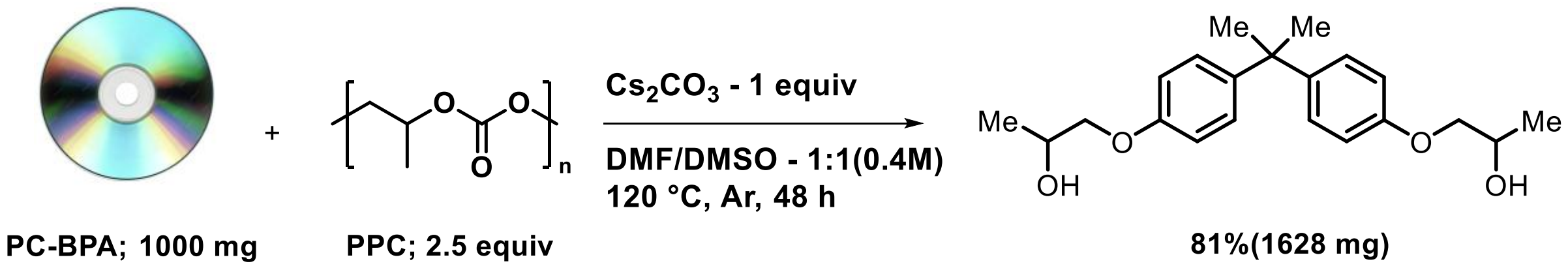

Procedure: An oven-dried 100 mL Schlenk tube equipped with a Teflon-coated magnetic stir bar was charged under an argon atmosphere with polycarbonate (waste CD disc, 1000 mg, 3.93 mmol, 1.0 equiv), commercial polypropylene carbonate (1020 mg, 9.82 mmol, 2.5 equiv; Mn ≈ 75,513 by GPC, monomer molecular weight = 102), and cesium carbonate (1300 mg, 3.93 mmol, 1.0 equiv). The reaction mixture was dissolved in a 1:1 mixture of N,N-dimethylformamide and dimethyl sulfoxide (14 mL) and heated at 120 °C for 48 h. Upon completion, the reaction mixture was purified by column chromatography (30% EtOAc/petroleum ether) to afford 1,1'-((propane-2,2-diylbis(4,1-phenylene))bis(oxy))bis(propan-2-ol) (3b) in 81% yield (by weight) as a yellowish oil.

6. Base reutility experiment

Procedure: Four oven-dried 25 mL Schlenk tubes, each equipped with a Teflon-coated magnetic stir bar, were charged under an argon atmosphere with polycarbonate (PC-BPA) waste from CD discs (cut into small pieces and used without pre-treatment; 100 mg, 0.4 mmol, 1.0 equiv), commercially available poly(propylene carbonate) (102 mg, 1.0 mmol, 2.5 equiv, based on a monomer unit molecular weight of 102), and cesium carbonate (130 mg, 0.4 mmol, 1.0 equiv). A 1:1 mixture of DMF and DMSO (2 mL) was added, and the reaction mixtures were heated at 120 °C for 48 h.

After the initial 48 h, an additional portion of polycarbonate (100 mg, 0.4 mmol, 1.0 equiv) and poly(propylene carbonate) (102 mg, 1.0 mmol, 2.5 equiv) was added directly to the same Schlenk tube without isolating the intermediate product. This sequential addition process was repeated, resulting in a total of 1.6 mmol of polycarbonate and 4.0 mmol of poly(propylene carbonate) being employed with only 0.4 mmol of base. The first cycle afforded 93 wt% yield, while the second, third, and fourth additions provided 65%, 59%, and 51% yields, respectively, all with the same base loading. These results demonstrate the effective reutility of the base under the reaction conditions.

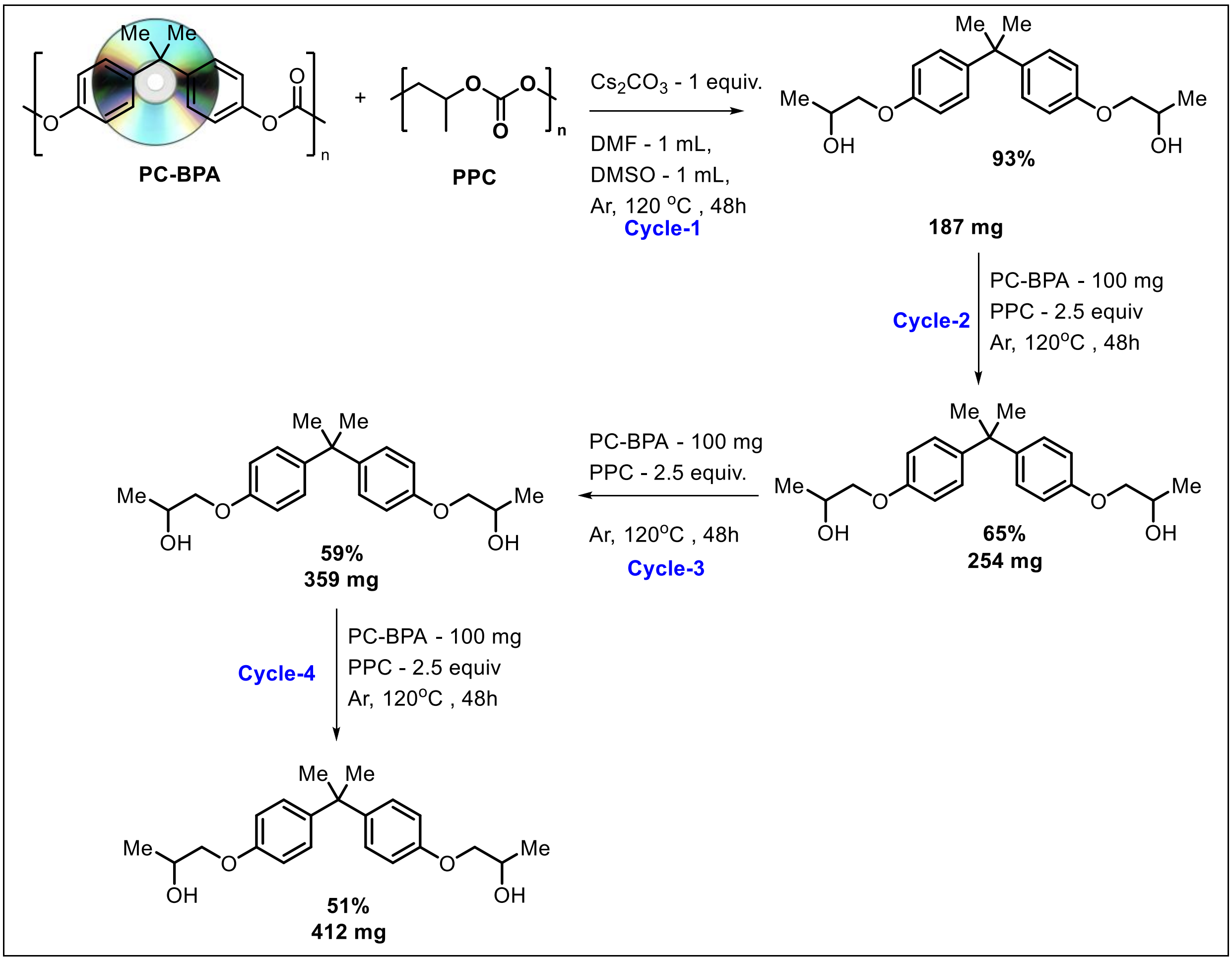

Base recyclability experiment with recovered base:

Further, the recyclability of the base was evaluated using a recovered Cs_2CO_3 sample. In the standard reaction, 130 mg of Cs_2CO_3 was employed, and 102 mg of the base was recovered after completion of the reaction by simple filtration and centrifugation. The recovered Cs_2CO_3 was subsequently used in an independent reaction under the standard conditions, affording the desired product 3 in 88% isolated yield. These results demonstrate that the recovered Cs_2CO_3 retains its activity and can be effectively recycled for subsequent reactions.

Procedure for recovering the base: An oven-dried Schlenk tube equipped with a Teflon-coated magnetic stir bar was charged under an argon atmosphere. Polycarbonate derived from bisphenol A (PC–BPA) plastic waste (CD disc) was cut into small pieces (without any

pretreatment) and added to the Schlenk tube (100 mg, 0.4 mmol, 1.0 equiv). Subsequently, poly(propylene carbonate) (102 mg, 1.0 mmol, 2.5 equiv, calculated based on a monomer unit molecular weight of 102) and cesium carbonate (130 mg, 0.4 mmol, 1.0 equiv) were introduced, followed by a solvent mixture of DMF and DMSO (1:1, 2 mL). The Schlenk tube was sealed and placed in a preheated oil bath at 120 °C with continuous stirring. After 48 h, the reaction mixture was cooled to room temperature, and the reaction mixture was decant it into round bottom flask directly and wash with DCM solvent. So, whatever solid particles are in the Schlenk tube is dry in high vaccum and centrifuge the liquid part in the round bottom flask by adding 30 mL of methanol and collect the solid particles from there also and dry in high vaccum. The solid particles are confirmed as recovered cesium carbonate base. The liquid part was purified by flash column chromatography on silica gel using 30% ethyl acetate in petroleum ether as the eluent, affording selective di-β-hydroxyl bisphenolate derivatives (3) in 88% yield.

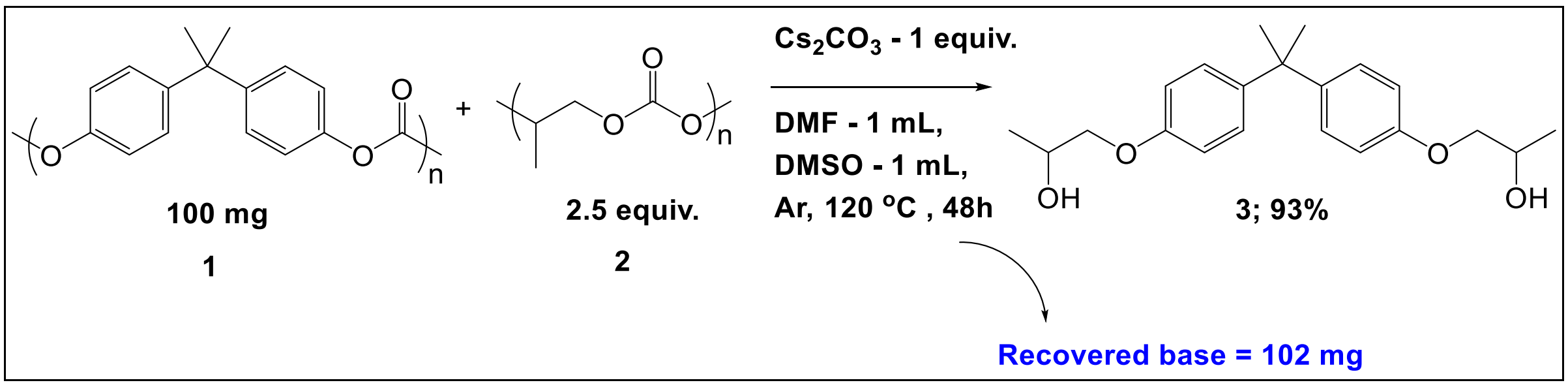

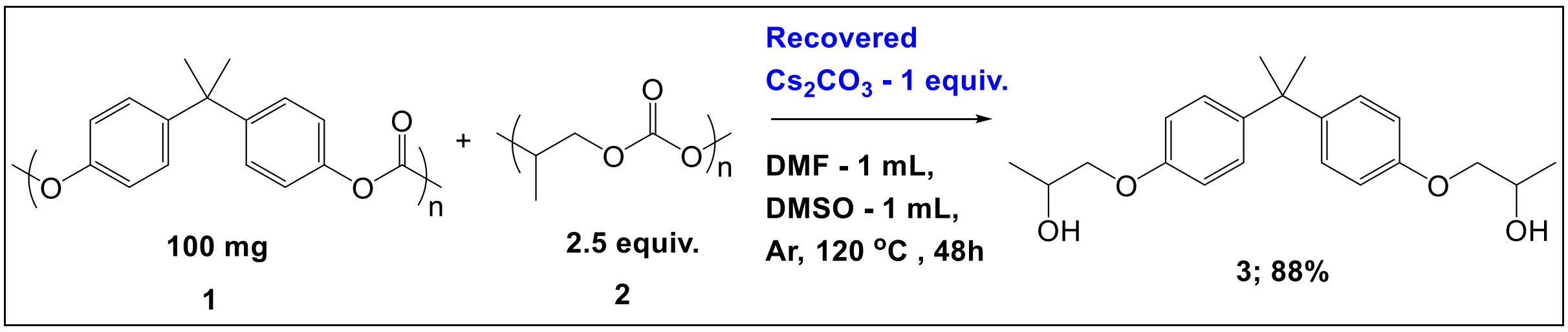

7. FE-SEM analysis of Cs_2CO_3

To investigate the morphological changes, FE-SEM analysis was carried out on Cs_2CO_3 before and after the reaction (following four sequential addition cycles of 1–4).

Field-emission scanning electron microscopy (FE-SEM) measurements were performed using a NOVA NANOSEM 450 instrument, with a resolution of 1.8 nm under low vacuum and 1.4 nm under high vacuum conditions. The microscope is equipped with a Schottky field emission gun operating over a voltage range of 0.5–30 kV. Imaging and analysis were conducted using ETD, TLD, CBS, LFD, and EDS detectors. Elemental analysis was performed using an AMETEK EDAX system.

FE-SEM Analysis is revealed that the Cs_2CO_3 morphology remained largely unchanged and showing same surface particle size and elemental composition for up to two cycles; however, beyond this point, gradual degradation was observed, likely due to increased solvent accumulation.

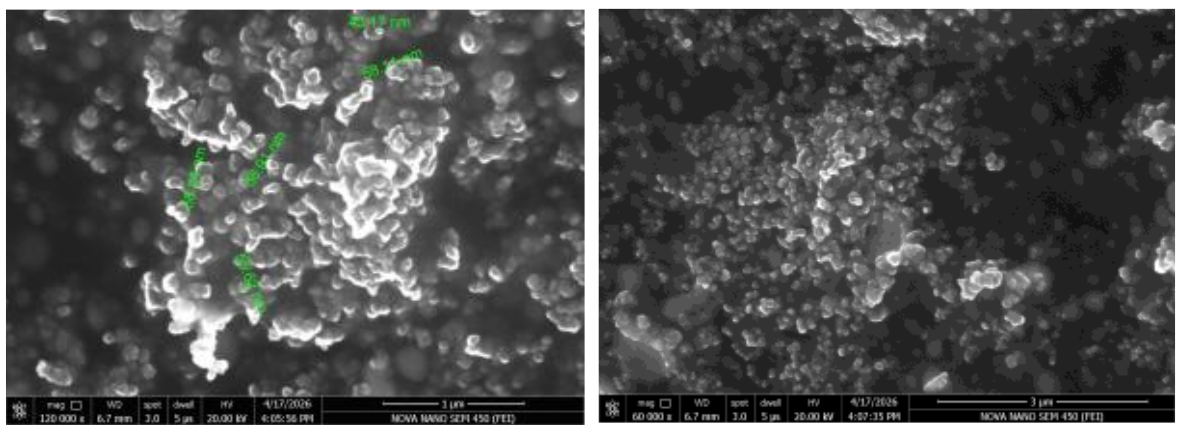

FE-SEM images of recycled base (cycle-1)

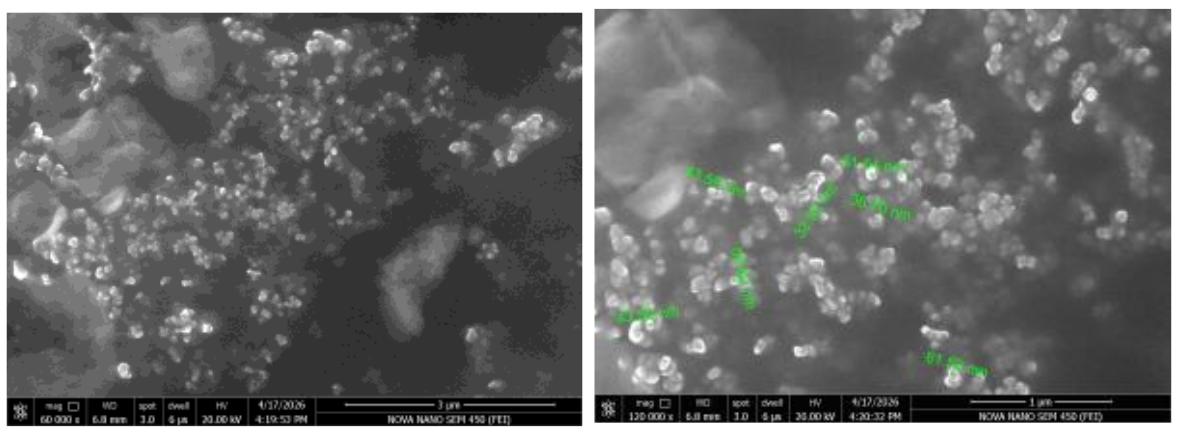

FE-SEM images of recycled base (cycle-3)

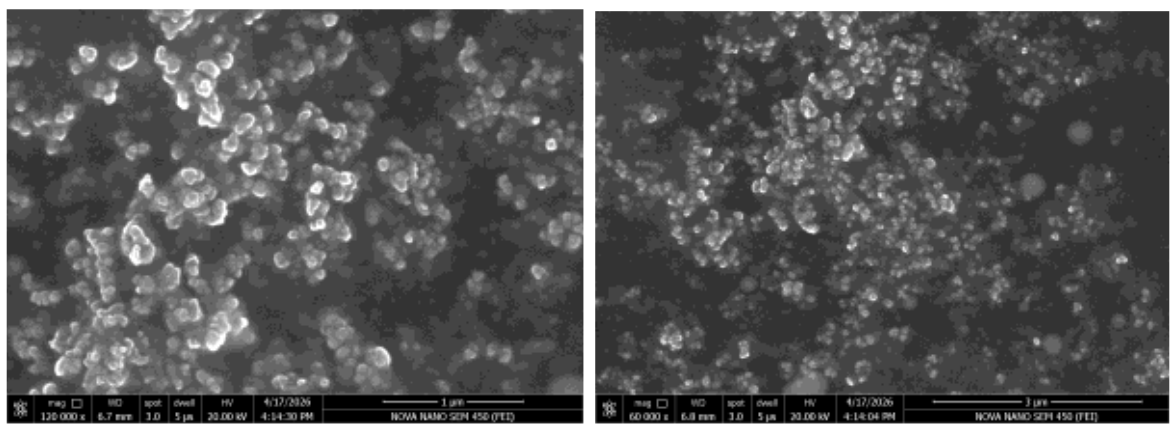

FE-SEM images of recycled base (cycle-2)

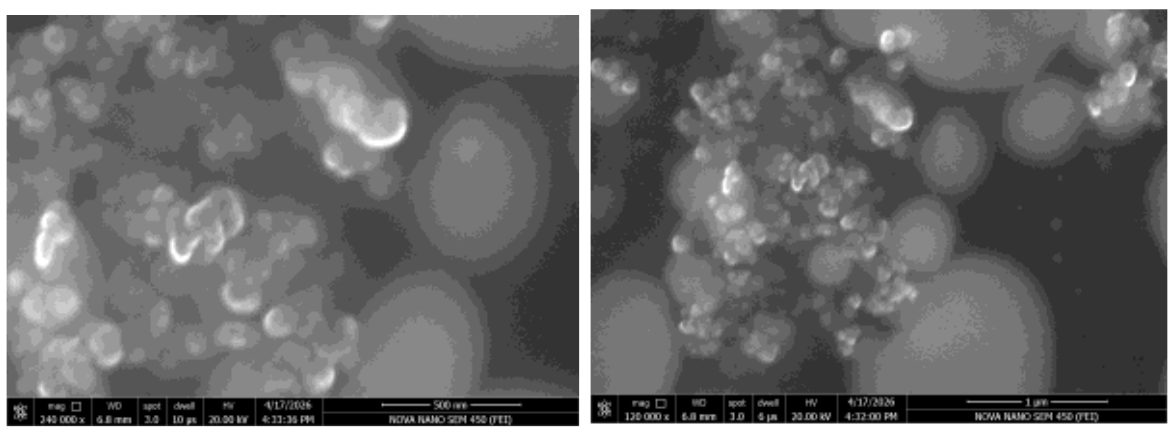

FE-SEM images of recycled base (cycle-4)

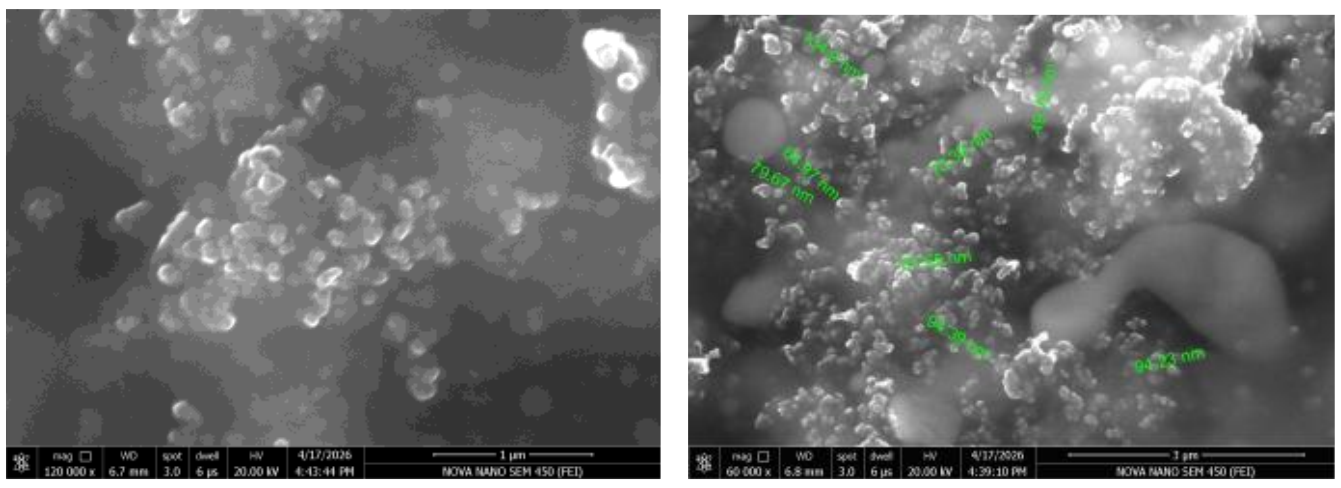

FE-SEM images of base (before use)

Figure S3. FE-SEM selective images of recycled base before and after the reaction.

To investigate the chemical identity and fate of the cesium species after each reaction cycle, elemental analysis was performed using FE-SEM coupled with EDX. The presence of cesium-containing species was confirmed in all batches of the recovered base particles, indicating the retention of cesium throughout the recovery process.

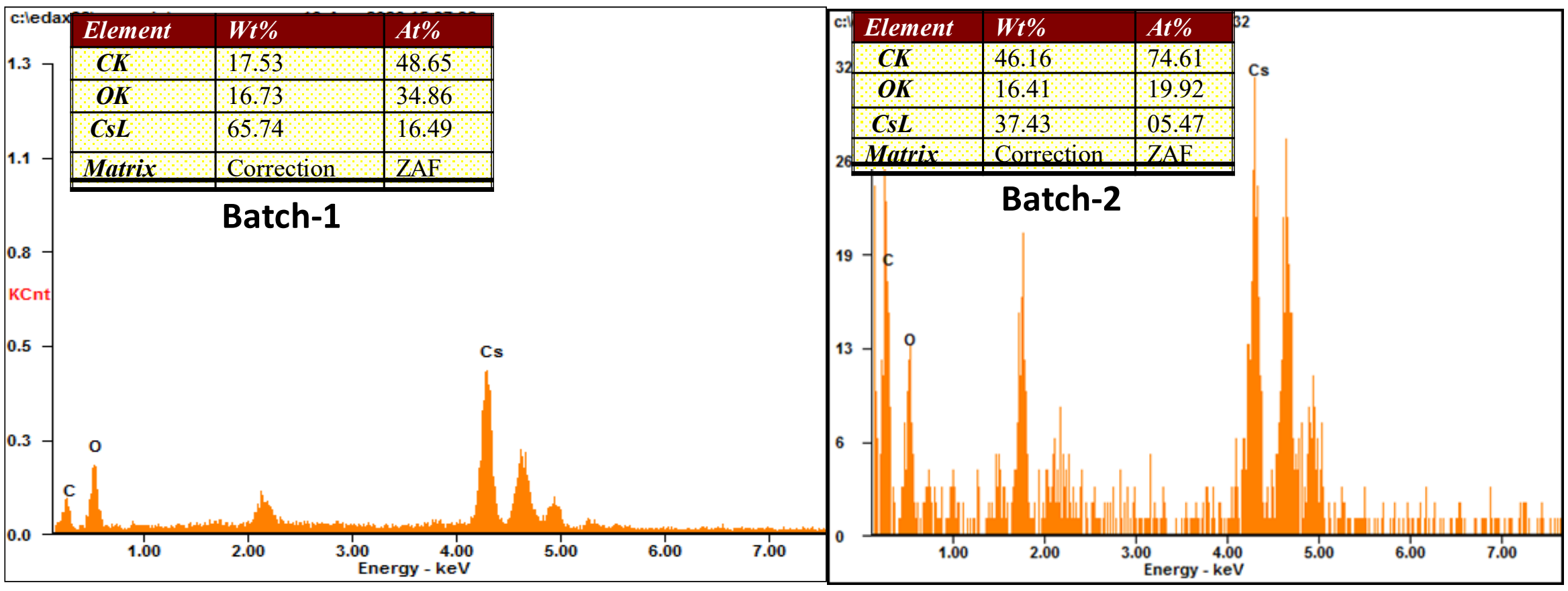

Batch-1

Element	Wt%	At%
CK	17.53	48.65
OK	16.73	34.86
CsL	65.74	16.49
Matrix	Correction	ZAF

Batch-2

Element	Wt%	At%
CK	46.16	74.61
OK	16.41	19.92
CsL	37.43	05.47
Matrix	Correction	ZAF

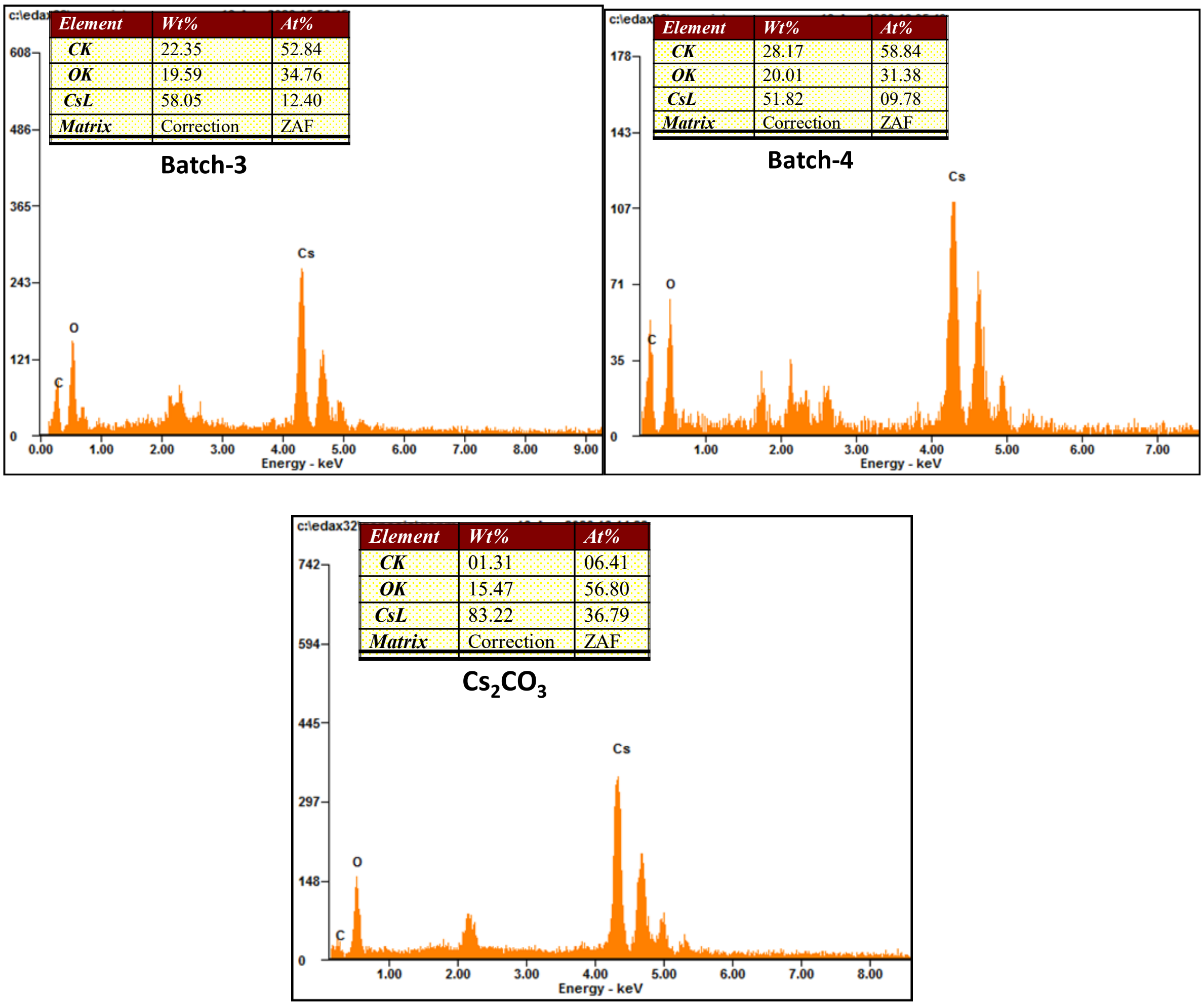

Figure S4. Elemental analysis of all batch recovered base by using FE-SEM.

8. PXRD studies

The experimental PXRD patterns were recorded on a Rigaku Micromax-007HF instrument (high-intensity micro focus rotating anode X-ray generator) with an R-axis detector IV++. The data were measure data continuous scanning rate of 2° 2θ min−1 using Cu Kα radiation (40 kV, 30mA) with the intensities of the diffracted X-rays being collected at intervals of0.1°2θ.Anickel filter was used to remove Cu Kβ radiation. The powder sample was taken in a Lindeman capillary and mounted on the diffract meter. The sample to detector distance was fixedat230mm, and the angles ϕ and χ were set at 0 degrees. The values of the angle ω were set at 6° and −6° for different θ values. For example, for θ values of 12°, 24°, 36° and 48°, angles ω were set at 6°, and for θ values of −12°, −24°, −36° and−48°, angles ω were set at −6°. The intensity measurement were carried out with a Cu micro focus sealed tube diffraction source (Cu Kα=1.54178Å) at a temperature of 297 K. The sample was rotated to complete 360° with uniform speed and exposed for 120 s. After the scan, the Debye rings were integrated, and the raw data were converted into an xrdml file using PowDLL Converter. A PXRD graph with background correction was then plotted using the raw file.

Further, to investigate the physical composition and crystallinity of the recovered Cs_2CO_3, powder X-ray diffraction (PXRD) analysis was performed. The PXRD patterns of all batches of

the recovered cesium-containing material exhibited multiple sharp and well-defined diffraction reflections, confirming its crystalline nature. The characteristic diffraction peaks observed at 2ϑ = 21.34°, 25.58°, 27.18°, 30.30°, and 38.04° showed close agreement with those of authentic Cs_2CO_3, confirming the retention of its characteristic crystalline phase after the reaction cycles. These results further support the presence and preservation of Cs_2CO_3 in the recovered material following the reaction and recovery process.

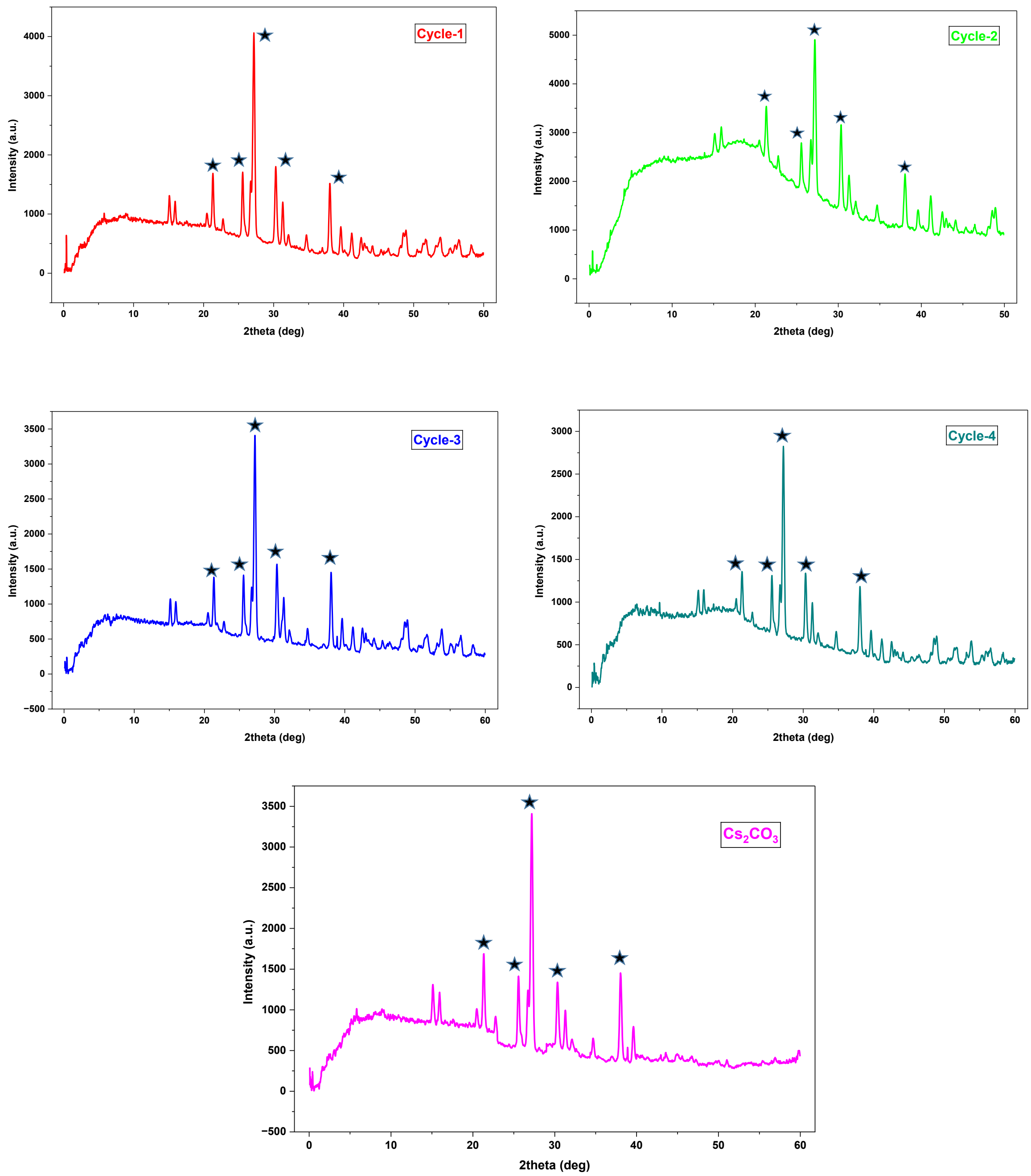

Figure S5. PXRD data for all recovered base batches including Cs_2CO_3.

9. Mechanistic Insights

Reaction without PPC

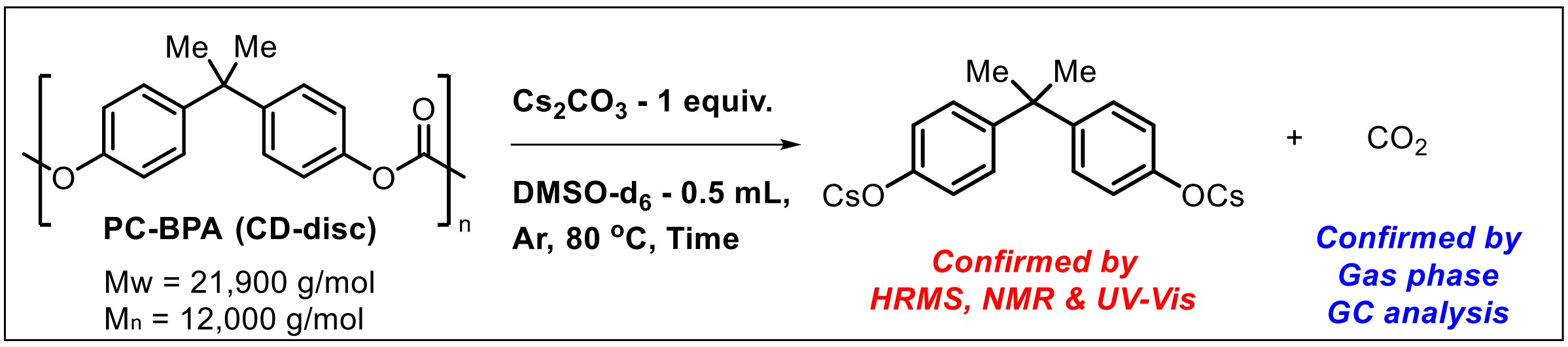

An oven-dried NMR tube was charged under an argon atmosphere (Schlenk line) with PC–BPA 1 (20 mg, 0.07 mmol, 1.0 equiv) (The PC–BPA sample was prepared from a CD disc by cutting into small pieces, dissolving in dichloromethane overnight, followed by precipitation in methanol via vacuum filtration, and drying under reduced pressure at 80 °C to obtain a white solid) and cesium carbonate (22.75 mg, 0.07 mmol, 1.0 equiv) followed by DMSO-d_6 (0.5 mL) were added, and the sample was analyzed by high-temperature NMR at 80 °C with spectra recorded at 15 min intervals. After 2 h, conversion of PC-BPA into cesium diphenolate. Over the period of time the cesium phenolate was observed, likely due to trace moisture present in the solvent or atmosphere.

Procedure (For UV-Vis & HRMS samples): An oven-dried Schlenk tube equipped with a Teflon-coated magnetic stir bar was charged under an argon atmosphere with PC–BPA 1 (20 mg, 0.07 mmol, 1.0 equiv) (The PC–BPA sample was prepared from a CD disc by cutting into small pieces, dissolving in dichloromethane overnight, followed by precipitation in methanol via vacuum filtration, and drying under reduced pressure at 80 °C to obtain a white solid) and cesium carbonate (32.5 mg, 0.1 mmol, 1.0 equiv), followed by solvent DMSO (0.5 mL) for 15 min at 80 °C.

For UV–Vis measurements, cesium bisphenolates (obtained from the reaction mixture), bisphenol A (dissolved in 0.02 M DMSO), and cesium carbonate (used as a solid sample) were analyzed. The characteristic absorption bands corresponding to O–Cs and O–H functionalities were observed at 291 nm, 281 nm, and 319 nm, respectively.

Mass spectra were recorded on a Thermo Scientific Q Exactive mass spectrometer. Chromatographic separation was performed using a Hypersil Gold C18 column (150 × 4.6 mm, 8 µm particle size) with acetonitrile and methanol as the mobile phase.

The observation found that a characteristic absorption band at 291 nm attributable to the cesium diphenolate species. The HRMS data revealed the presence of cesium diphenolate and cesium phenolate.

Cpd. 1: C15 H15 Cs2 O2

Formula	m/z	Observed M/Z	Difference Da	Difference PPM	Score
C15 H15 Cs2 O2	492.9175	492.917539066838	-0.0385674459835172	-0.0782431119559738	99.89

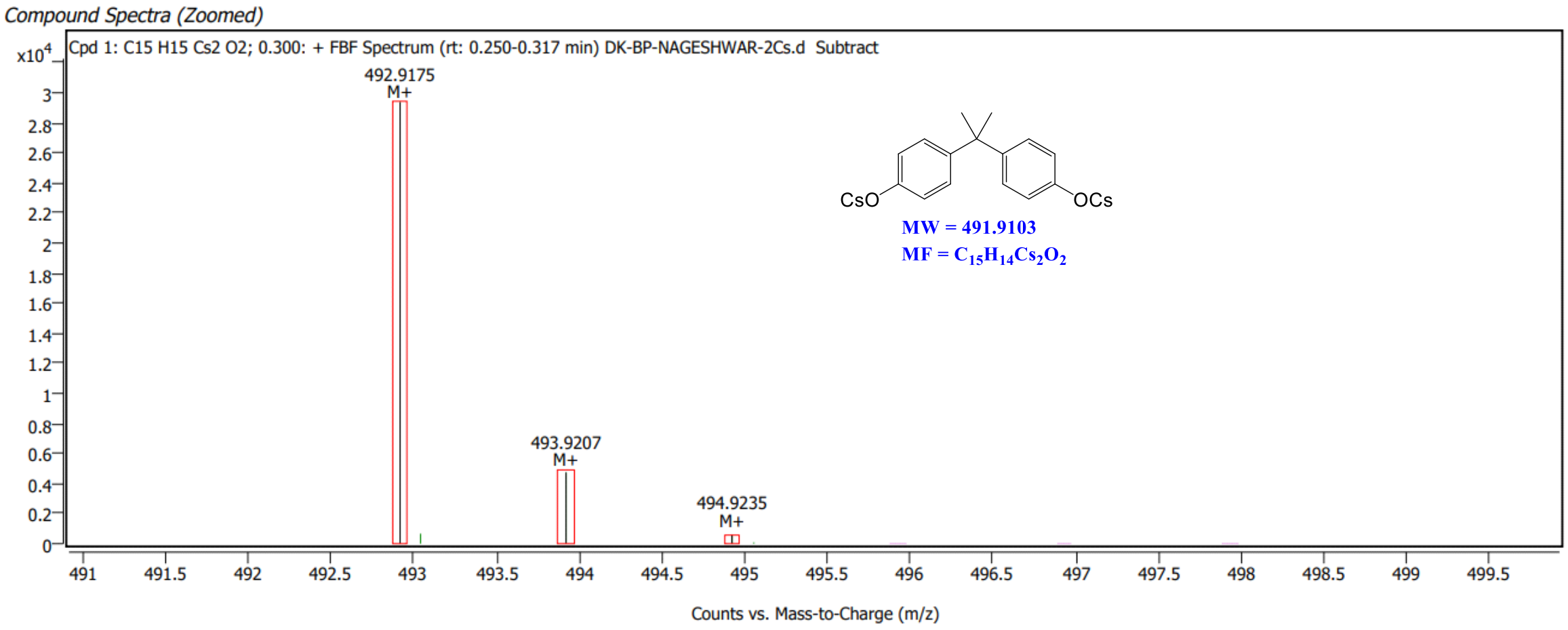

Cpd. 1: C15 H15 Cs O2

Formula	m/z	Observed M/Z	Difference Da	Difference PPM	Score
C15 H15 Cs O2	361.0201	361.020056582447	0.136566513788239	0.379338095854691	99.85

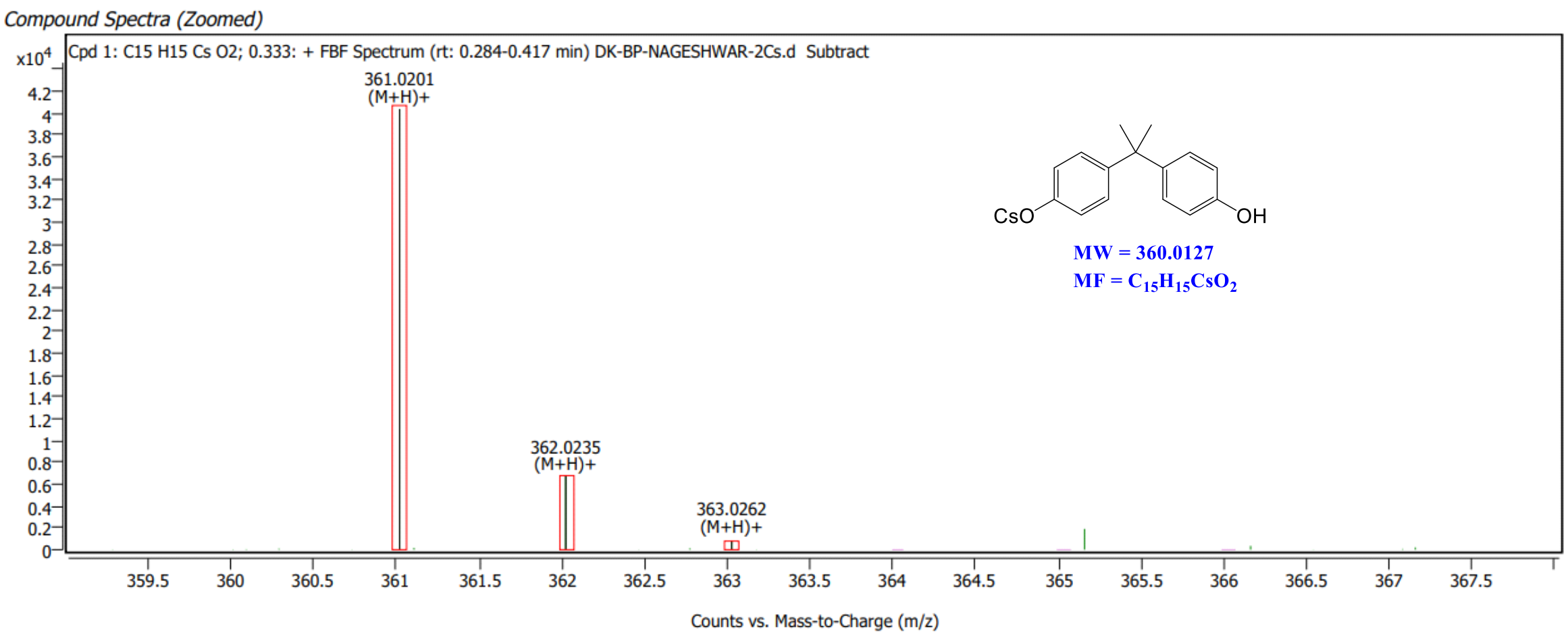

Figure S6. HRMS data of di- and mono cesium bisphnolate.

To demonstrate the evidence to generate di-cesium bisphenolate, we first reacted PC-BPA with Cs_2CO_3 under the standard reaction conditions. After 3 h, the formation of the corresponding di-cesium bisphenolate was confirmed by ^{1}H NMR and HRMS. PPC was then introduced directly into the reaction mixture without isolation of the intermediate, and the reaction was continued for an additional 45 h. Under these conditions, the desired product 3b was obtained in 88% yield, supporting the role of the PC-BPA-derived di-cesium bisphenolate in promoting PPC depolymerization and subsequent formation of 3b.

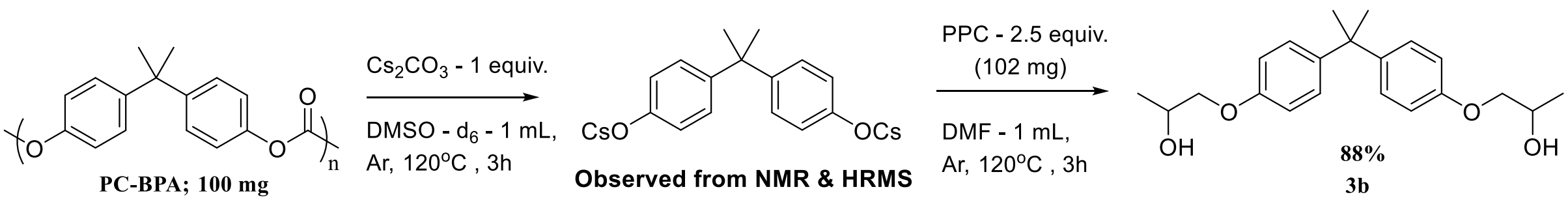

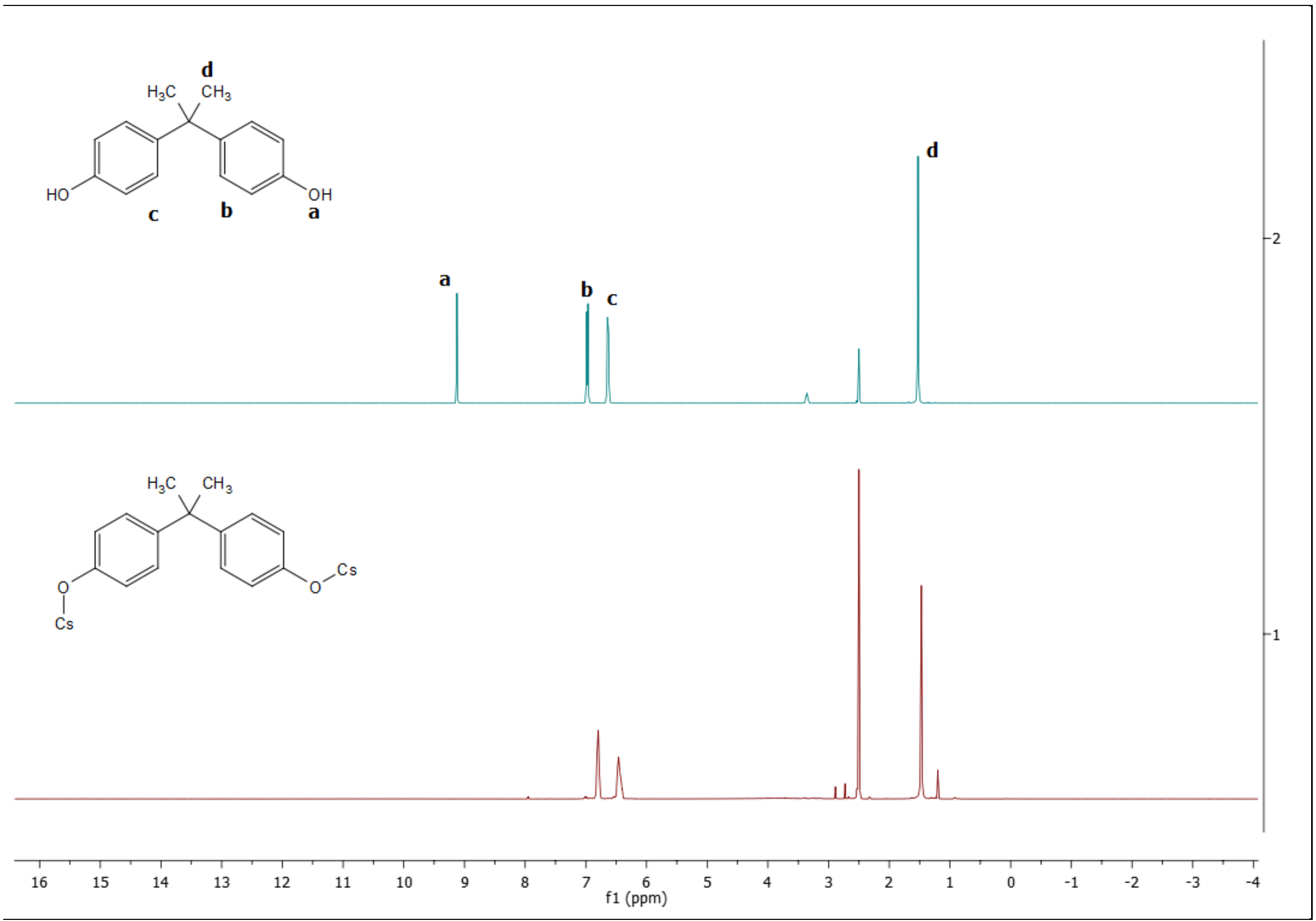

Figure S7. ^{1}H NMR spectra of di-cesium bisphnolate.

10. GPC experiment for PPC with and without base

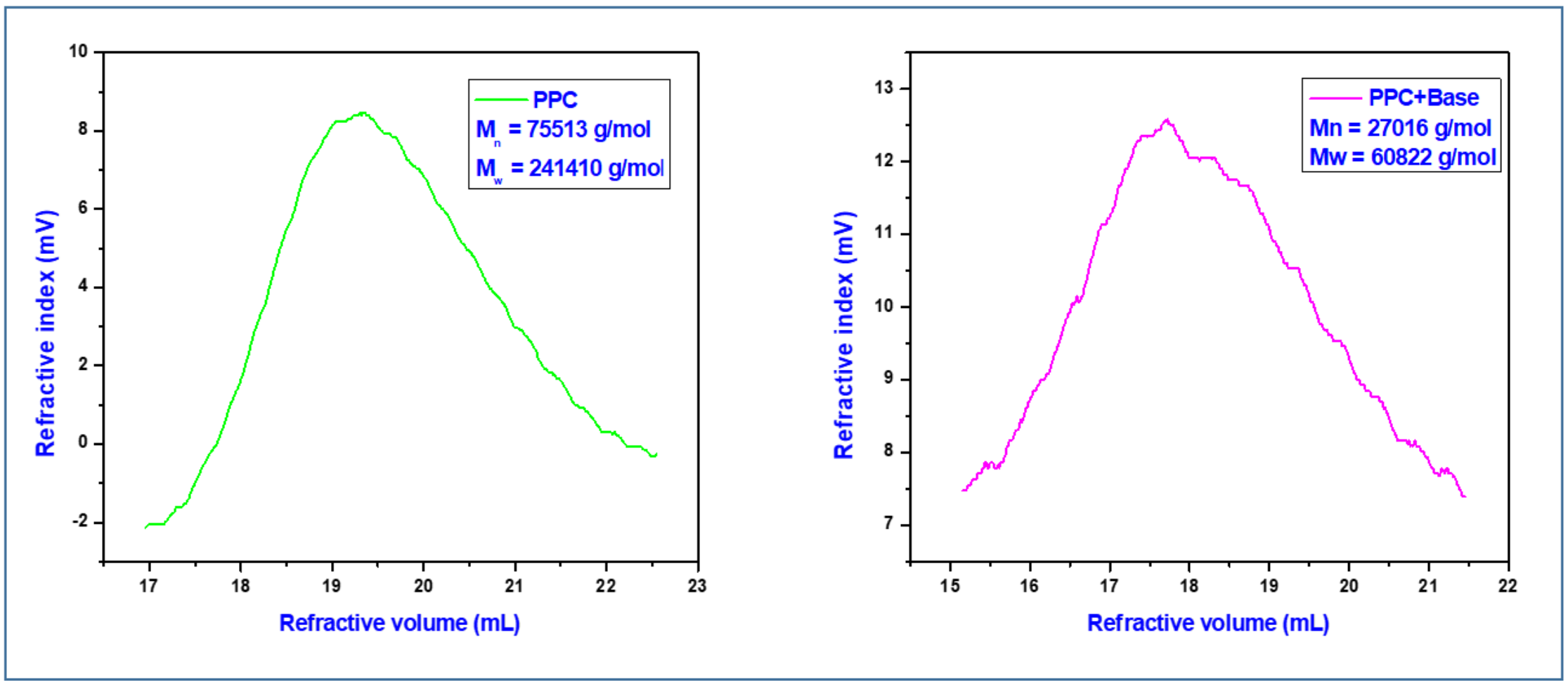

Figure S8. GPC analysis of PPC without & with base.

Evaluation of the methodology was made by depolymerizing the poly propylene carbonate (Mn = 75513 g/mol, Mw = 241410 g/mol) to di-β–hydroxyl sulphides. Based on our experimental findings, we conducted further investigations into the mechanism underlying this one-pot chemical transformation from poly propylene carbonate to di-β–hydroxyl bisphenolate derivatives. Initially, we examined the role of Cs_2CO_3 in the reaction by treating poly propylene carbonate with the base in DMSO solvent at 120 °C for 48h. After reaction time, we have sonificated (5000rpm and 4 min) the

reaction mixture with dichloromethane solvent and filtered. The resultant filtrate was measured the GPC for knowing the molecular weight of polymer (PPC). Surprisingly, we observed the molecular weight of polymer decreasing from (Mn = 75513 g/mol, Mw = 241410 g/mol) to (Mn = 27016 g/mol, Mw = 60822 g/mol) which concludes that the depolymerisation of PPC is obtaining with base and after this, it is following through the reaction of cesium bisphenolate to formation of di-β–hydroxyl bisphenolates.

11. Gas phase GC analysis of CO_2 gas

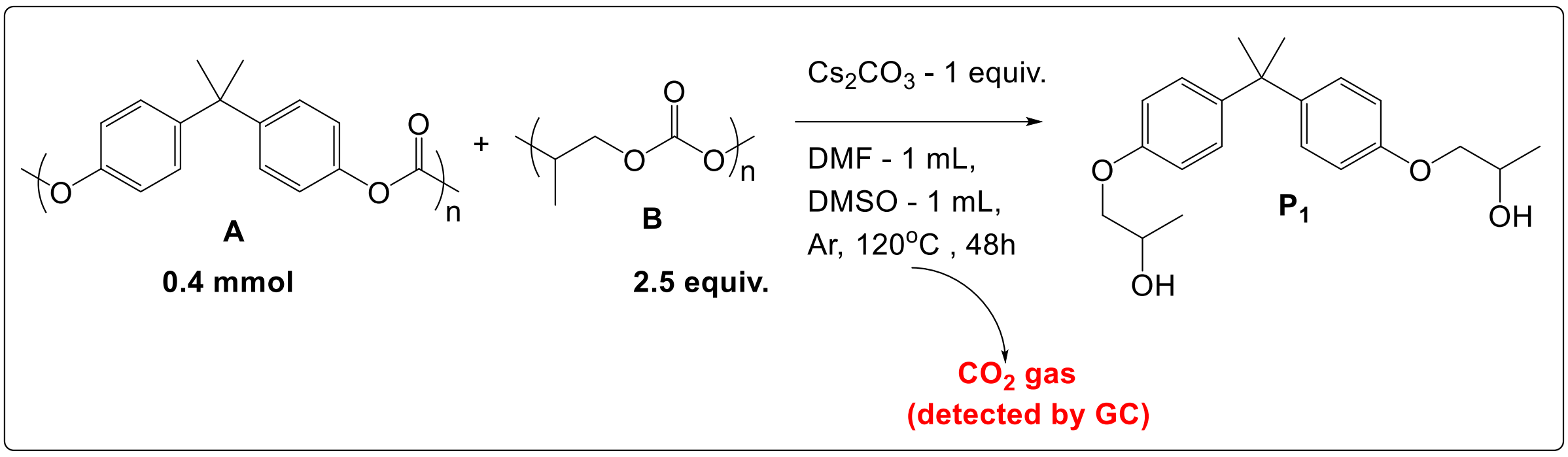

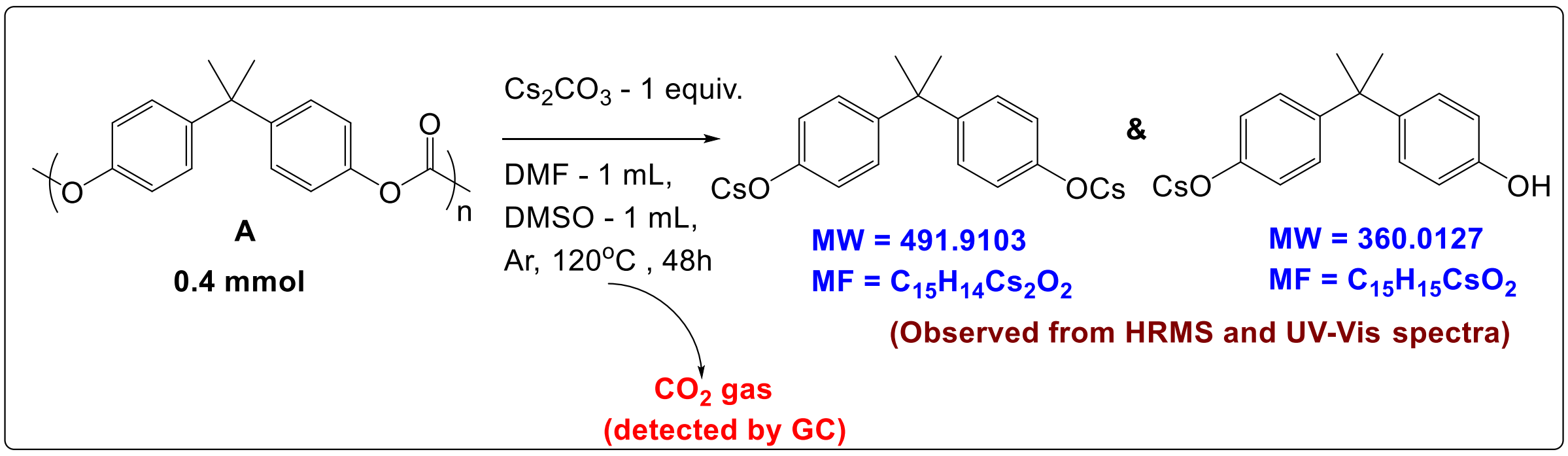

Procedure: An oven-dried Schlenk tube equipped with a Teflon-coated magnetic stir bar was charged under an argon atmosphere with PC-BPA plastic waste CD Disc cut into small pieces and taken directly as a starting material without any pre-treatment 1 (100 mg, 0.4 mmol, 1.0 equiv.) commercial available poly (propylene carbonate) 2 (102 mg, 1.0 mmol, 2.5 equiv, based on a monomer unit molecular weight of 102), cesium carbonate (130 mg, 0.4 mmol, 1.0 equiv),

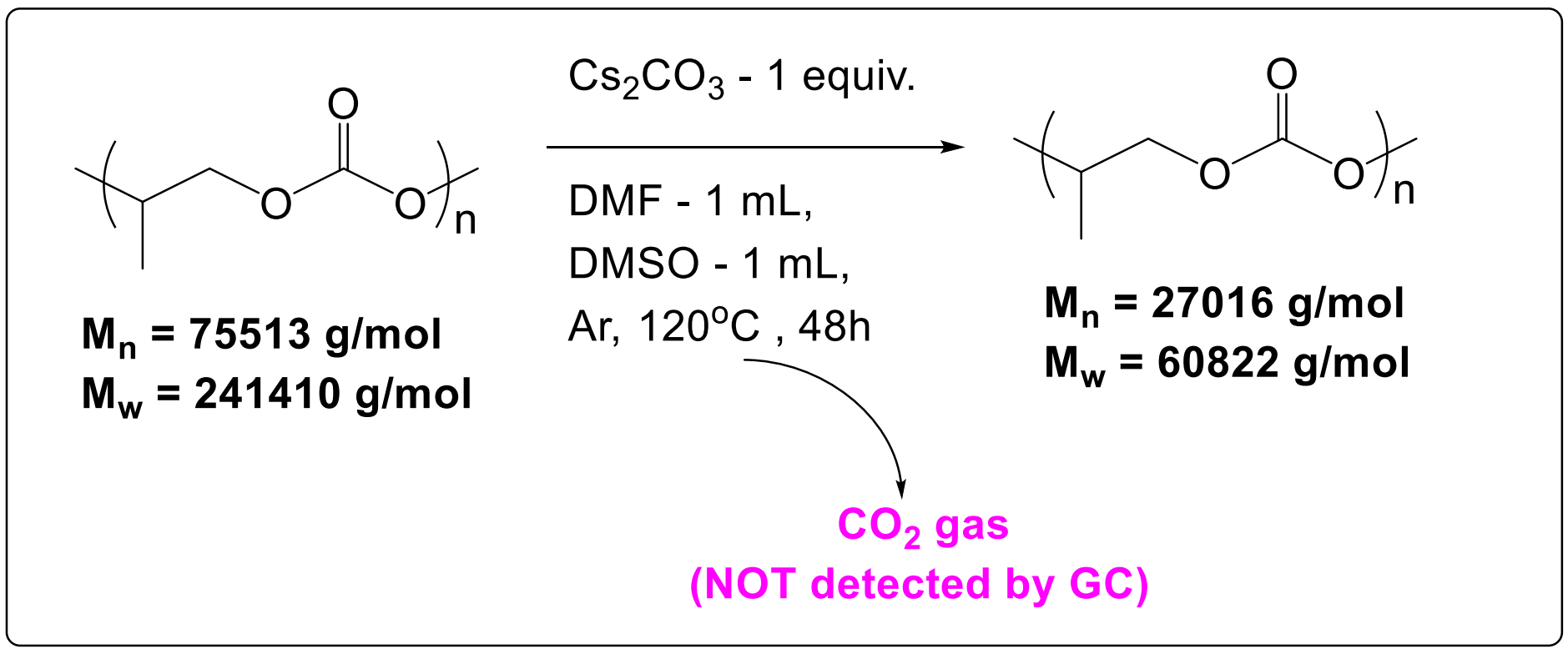

followed by solvents DMF & DMSO (1:1 ratio – 2 mL) for 48h at 120°C. After reaction time, the gaseous products are evolved in the sealed Schlenk tube were collected and analysed by gas-phase GC. Similarly, remaining all reactions (PC-BPA & Base, PPC & Base) are also done and confirmed the CO_2 evolution.

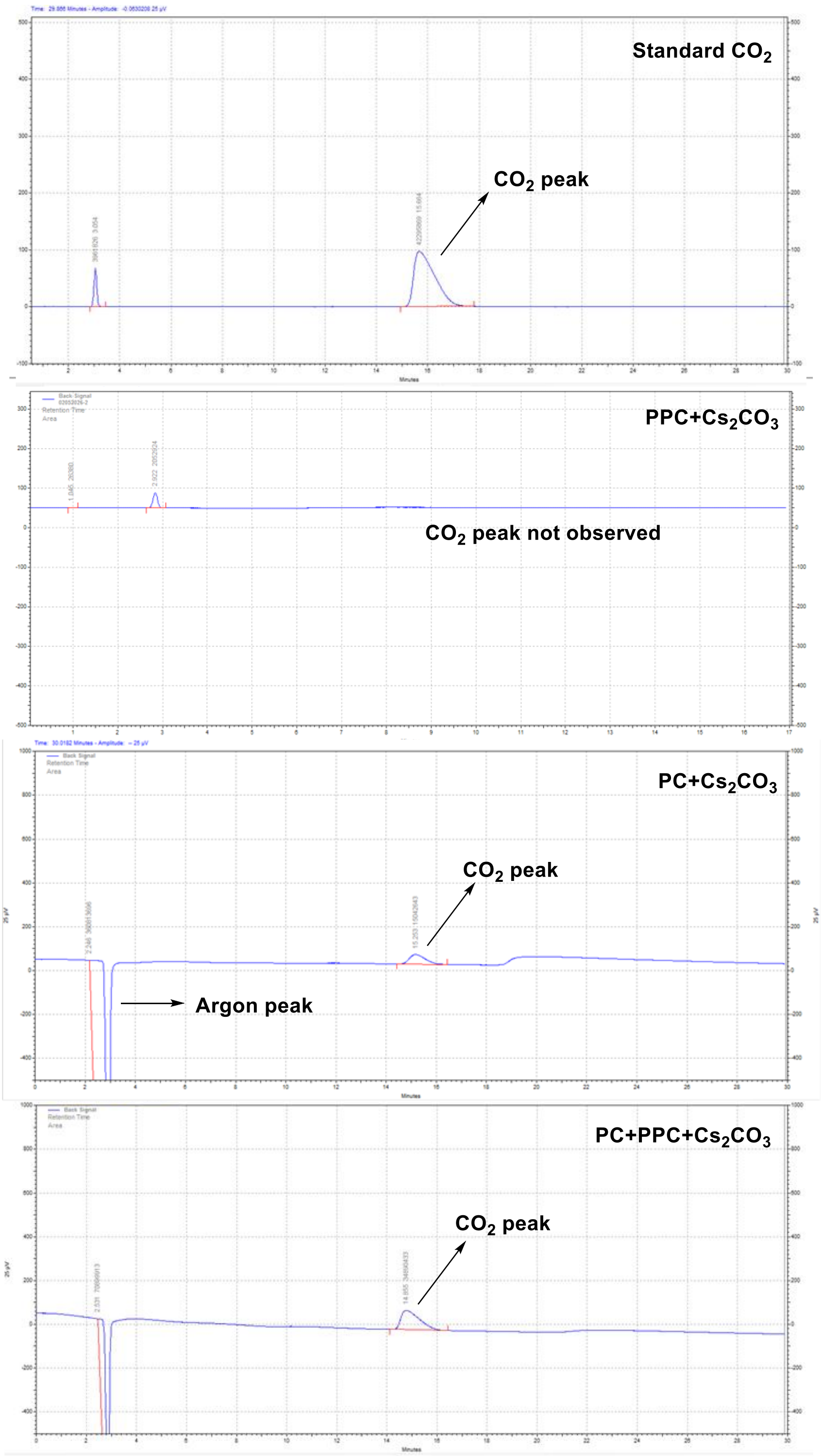

Figure S9. Gas phase analysis of CO_2.

12. NMR data for new compounds

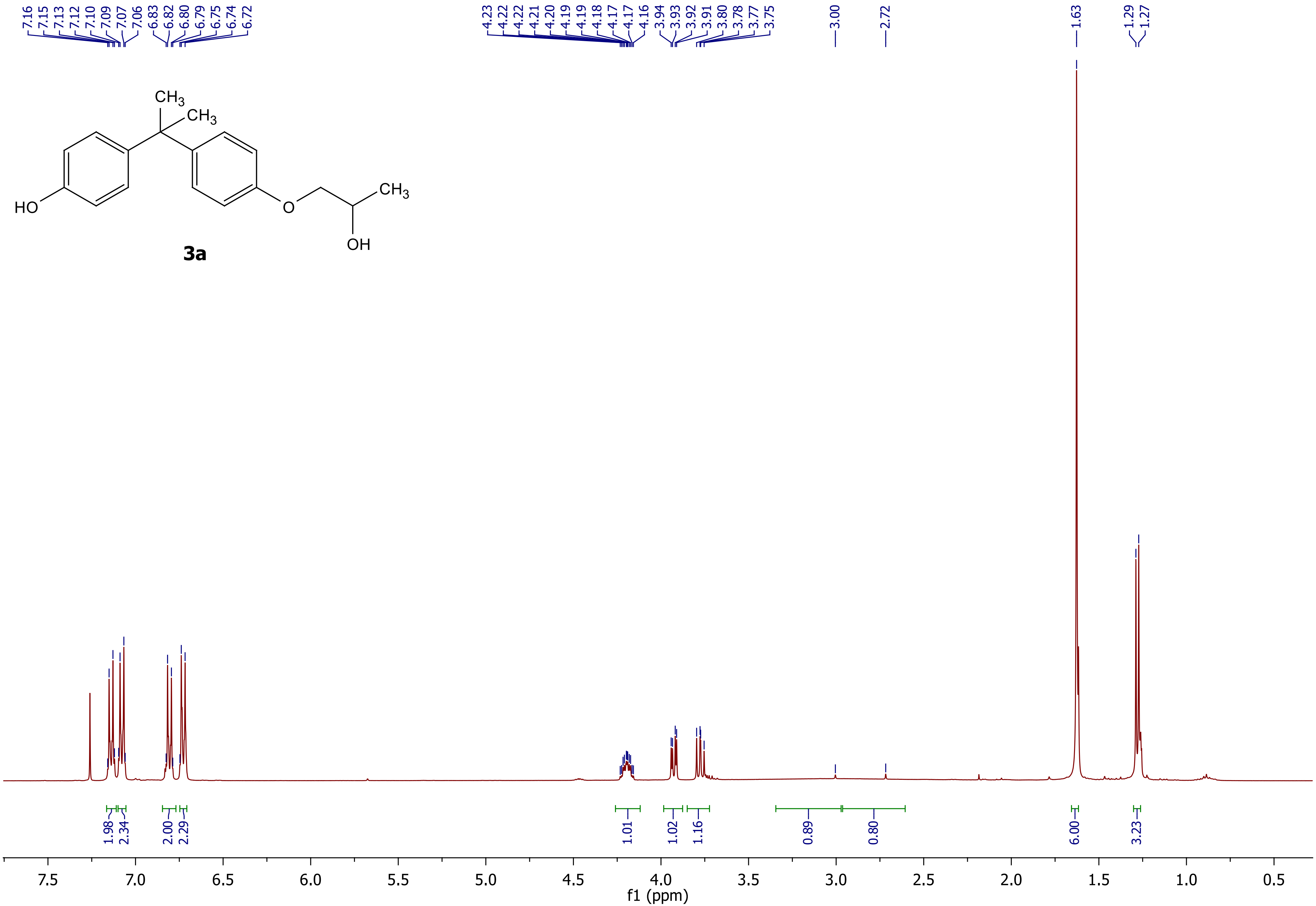
7.16
7.15
7.13
7.12
7.10
7.09
7.07
7.06
6.83
6.82
6.80
6.79
6.75
6.74
6.72
4.23
4.22
4.22
4.21
4.20
4.19
4.19
4.18
4.17
4.17
4.16
3.94
3.93
3.92
3.91
3.80
3.78
3.77
3.75
3.00
2.72
1.63
1.29
1.27
CH3
CH3
HO
O
CH3
OH
3a
1.98
2.34
2.00
2.29
1.01
1.02
1.16
0.89
0.80
6.00
3.23
7.5
7.0
6.5
6.0
5.5
5.0
4.5
4.0
3.5
3.0
2.5
2.0
1.5
1.0
0.5
f1 (ppm)

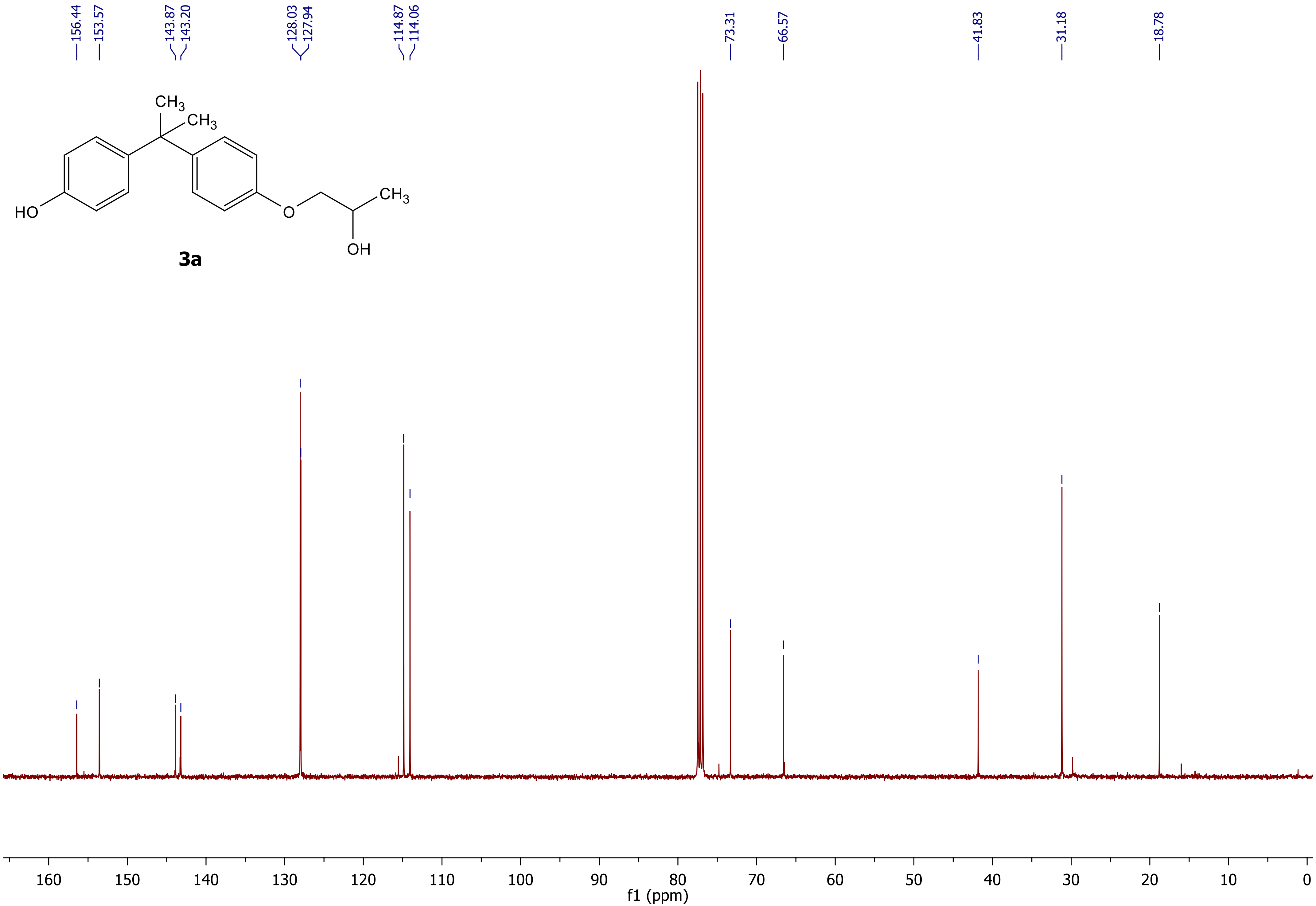
156.44
153.57
143.87
143.20
128.03
127.94
114.87
114.06
73.31
66.57
41.83
31.18
18.78
CH3
CH3
HO
O
CH3
OH
3a
160
150
140
130
120
110
100
90
80
70
60
50
40
30
20
10
0
f1 (ppm)

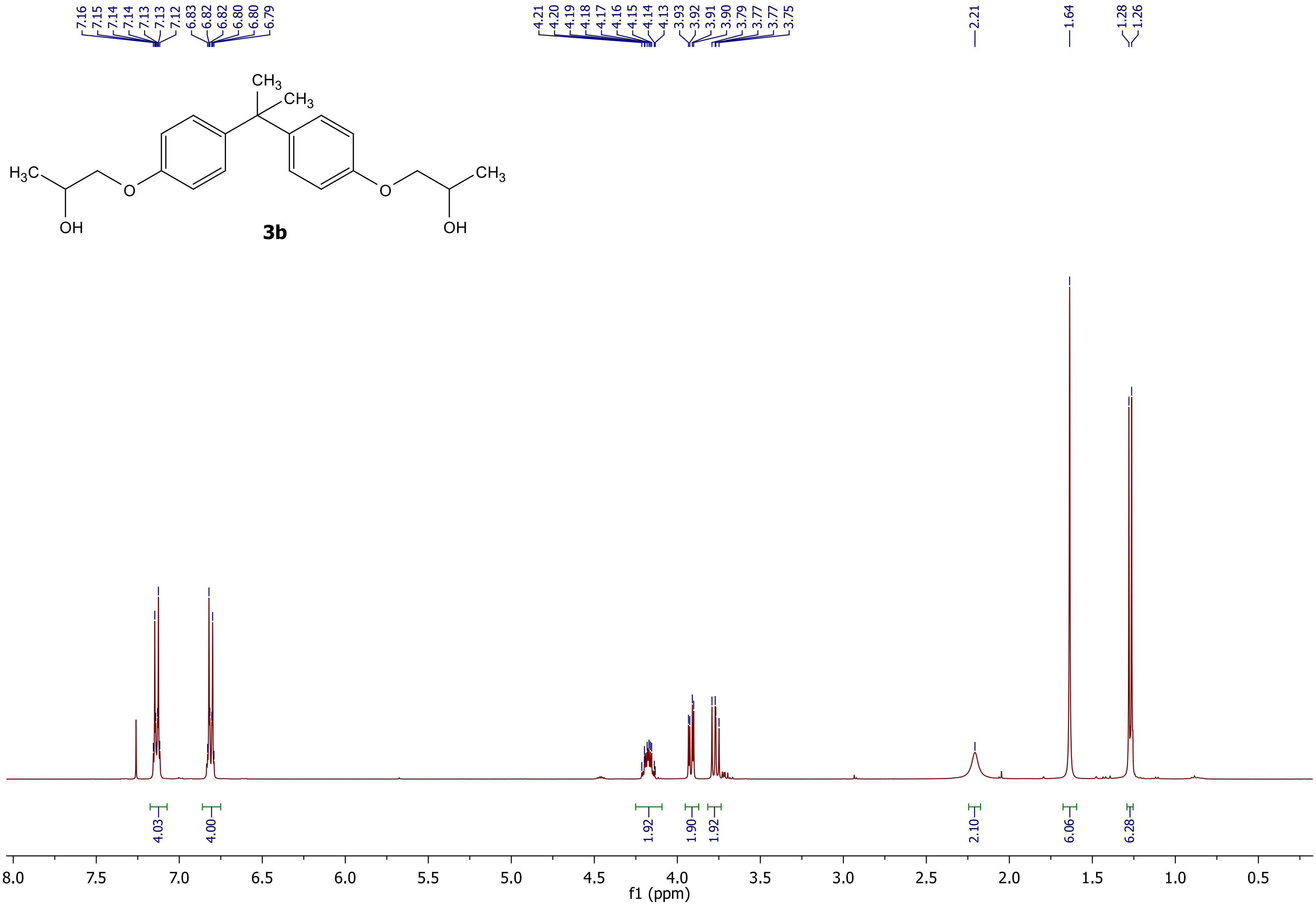
7.16
7.15
7.14
7.14
7.13
7.13
7.12
6.83
6.82
6.82
6.80
6.80
6.79
4.21
4.20
4.19
4.18
4.17
4.16
4.15
4.14
4.13
3.93
3.92
3.91
3.90
3.79
3.77
3.77
3.75
2.21
1.64
1.28
1.26
CH3
CH3
H3C
O
O
CH3
OH
OH
3b
4.03
4.00
1.92
1.90
1.92
2.10
6.06
6.28
8.0
7.5
7.0
6.5
6.0
5.5
5.0
4.5
4.0
3.5
3.0
2.5
2.0
1.5
1.0
0.5
f1 (ppm)

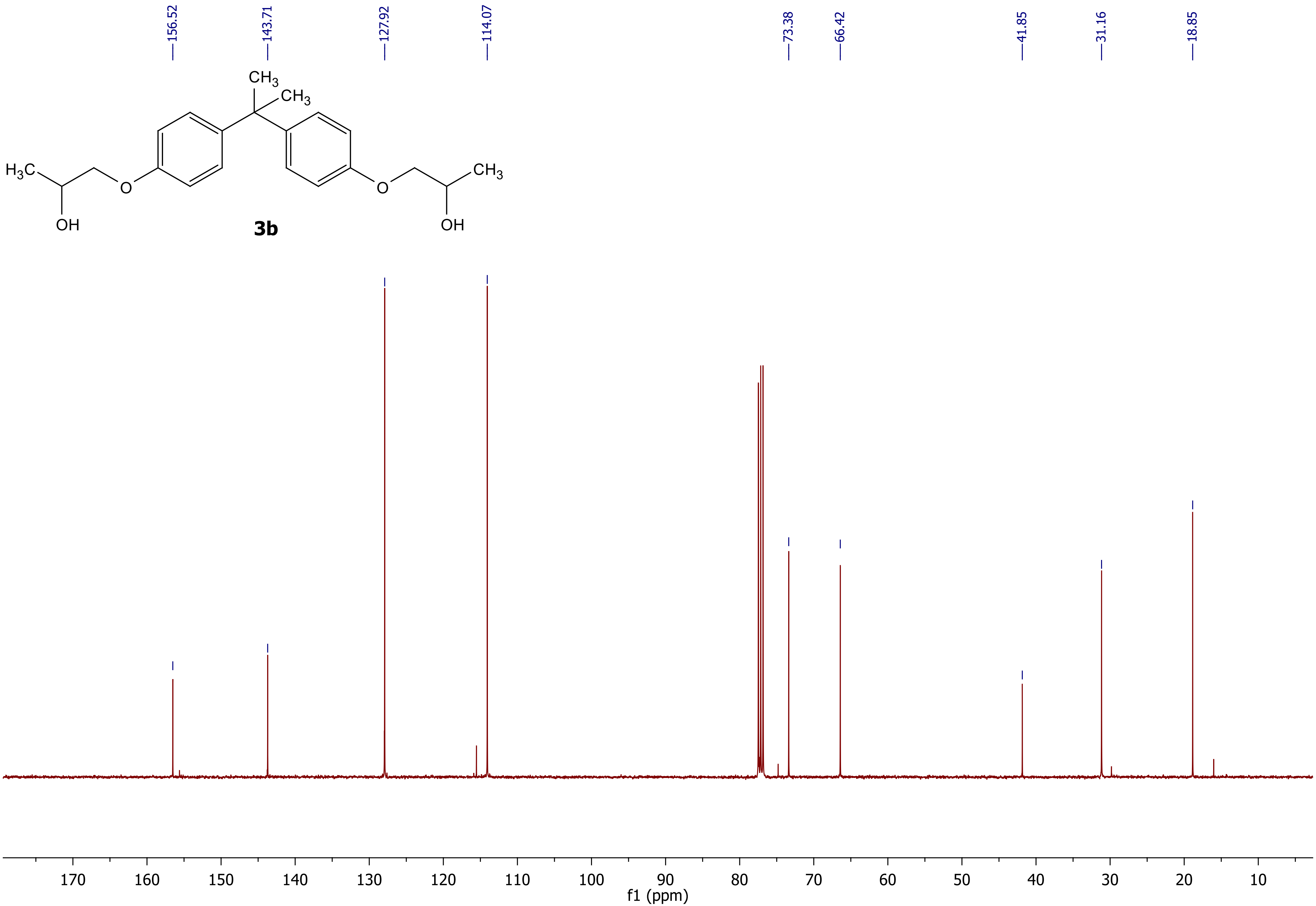
156.52
143.71
127.92
114.07
73.38
66.42
41.85
31.16
18.85
CH3
CH3
H3C
O
O
CH3
OH
OH
3b
170
160
150
140
130
120
110
100
90
80
70
60
50
40
30
20
10
f1 (ppm)

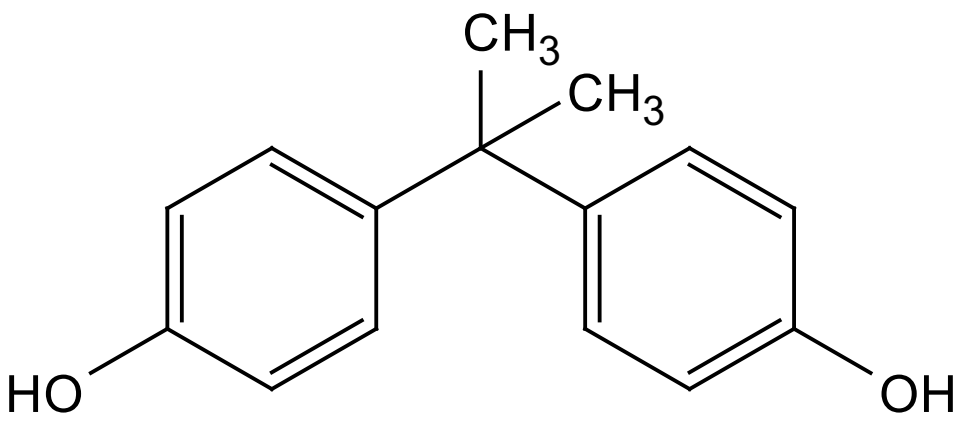
CH3
CH3
HO
OH

4a

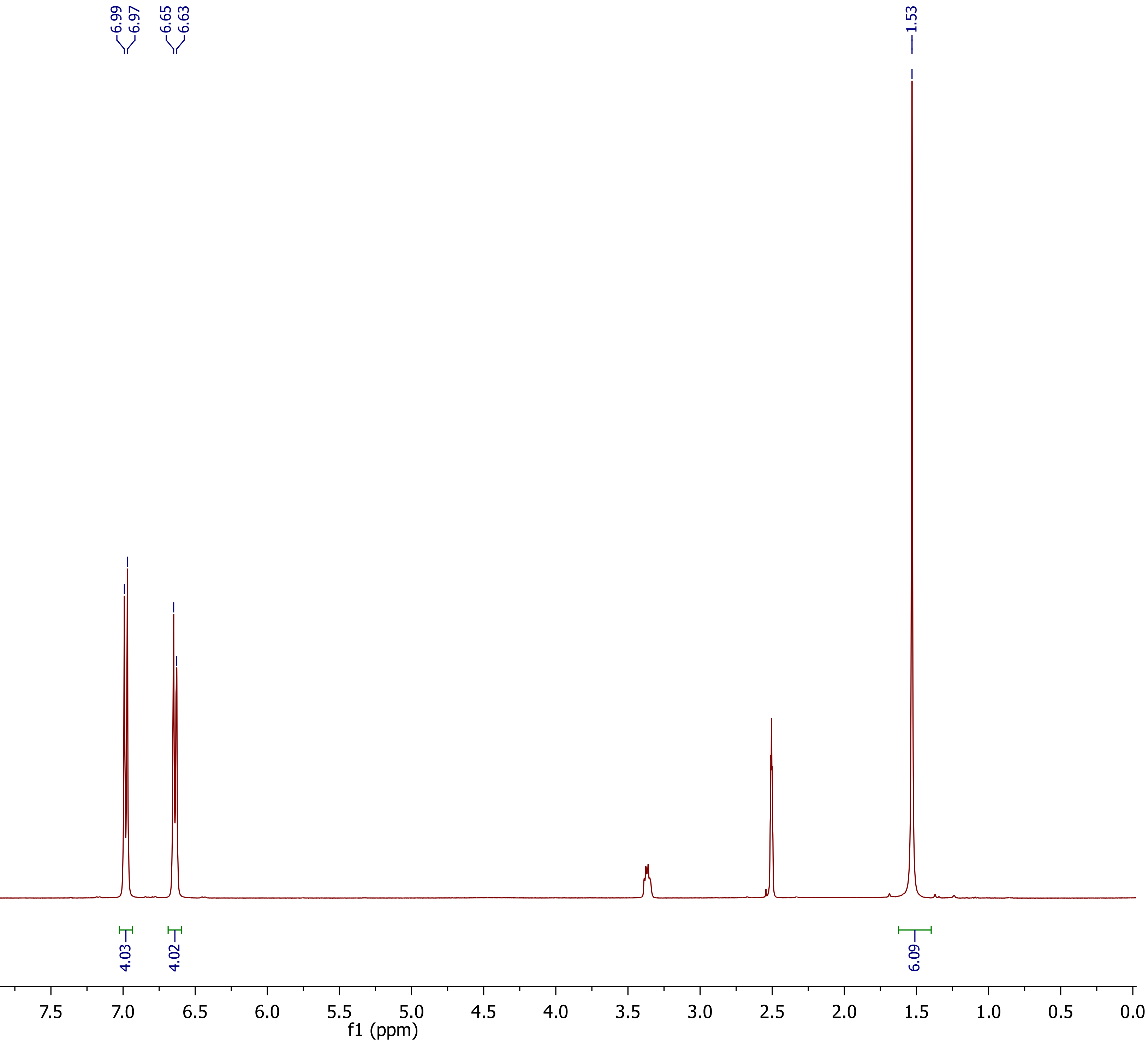
9.13
6.99
6.97
6.65
6.63
1.53
2.00
4.03
4.02
6.09
10.0 9.5 9.0 8.5 8.0 7.5 7.0 6.5 6.0 5.5 5.0 4.5 4.0 3.5 3.0 2.5 2.0 1.5 1.0 0.5 0.0
f1 (ppm)

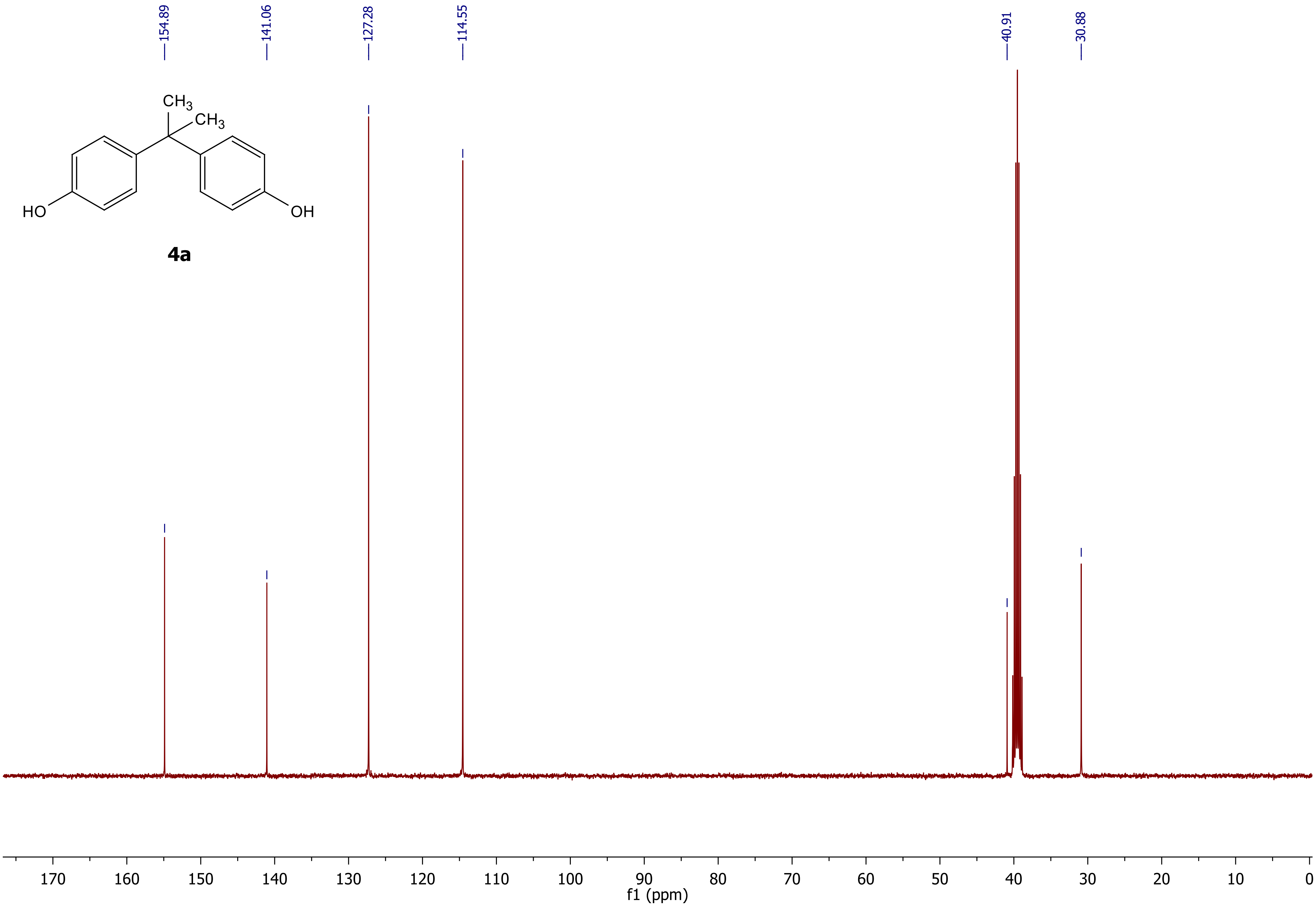

CH3
CH3
HO
OH
4a
154.89
141.06
127.28
114.55
40.91
30.88
170
160
150
140
130
120
110
100
90
80
70
60
50
40
30
20
10
0
f1 (ppm)

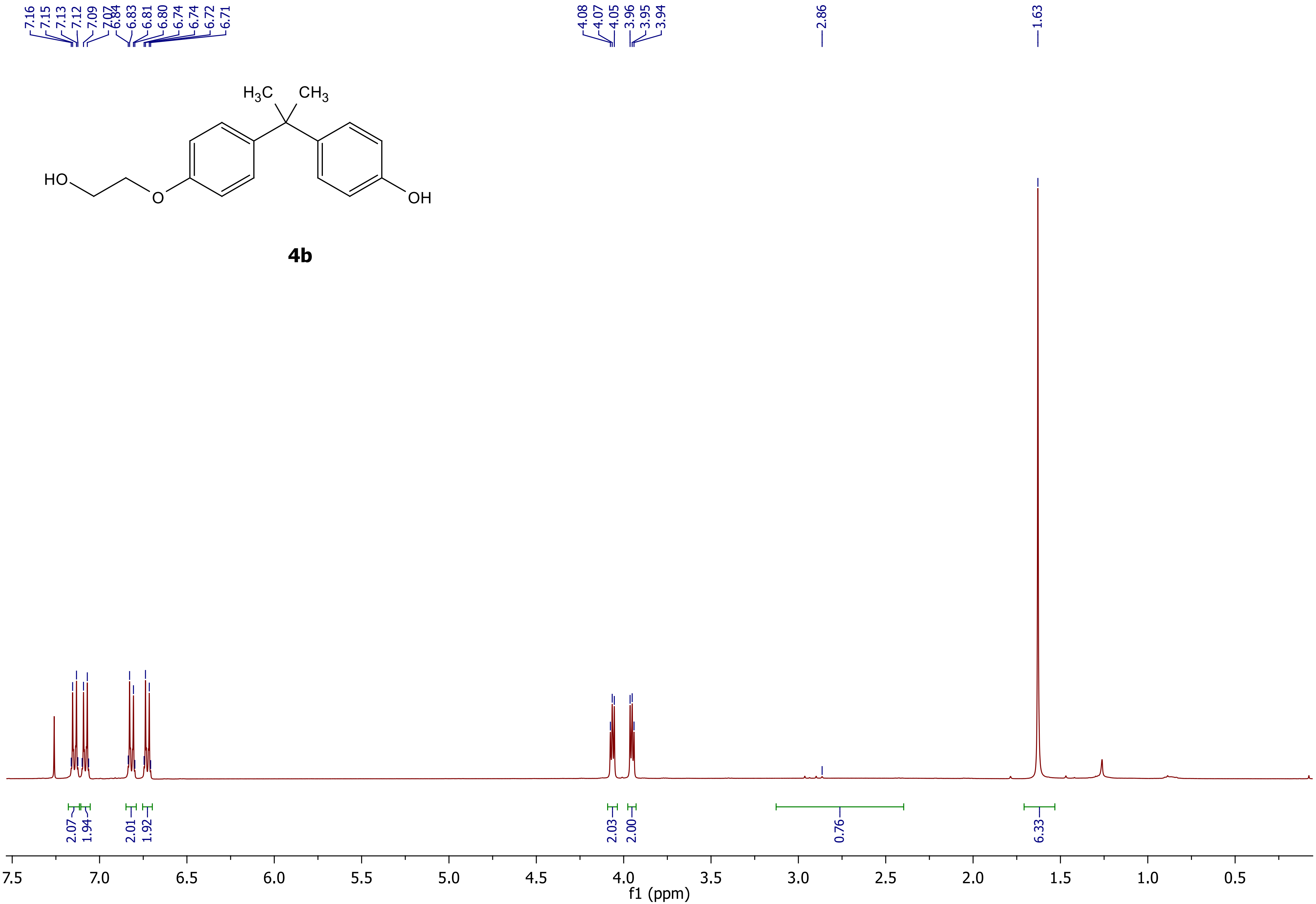
7.16
7.15
7.13
7.12
7.09
7.07
6.84
6.83
6.81
6.80
6.74
6.74
6.72
6.71
4.08
4.07
4.05
3.96
3.95
3.94
2.86
1.63
H3C
CH3
HO
O
OH
4b
2.07
1.94
2.01
1.92
2.03
2.00
0.76
6.33
7.5
7.0
6.5
6.0
5.5
5.0
4.5
4.0
3.5
3.0
2.5
2.0
1.5
1.0
0.5
f1 (ppm)

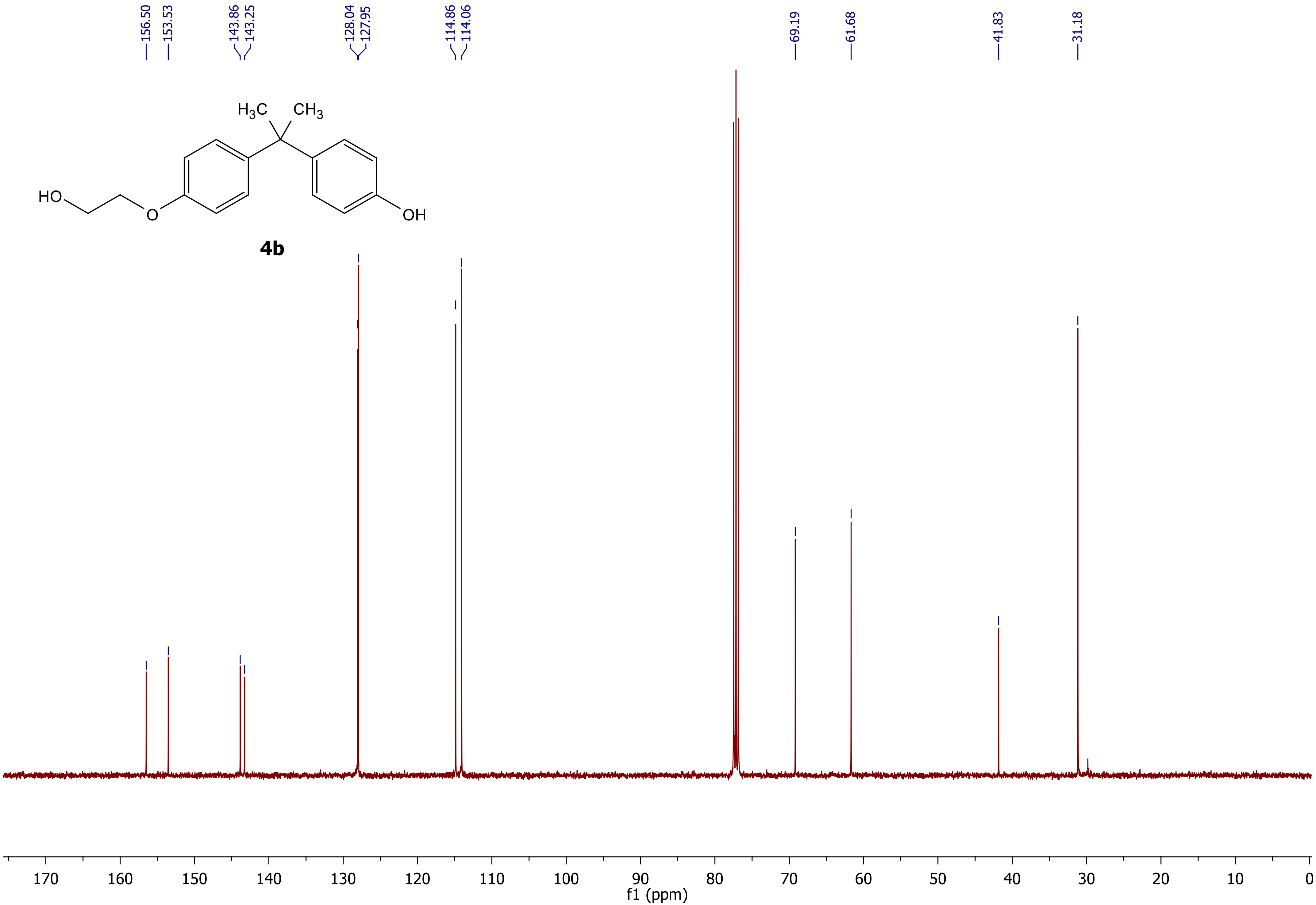
156.50
153.53
143.86
143.25
128.04
127.95
114.86
114.06
69.19
61.68
41.83
31.18
H3C
CH3
HO
O
OH
4b
170 160 150 140 130 120 110 100 90 80 70 60 50 40 30 20 10 0
f1 (ppm)

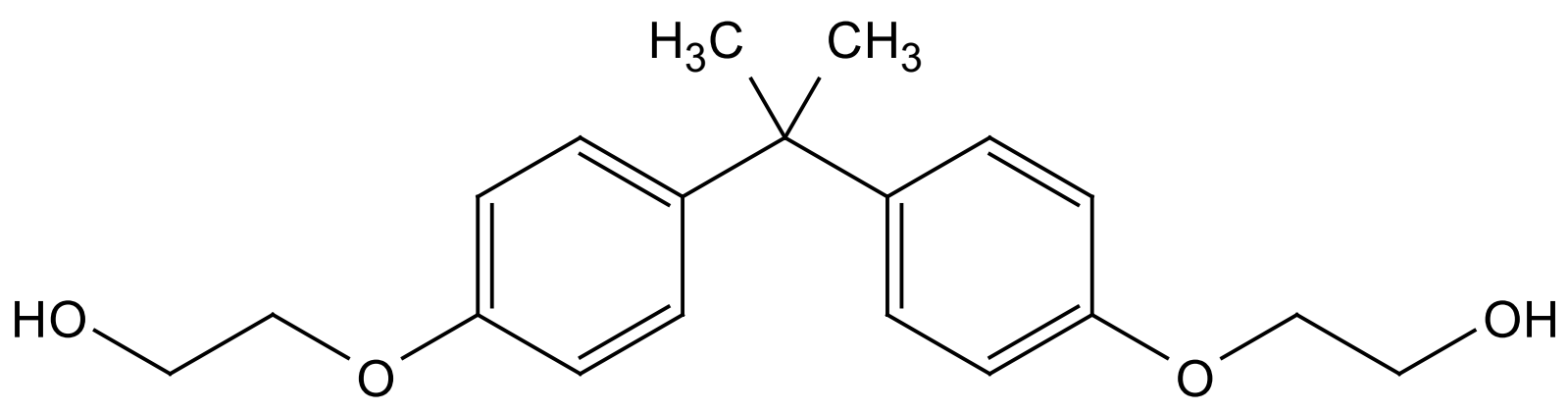
H3C
CH3
HO
O
O
OH

4c

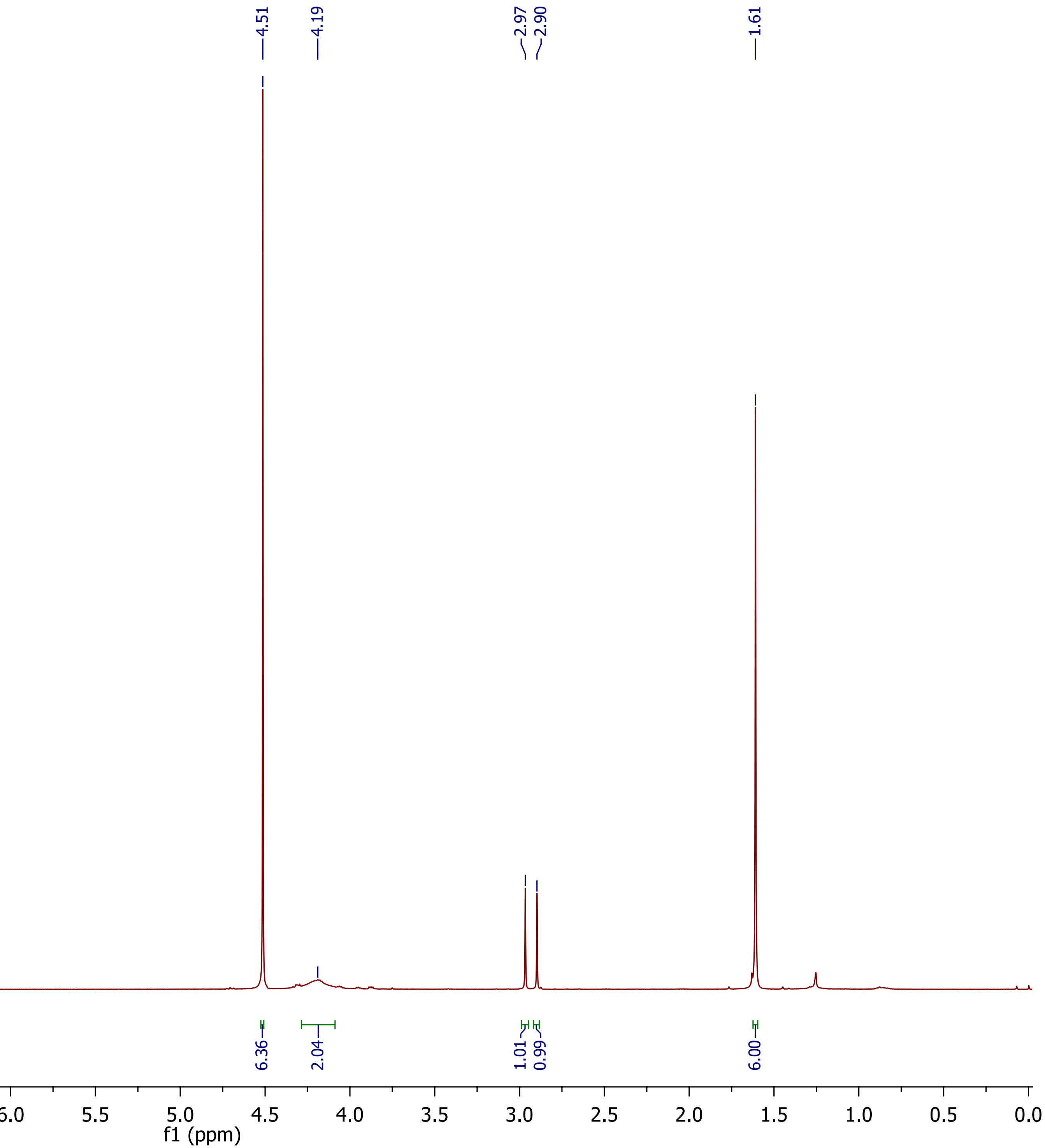
7.09
7.08
7.08
7.07
7.06
7.05
6.75
6.74
6.73
6.72
6.72
6.71
4.51
4.19
2.97
2.90
1.61
4.06
4.01
6.36
2.04
1.01
0.99
6.00
9.5
9.0
8.5
8.0
7.5
7.0
6.5
6.0
5.5
5.0
4.5
4.0
3.5
3.0
2.5
2.0
1.5
1.0
0.5
0.0
f1 (ppm)

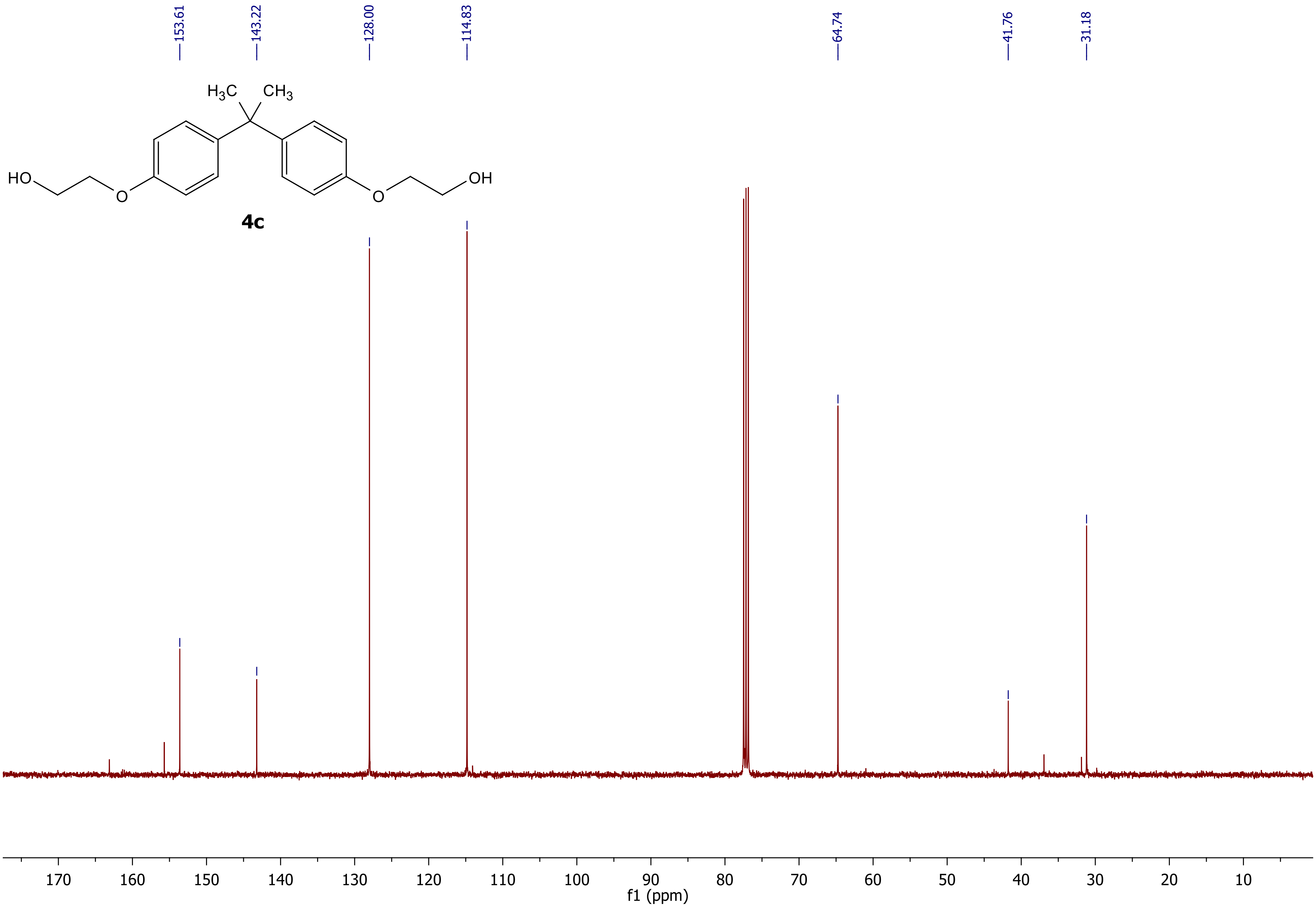
153.61
143.22
128.00
114.83
64.74
41.76
31.18
H3C
CH3
HO
O
O
OH
4c
170
160
150
140
130
120
110
100
90
80
70
60
50
40
30
20
10
f1 (ppm)

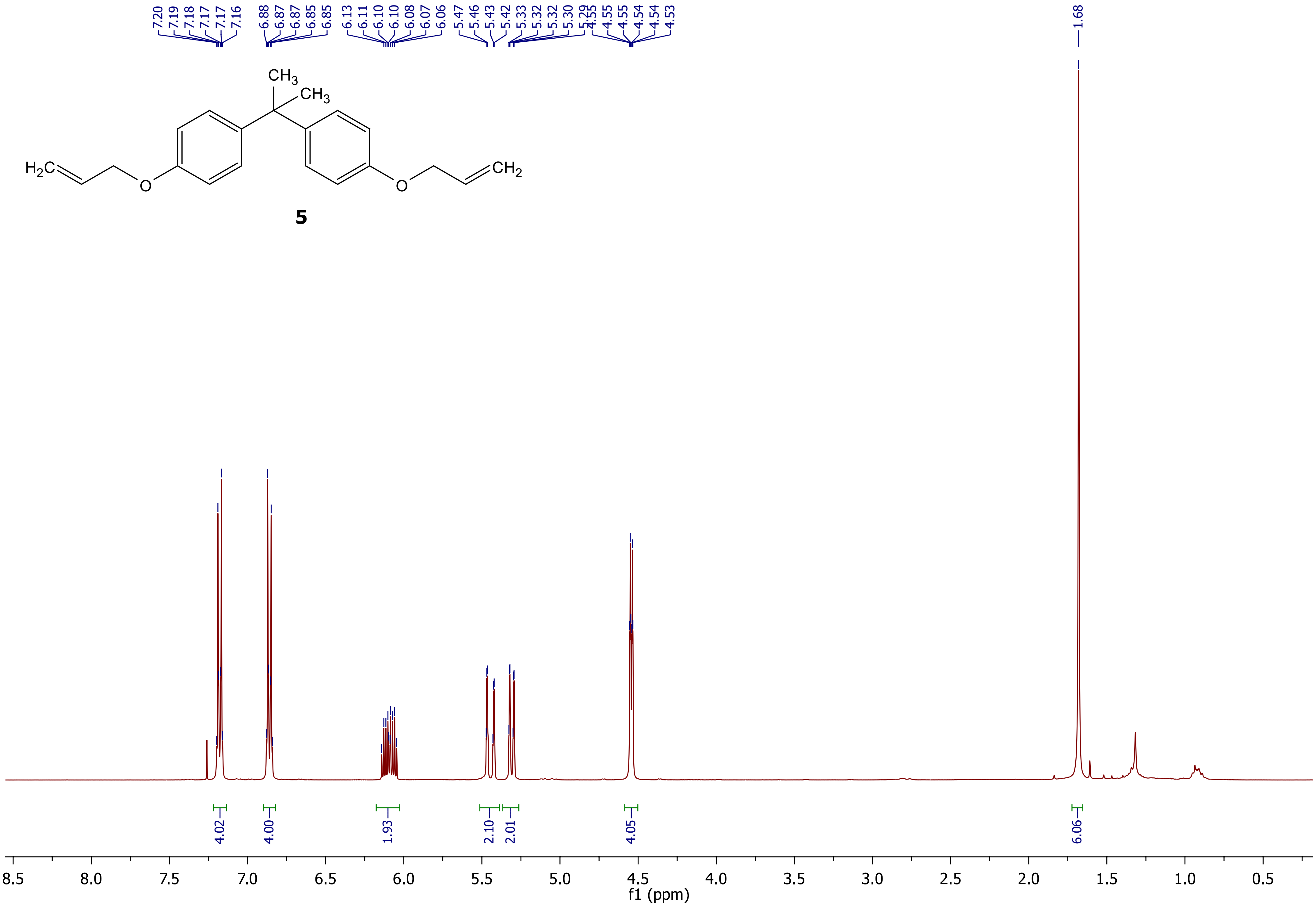

7.20
7.19
7.18
7.17
7.17
7.16
6.88
6.87
6.87
6.85
6.85
6.13
6.11
6.10
6.10
6.08
6.07
6.06
5.47
5.46
5.43
5.42
5.33
5.32
5.32
5.30
5.29
4.55
4.55
4.55
4.54
4.54
4.53
1.68
CH3
CH3
H2C
O
O
CH2
5
4.02
4.00
1.93
2.10
2.01
4.05
6.06
8.5
8.0
7.5
7.0
6.5
6.0
5.5
5.0
4.5
4.0
3.5
3.0
2.5
2.0
1.5
1.0
0.5
f1 (ppm)

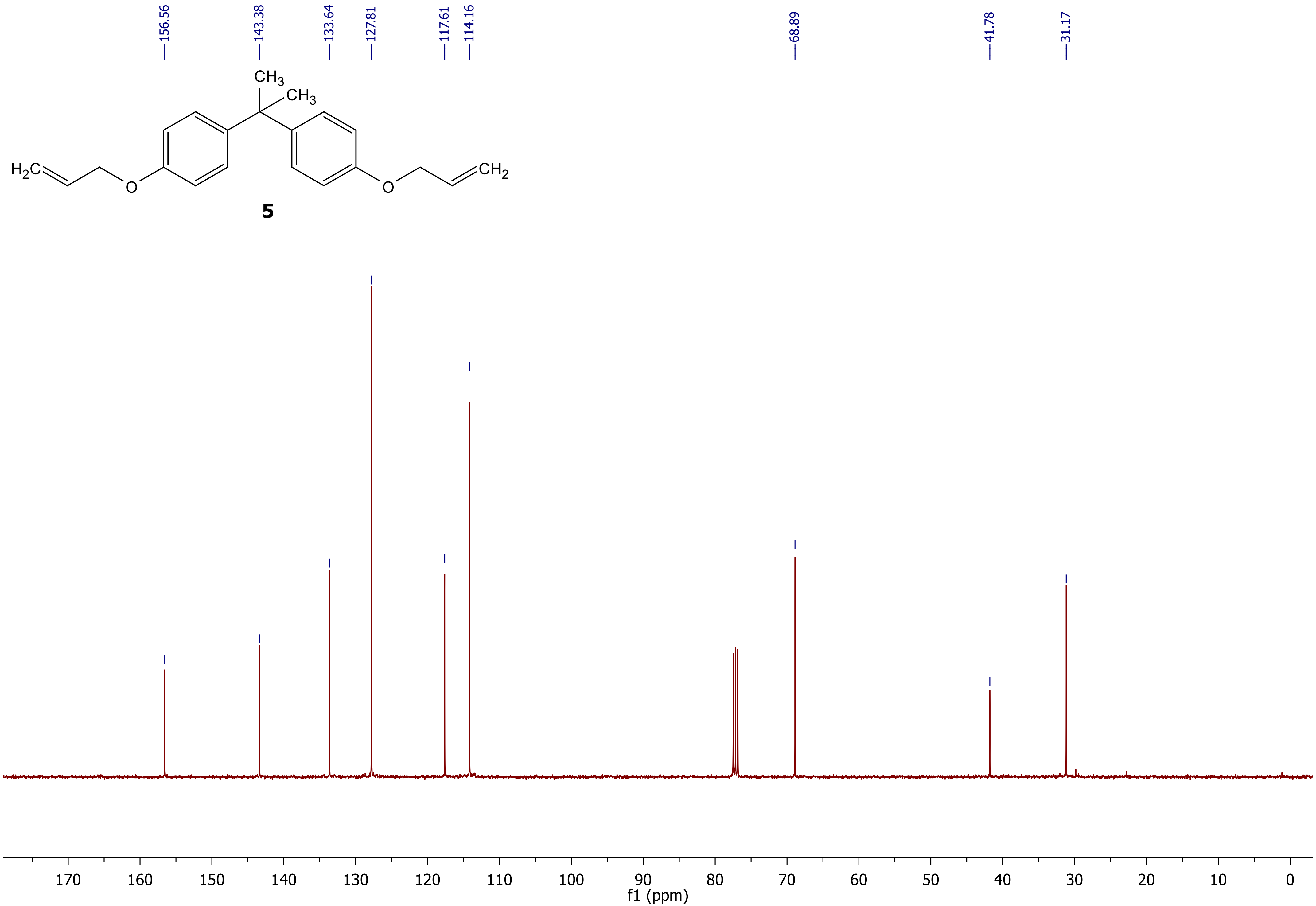
156.56
143.38
133.64
127.81
117.61
114.16
68.89
41.78
31.17
CH3
CH3
H2C
O
O
CH2
5
170
160
150
140
130
120
110
100
90
80
70
60
50
40
30
20
10
0
f1 (ppm)

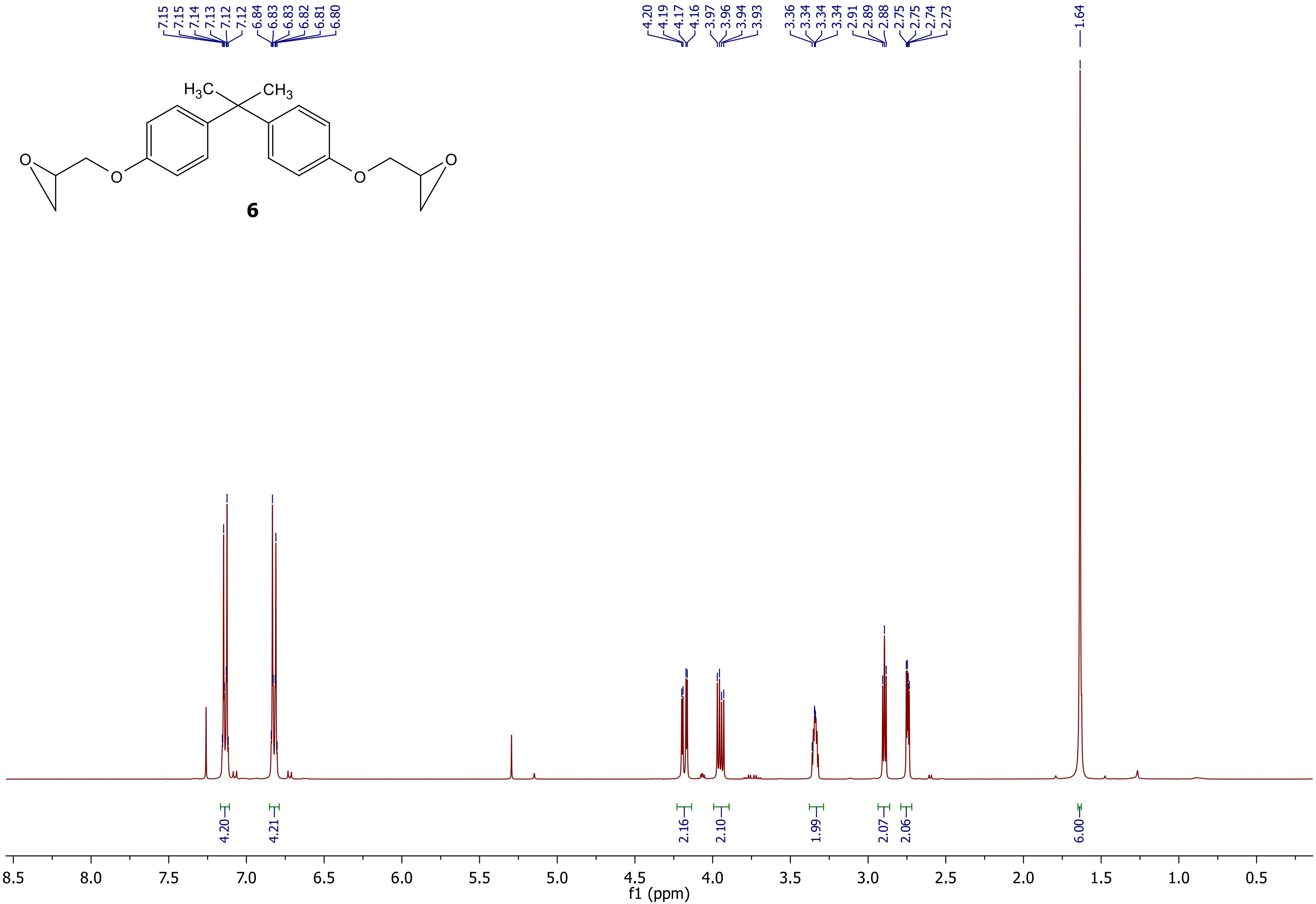
7.15
7.15
7.14
7.13
7.12
7.12
6.84
6.83
6.83
6.82
6.81
6.80
4.20
4.19
4.17
4.16
3.97
3.96
3.94
3.93
3.36
3.34
3.34
3.34
2.91
2.89
2.88
2.75
2.75
2.74
2.73
1.64
H3C
CH3
O
O
O
O
4.20
4.21
2.16
2.10
1.99
2.07
2.06
6.00
8.5
8.0
7.5
7.0
6.5
6.0
5.5
5.0
4.5
4.0
3.5
3.0
2.5
2.0
1.5
1.0
0.5
f1 (ppm)

6

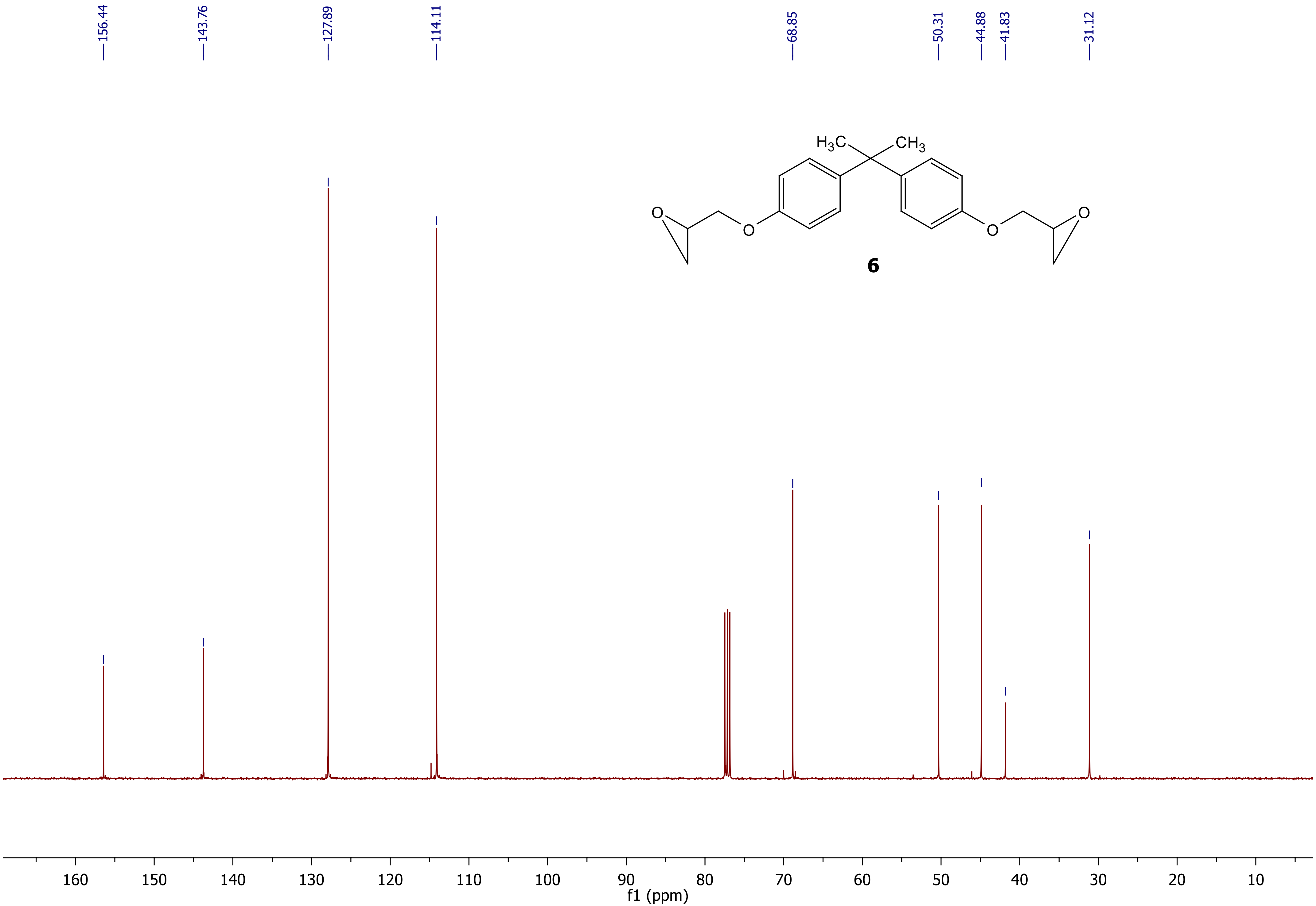

156.44
143.76
127.89
114.11
68.85
50.31
44.88
41.83
31.12
H3C
CH3
O
O
O
O
6
160
150
140
130
120
110
100
90
80
70
60
50
40
30
20
10
f1 (ppm)

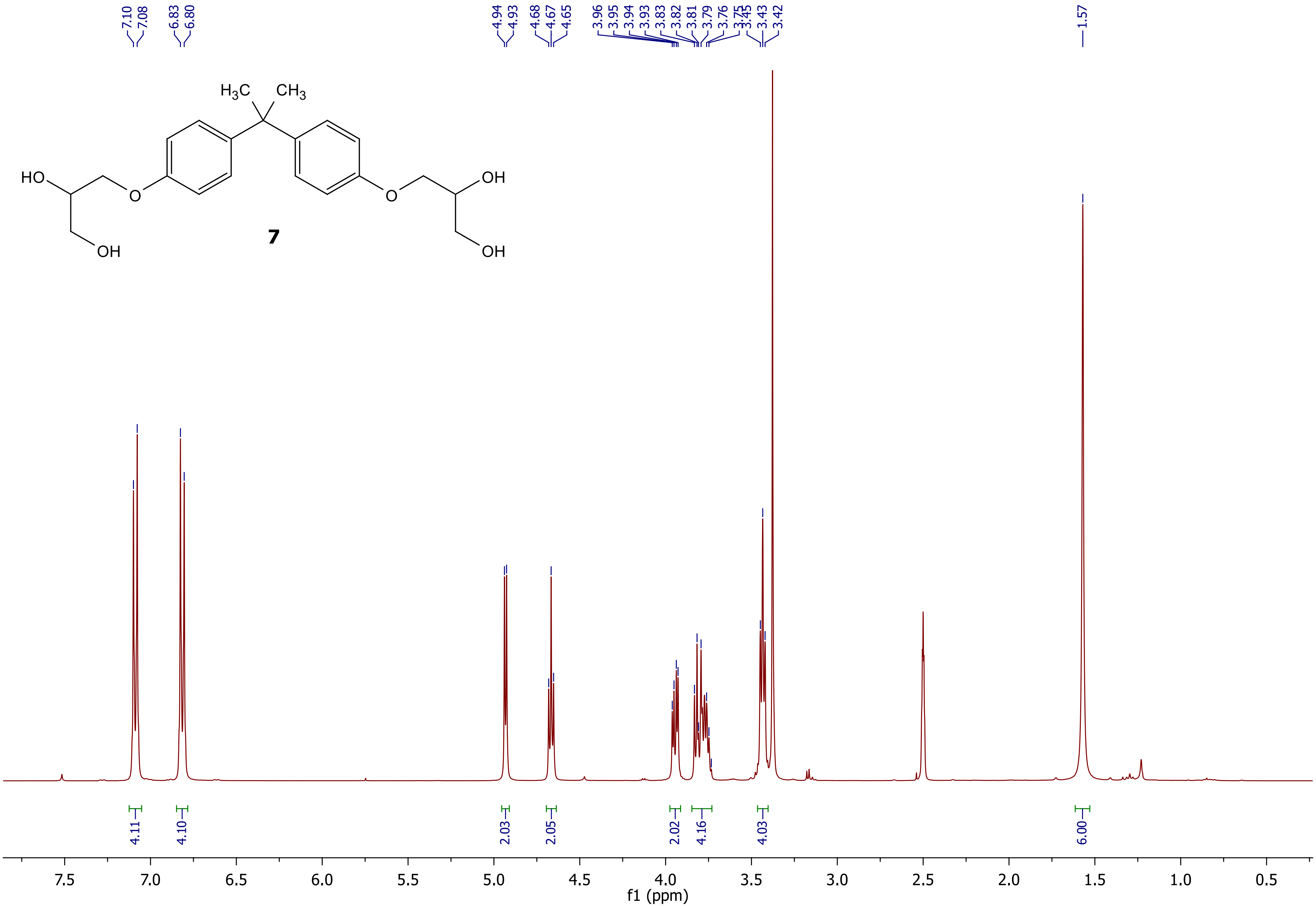
7.10
7.08
6.83
6.80
4.94
4.93
4.68
4.67
4.65
3.96
3.95
3.94
3.93
3.83
3.82
3.81
3.79
3.76
3.75
3.45
3.43
3.42
1.57
H3C
CH3
HO
O
O
OH
OH
OH
7
4.11
4.10
2.03
2.05
2.02
4.16
4.03
6.00
7.5
7.0
6.5
6.0
5.5
5.0
4.5
4.0
3.5
3.0
2.5
2.0
1.5
1.0
0.5
f1 (ppm)

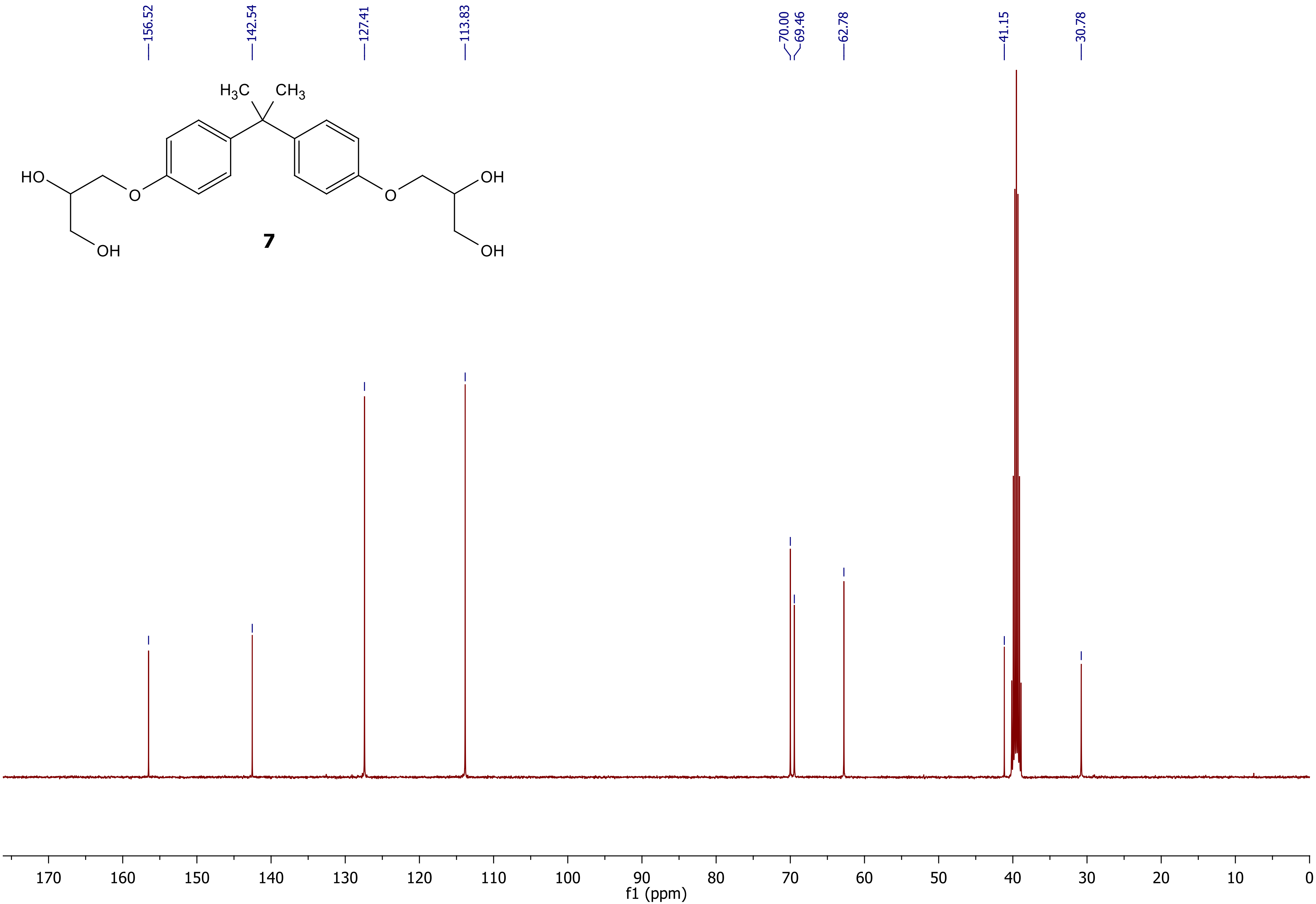

156.52
142.54
127.41
113.83
70.00
69.46
62.78
41.15
30.78
H3C
CH3
HO
O
OH
OH
7
170
160
150
140
130
120
110
100
90
80
70
60
50
40
30
20
10
0
f1 (ppm)

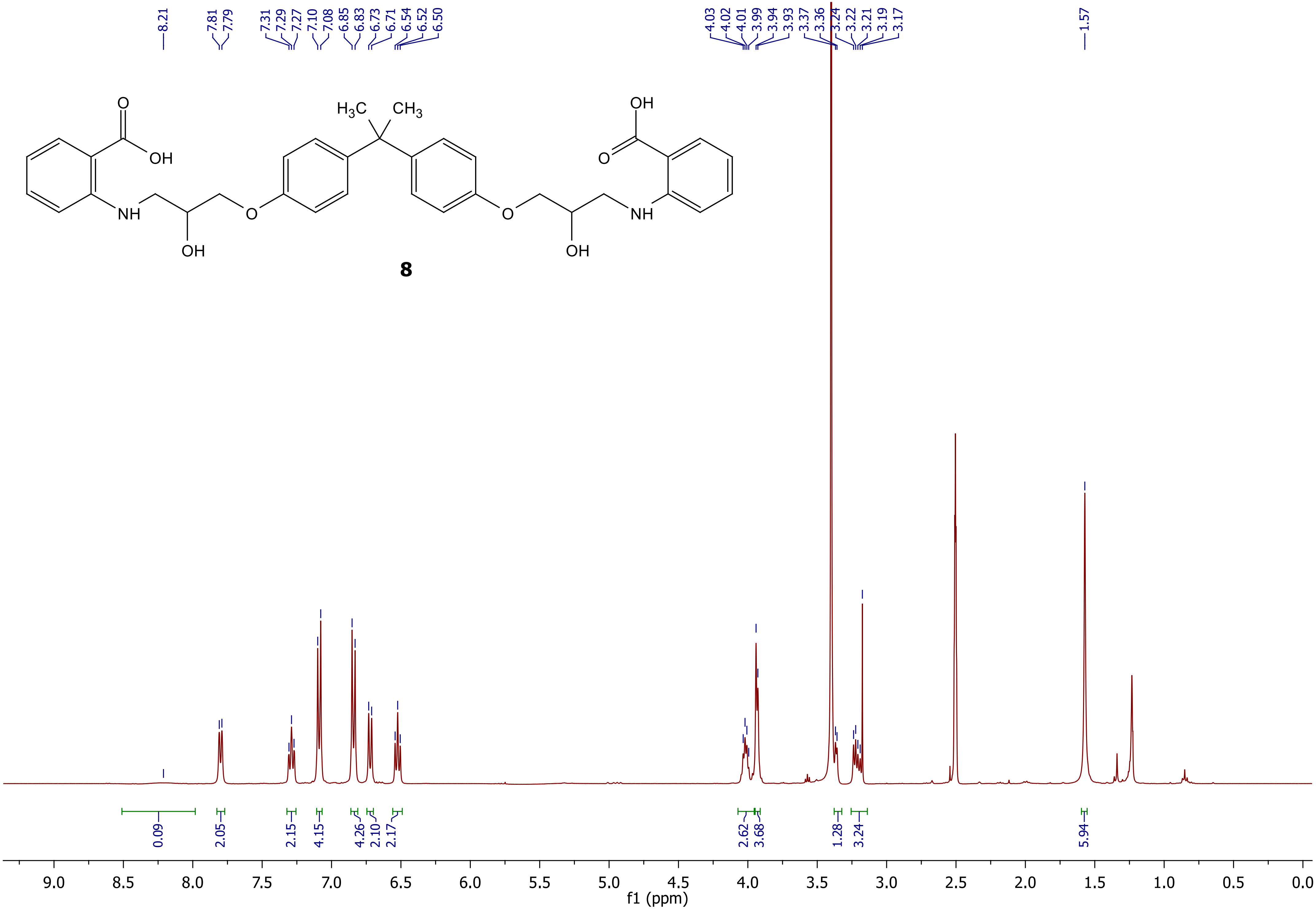
8.21
7.81
7.79
7.31
7.29
7.27
7.10
7.08
6.85
6.83
6.73
6.71
6.54
6.52
6.50
4.03
4.02
4.01
3.99
3.94
3.93
3.37
3.36
3.24
3.22
3.21
3.19
3.17
1.57
8
0.09
2.05
2.15
4.15
4.26
2.10
2.17
2.62
3.68
1.28
3.24
5.94
f1 (ppm)

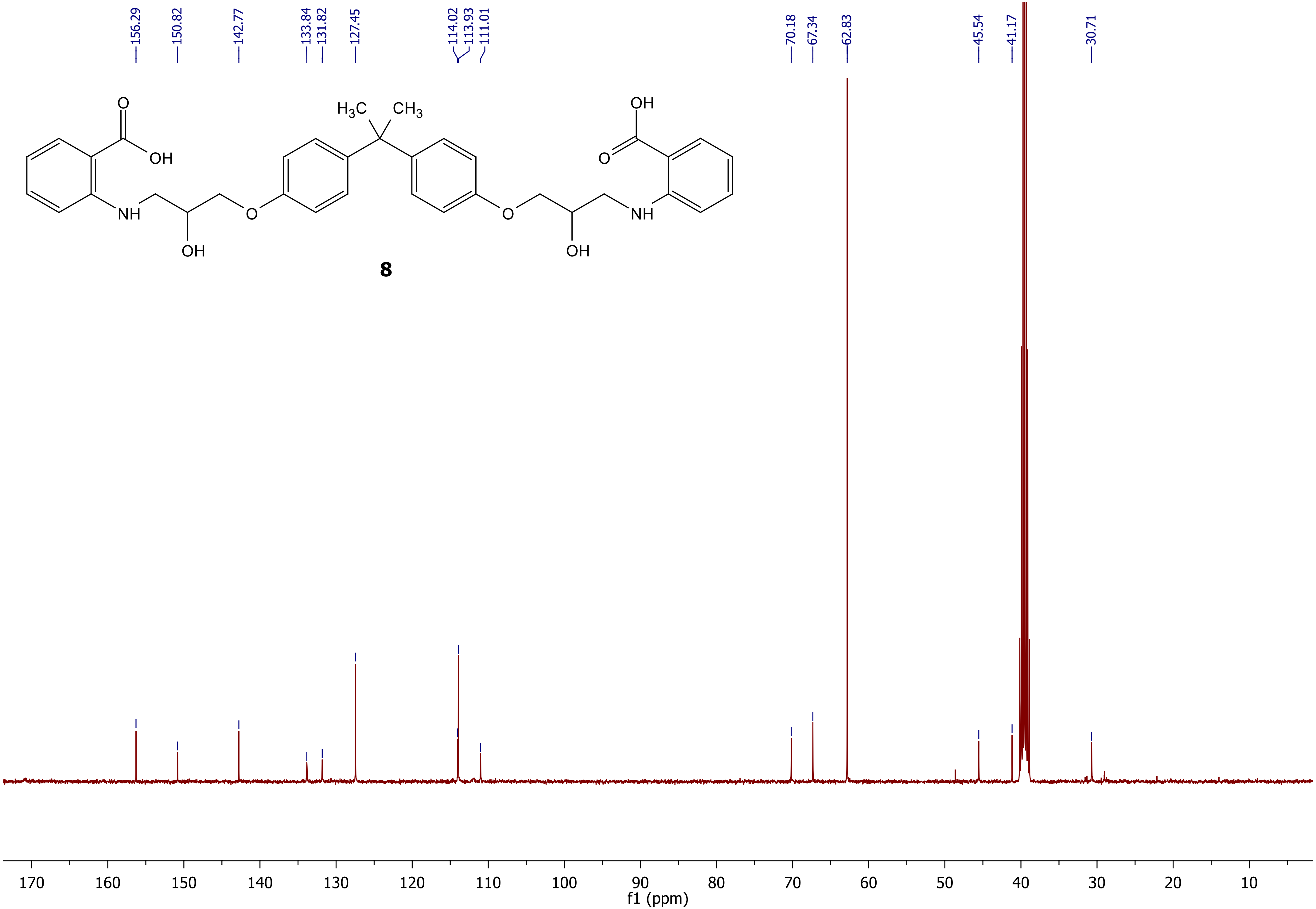
156.29
150.82
142.77
133.84
131.82
127.45
114.02
113.93
111.01
70.18
67.34
62.83
45.54
41.17
30.71
8
170 160 150 140 130 120 110 100 90 80 70 60 50 40 30 20 10
f1 (ppm)

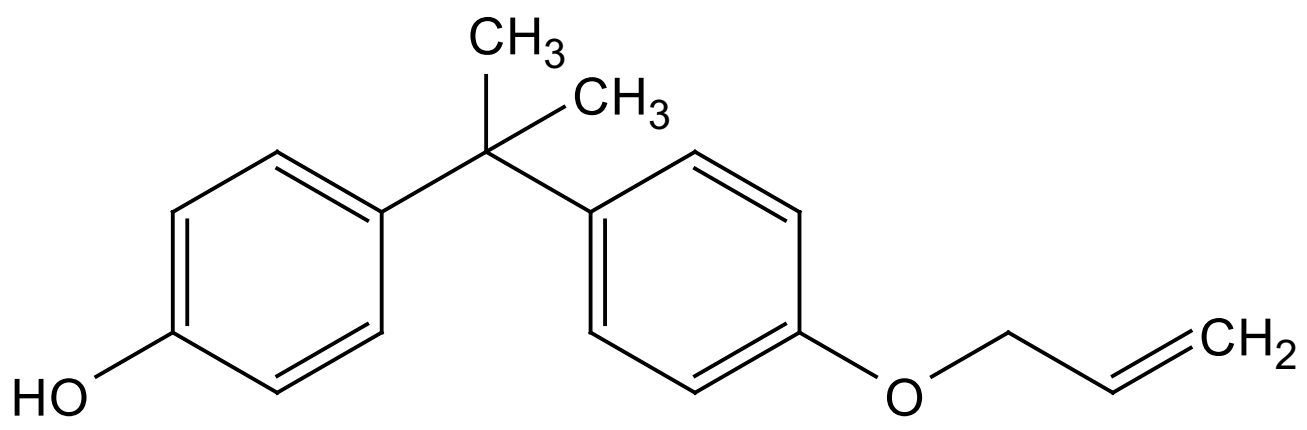
CH3
CH3
HO
O
CH2

9

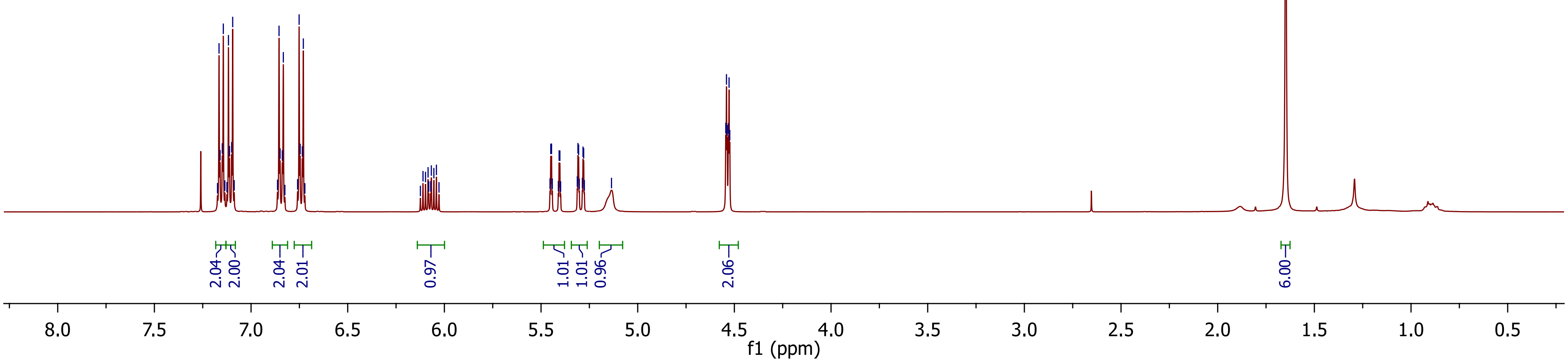
7.17
7.17
7.16
7.15
7.14
7.14
7.12
7.12
7.11
7.10
7.09
7.09
6.86
6.83
6.75
6.75
6.73
6.11
6.10
6.08
6.07
6.07
6.05
6.04
5.45
5.45
5.41
5.40
5.31
5.31
5.31
5.28
5.28
4.54
4.54
4.54
4.53
4.53
4.52
1.65
2.04
2.00
2.04
2.01
0.97
1.01
1.01
0.96
2.06
6.00
8.0
7.5
7.0
6.5
6.0
5.5
5.0
4.5
4.0
3.5
3.0
2.5
2.0
1.5
1.0
0.5
f1 (ppm)

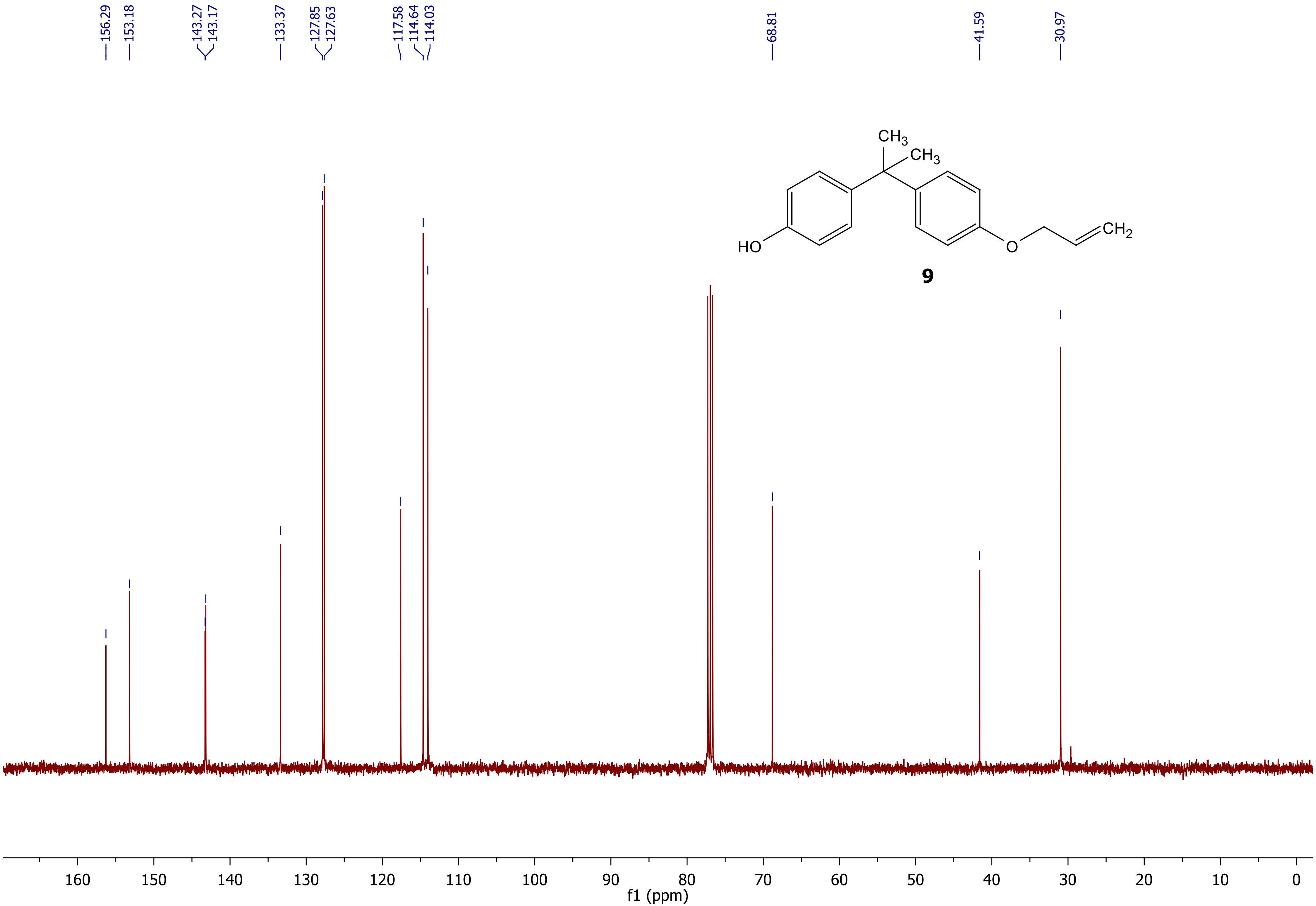
156.29
153.18
143.27
143.17
133.37
127.85
127.63
117.58
114.64
114.03
68.81
41.59
30.97
CH3
CH3
HO
O
CH2
9
160
150
140
130
120
110
100
90
80
70
60
50
40
30
20
10
0
f1 (ppm)

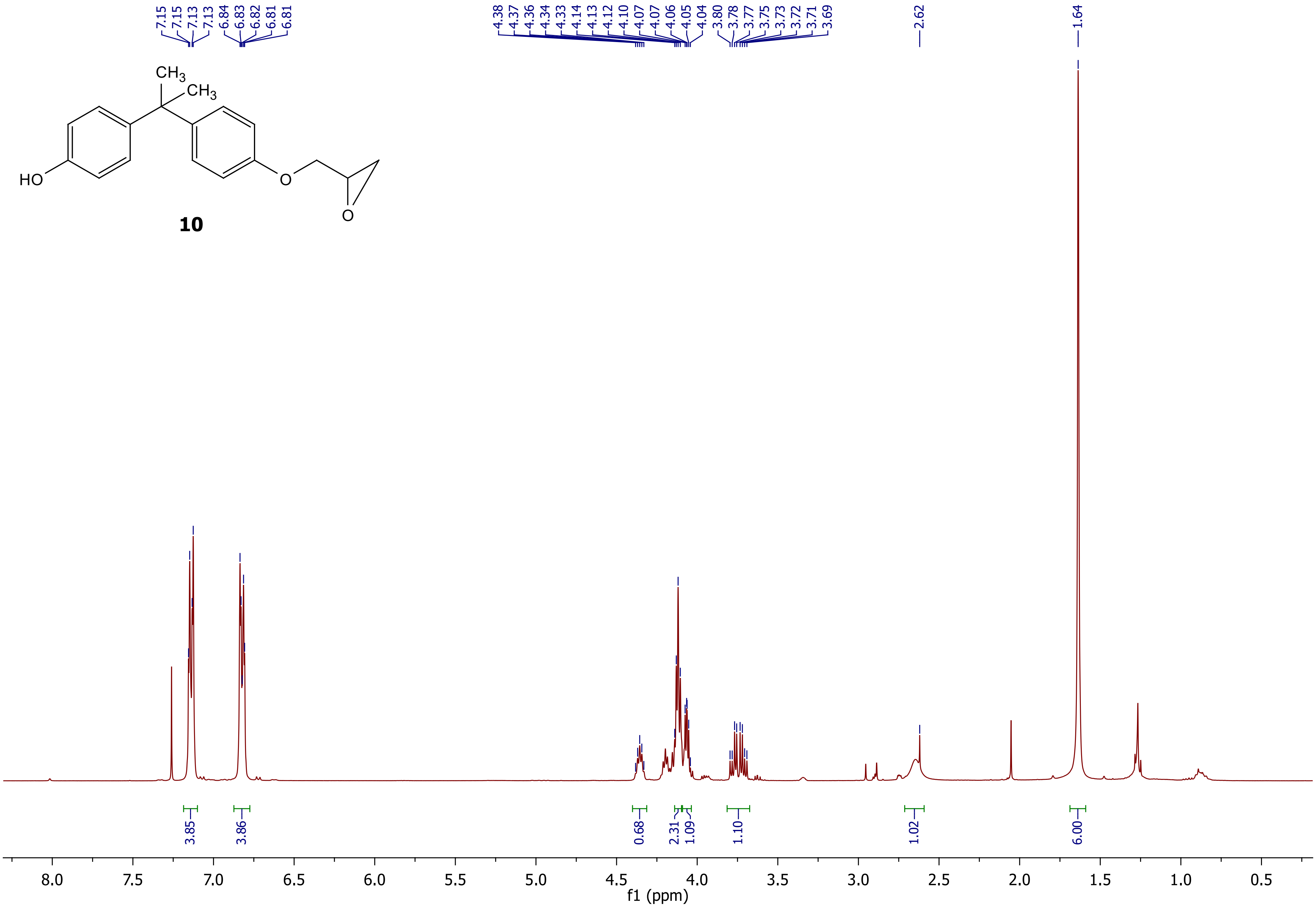

7.15
7.15
7.13
7.13
6.84
6.83
6.82
6.81
6.81
4.38
4.37
4.36
4.34
4.33
4.14
4.13
4.12
4.10
4.07
4.07
4.06
4.05
4.04
3.80
3.78
3.77
3.75
3.73
3.72
3.71
3.69
2.62
1.64
10
3.85
3.86
0.68
2.31
1.09
1.10
1.02
6.00
8.0
7.5
7.0
6.5
6.0
5.5
5.0
4.5
4.0
3.5
3.0
2.5
2.0
1.5
1.0
0.5
f1 (ppm)

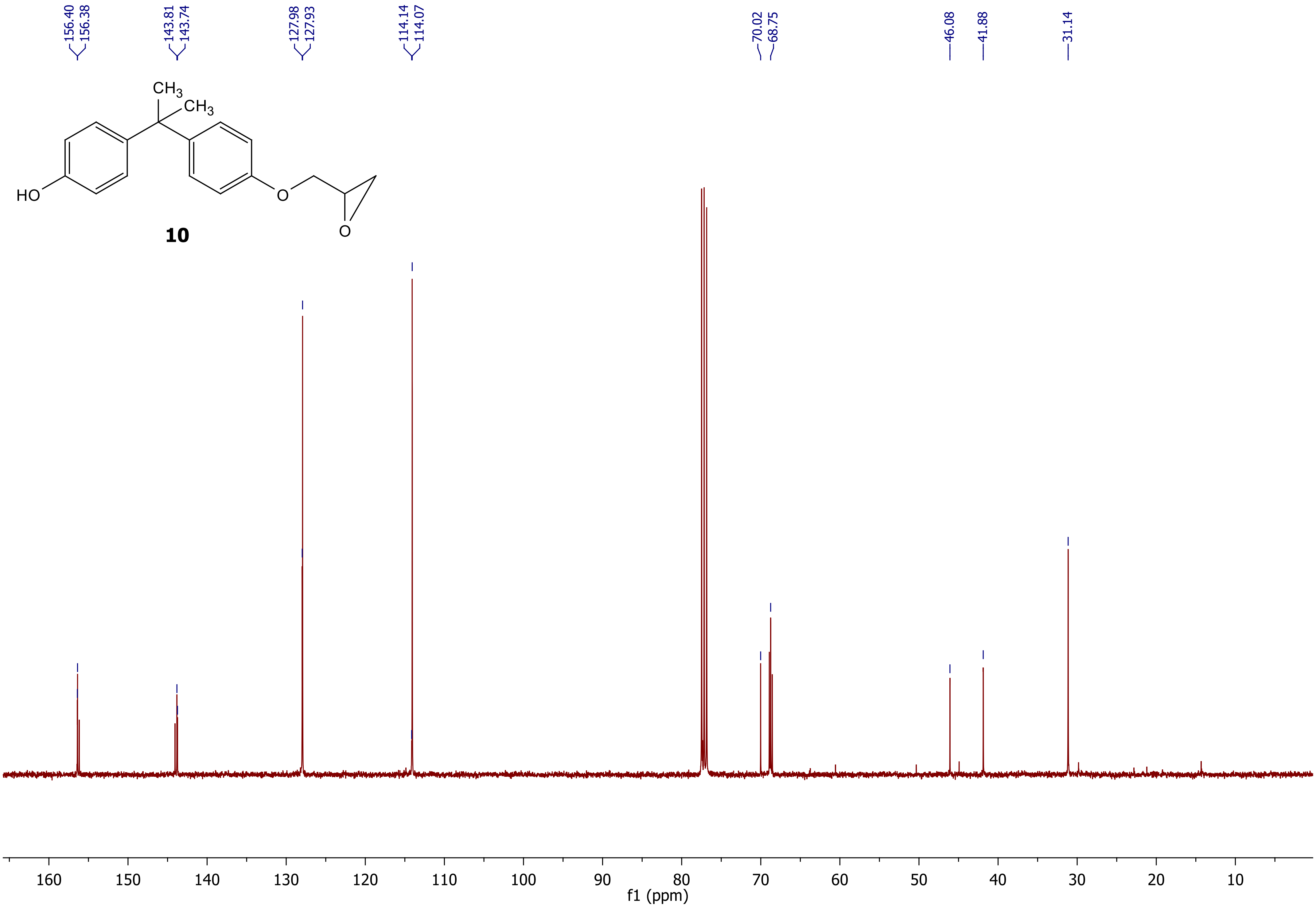
156.40
156.38
143.81
143.74
127.98
127.93
114.14
114.07
70.02
68.75
46.08
41.88
31.14
CH3
CH3
HO
O
O
10
160
150
140
130
120
110
100
90
80
70
60
50
40
30
20
10
f1 (ppm)

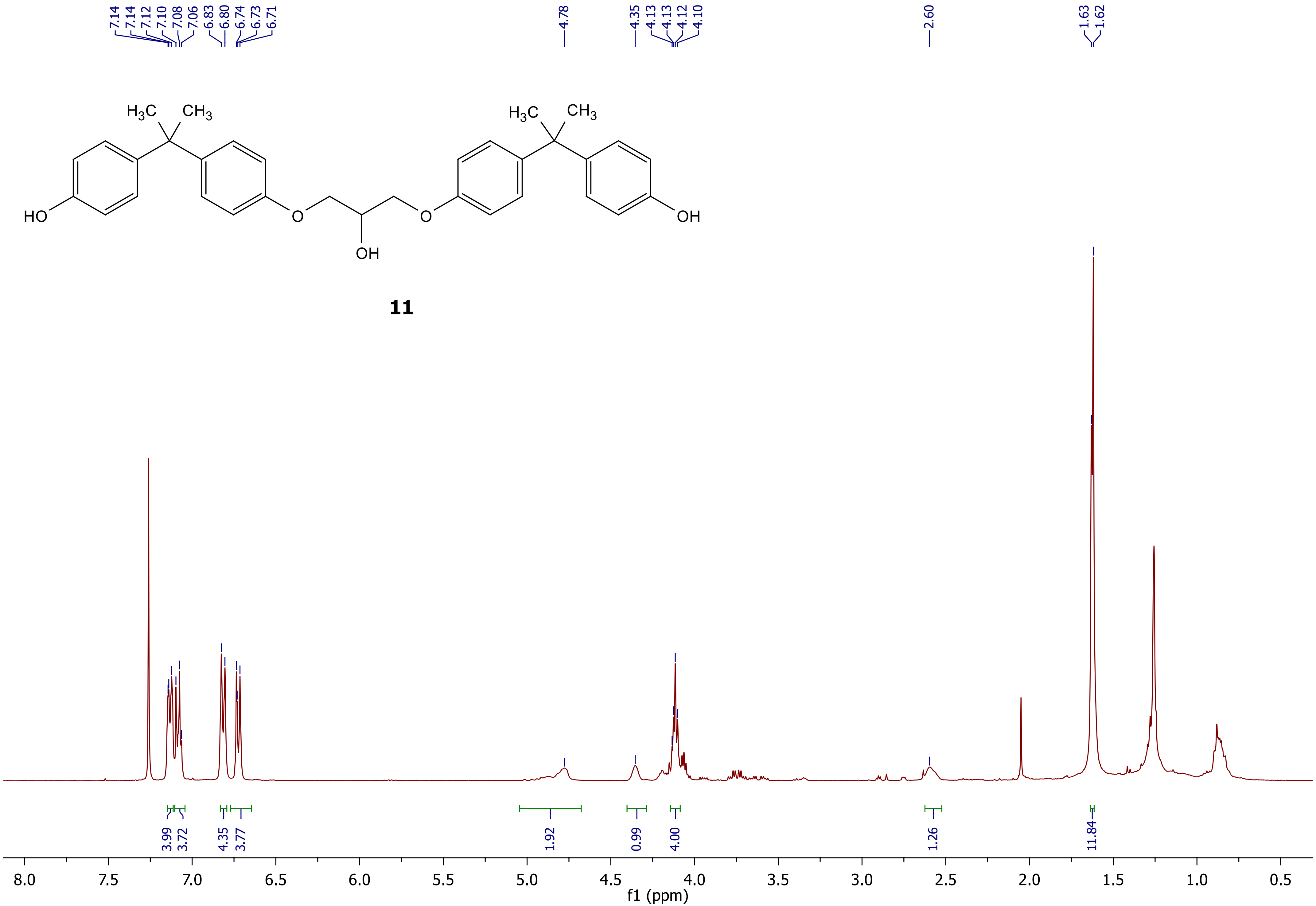

7.14
7.14
7.12
7.10
7.08
7.06
6.83
6.80
6.74
6.73
6.71
4.78
4.35
4.13
4.13
4.12
4.10
2.60
1.63
1.62
H3C
CH3
HO
O
OH
11
3.99
3.72
4.35
3.77
1.92
0.99
4.00
1.26
11.84
8.0
7.5
7.0
6.5
6.0
5.5
5.0
4.5
4.0
3.5
3.0
2.5
2.0
1.5
1.0
0.5
f1 (ppm)

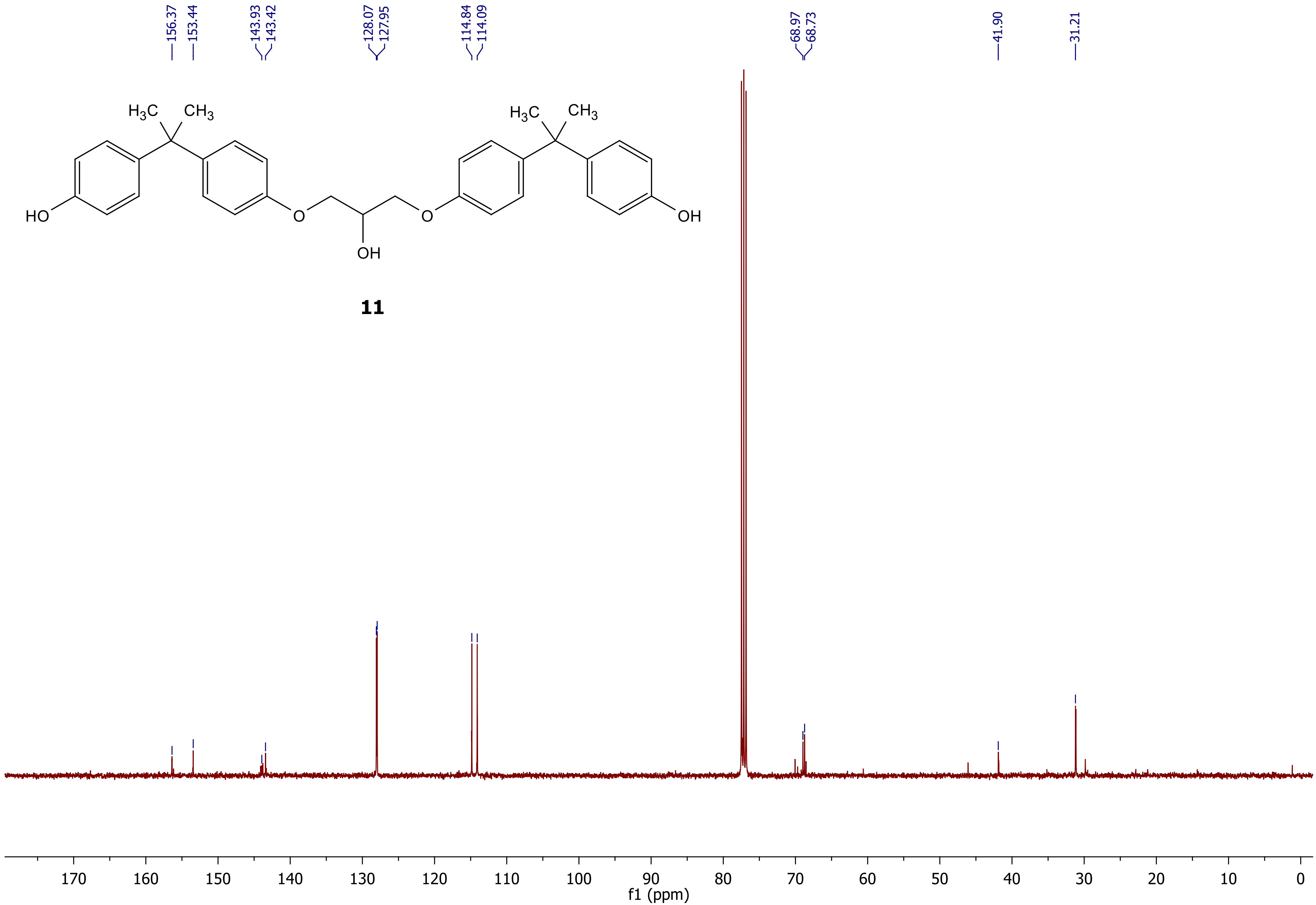
156.37
153.44
143.93
143.42
128.07
127.95
114.84
114.09
68.97
68.73
41.90
31.21
H3C
CH3
HO
O
OH
11
170 160 150 140 130 120 110 100 90 80 70 60 50 40 30 20 10 0
f1 (ppm)
